# Molecular characterization of the RNA-protein complex directing −2/−1 programmed ribosomal frameshifting during arterivirus replicase expression

**DOI:** 10.1074/jbc.RA120.016105

**Published:** 2021-01-13

**Authors:** Ankoor Patel, Emmely E. Treffers, Markus Meier, Trushar R. Patel, Jörg Stetefeld, Eric J. Snijder, Brian L. Mark

**Affiliations:** 1 Department of Microbiology, University of Manitoba, Winnipeg, Manitoba, Canada; 2 Molecular Virology Laboratory, Department of Medical Microbiology, Leiden University Medical Center, Leiden, The Netherlands; 3 Department of Chemistry, University of Manitoba, Winnipeg, Manitoba, Canada; 4 Alberta RNA Research and Training Institute, Department of Chemistry and Biochemistry, University of Lethbridge, Lethbridge, Alberta, Canada

**Keywords:** PRRSV, nidovirus, poly(C)-binding protein, PCPB2, nonstructural protein 1, β, nsp1, β-, small-angle X-ray scattering, sedimentation velocity, RNA, virology, ribosome, translation, structural biology, viral replication, RNA virus, ribosome function, nsp1beta, PCBP

## Abstract

Programmed ribosomal frameshifting (PRF) is a mechanism used by arteriviruses like porcine reproductive and respiratory syndrome virus (PRRSV) to generate multiple proteins from overlapping reading frames within its RNA genome. PRRSV employs −1 PRF directed by RNA secondary and tertiary structures within its viral genome (canonical PRF), as well as a noncanonical −1 and −2 PRF that are stimulated by the interactions of PRRSV nonstructural protein 1β (nsp1β) and host protein poly(C)-binding protein (PCBP) 1 or 2 with the viral genome. Together, nsp1β and one of the PCBPs act as transactivators that bind a C-rich motif near the shift site to stimulate −1 and −2 PRF, thereby enabling the ribosome to generate two frameshift products that are implicated in viral immune evasion. How nsp1β and PCBP associate with the viral RNA genome remains unclear. Here, we describe the purification of the nsp1β:PCBP2:viral RNA complex on a scale sufficient for structural analysis using small-angle X-ray scattering and stochiometric analysis by analytical ultracentrifugation. The proteins associate with the RNA C-rich motif as a 1:1:1 complex. The monomeric form of nsp1β within the complex differs from previously reported homodimer identified by X-ray crystallography. Functional analysis of the complex via mutational analysis combined with RNA-binding assays and cell-based frameshifting reporter assays reveal a number of key residues within nsp1β and PCBP2 that are involved in complex formation and function. Our results suggest that nsp1β and PCBP2 both interact directly with viral RNA during formation of the complex to coordinate this unusual PRF mechanism.

RNA viruses have evolved remarkable noncanonical translational mechanisms to maximize the coding capacity of their genomes (1, 2), including the use of programmed ribosomal frameshifting (PRF). PRF enables the ribosome to access multiple overlapping ORFs within the viral genome (1, 3, 4), thus yielding alternative viral protein variants from what—upon cursory inspection—appears to be a single gene, allowing for the expression of partially colinear proteins with alternate C-terminal extensions and domains (1, 5, 6).

The first evidence for the occurrence of PRF was discovered in Rous sarcoma virus, which produces a gag-pol fusion protein from briefly overlapping gag and pol ORFs during infection (7, 8, 9). This is achieved by causing the host cell ribosome to slip back one position (−1 PRF) during translation of the viral RNA genome, which occurs at a heptameric “slippery” sequence that is located 5–10 nucleotides upstream of an RNA structural element (stem-loop or pseudoknot) (7, 8, 9). Encountering this RNA structure causes the ribosome to pause and “slip” on the slippery sequence, resulting in a −1 frameshift that opens access to an alternate reading frame (10). The frequency of frameshifting events differs per virus and presumably controls the stoichiometry of certain viral proteins (1).

Members of the order *Nidovirales* (including among others the families *Arteriviridae* and *Coronaviridae*) encode two large replicase polyproteins, pp1a and pp1ab, which are post-translationally cleaved to yield 12–16 mature nonstructural proteins (nsps) (11). Expression of pp1ab depends on a pseudoknot-stimulated −1 PRF event to occur in the short ORF1a/ORF1b overlap region (5, 12). Next to this well-characterized −1 PRF event, most members of the arterivirus family also employ a more unusual −2 PRF mechanism. For example, in porcine reproductive and respiratory syndrome virus (PRRSV) and simian hemorrhagic fever virus (SHFV), −1 and −2 PRF events were shown to occur at the same site in the nsp2-coding region of ORF1a, yielding two nsp2 variants. In the case of PRRSV (Fig. 1*A*), these products are either truncated compared with full-length nsp2 (nsp2N, resulting from −1 PRF) or contain an alternative C-terminal domain (nsp2TF, resulting form −2 PRF) and were implicated in suppressing host innate immune responses (Fig. 1*A*) (13, 14, 15, 16). Interestingly, whereas a characteristic slippery sequence is present in the region of the PRRSV genome where these frameshifts occur, no discernible RNA secondary structural element could be predicted (14). However, a highly conserved C-rich motif (CCCANCUCC, or similar) is found 11 nt downstream of the slippery sequence shift site in studied PRRSV isolates (Fig. 1*B*), which suggested that a novel transactivating mechanism facilitates PRF at this position as opposed to a ribosomal pausing mechanism that is usually induced by an RNA tertiary structural element (13, 17). Indeed, two *trans*-acting elements subsequently were shown to control −1 and −2 PRF in PRRSV: the PRRSV protein nsp1β and the host cell protein poly(C)-binding protein 1 or 2 (PCBP1 or -2) (13, 14, 17). The two proteins interact with each other and with the viral RNA genome to induce −1/−2 PRF in the nsp2-coding region of ORF1a. Although PCBP2 and nsp1β had previously been shown to interact with each other (18), the significance of this interaction for efficient PRF has only recently been discovered (13, 17).

**Figure 1:**
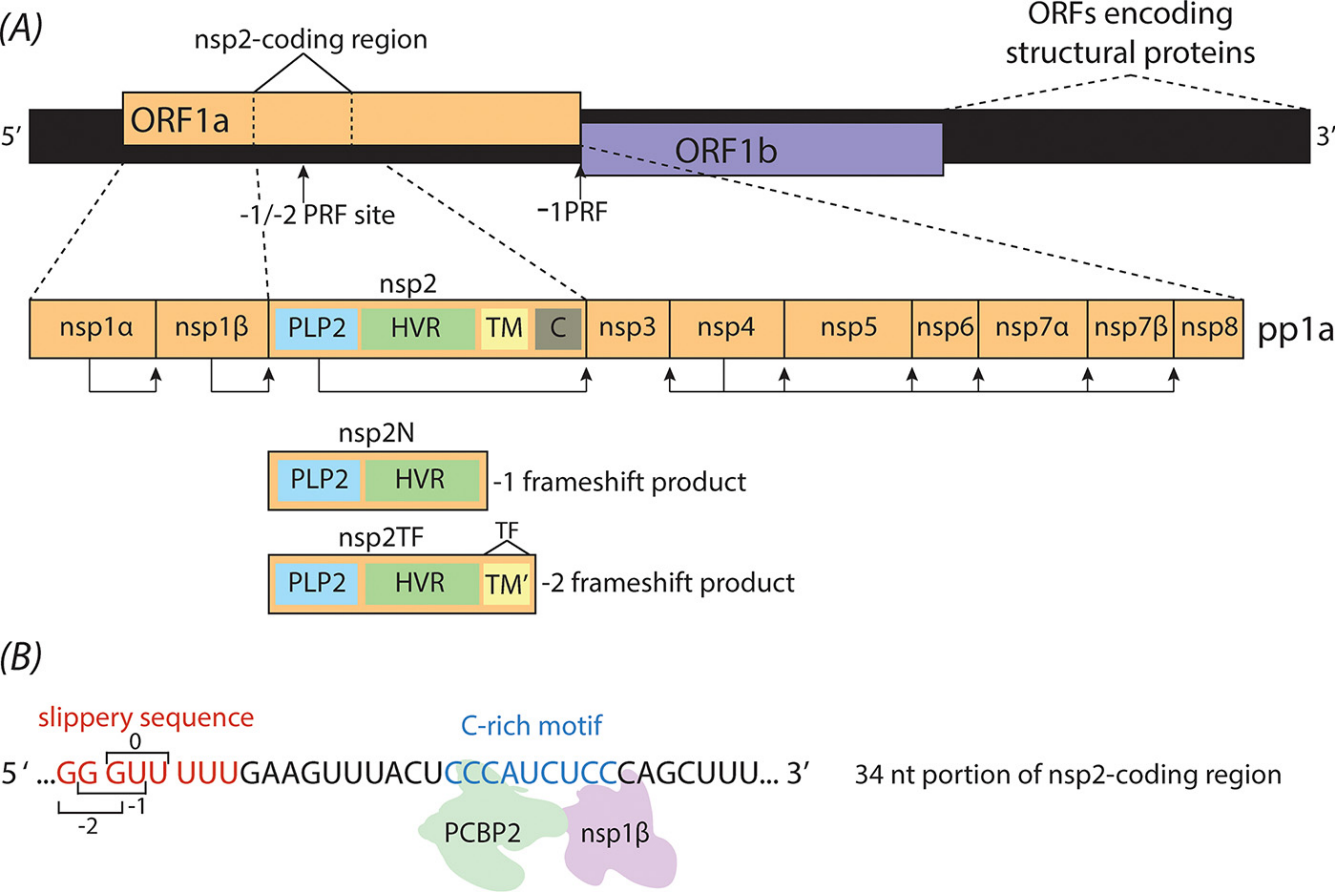
**Organization of the PRRSV genome showing known sites of PRF.***A*, schematic of the full +ssRNA PRRSV genome. Translation of the largest ORFs (ORF1a and ORF1b) yields replicase polyproteins 1a and 1ab (pp1a and pp1ab). Translation of ORF1b requires a −1 PRF event at the end of ORF1a. PRRSV pp1a is comprised of 10 nonstructural protein (nsp) subunits, four of which have autocatalytic polyprotein cleavage activity (*arrows* indicate cleavage sites). Whereas the –1 PRF at the ORF1a/1b junction is directed by stimulatory RNA structures (5) text, additional −1 and −2 PRF events occurring within the nsp2-coding region do not depend on higher-order RNA structures (13, 14). These PRF events result in truncated nsp2 variants, nsp2N and nsp2TF, which both retain the papain-like cysteine protease (PLP2) and hypervariable regions (*HVR*) but lack the C-terminal Cys-rich domain (*C*). Nsp2TF also contains a modified transmembrane domain (*TM*′) that is encoded by a short alternative ORF (*TF*) that overlaps with ORF1a in the −2 reading frame. *B*, the region of ORF1a from PRRSV SD01-08 showing where −1/−2 PRF occurs within nsp2. The slippery sequence is shown in *red*, and the C-rich motif is shown in *blue*. The C-rich motif replaces the canonical higher-order RNA structural element as found in most other PRF mechanisms and serves as a putative binding site for nsp1β and PCBP2. Adapted from Ref. 13.

PRRSV remains the most economically important viral disease in the swine industry (19), and its high pathogenicity may be due, in part, to the immune evasion mechanisms it employs during infection (11). Consequently, the further dissection of its molecular biology and gene expression mechanisms is highly relevant for efforts to improve PRRSV vaccines, including those based on attenuation by targeted engineering of the viral genome (20). Despite its importance to PRRSV replication, the biochemistry and structural biology of the interactions between nsp1β, PCBP1 or -2, and the PRRSV RNA genome have not been explored. Here we provide structural and functional insights into the quaternary complex between nsp1β:PCBP2 and viral RNA that controls PRF. Site-directed mutations in both nsp1β and PCBP2 pinpointed key residues needed for complex formation with the RNA genome. Nsp1β mutagenesis was also used to identify residues essential to stimulate PRF as well as residues involved in the evasion of innate immune responses. Whereas we found nsp1β and PCBP2 to be unstable on their own, combining the proteins with viral RNA containing the putative slippery sequence and C-rich motif resulted in a highly stable complex that we could study by analytical ultracentrifugation and small-angle X-ray scattering. Our study provides detailed molecular insights into a novel PRF-directing mechanism employing two protein transactivators interacting with the PRRSV genome to expand its coding capacity.

## Results and discussion

### Expression and purification of nsp1β and PCPB2

**Figure 2:**
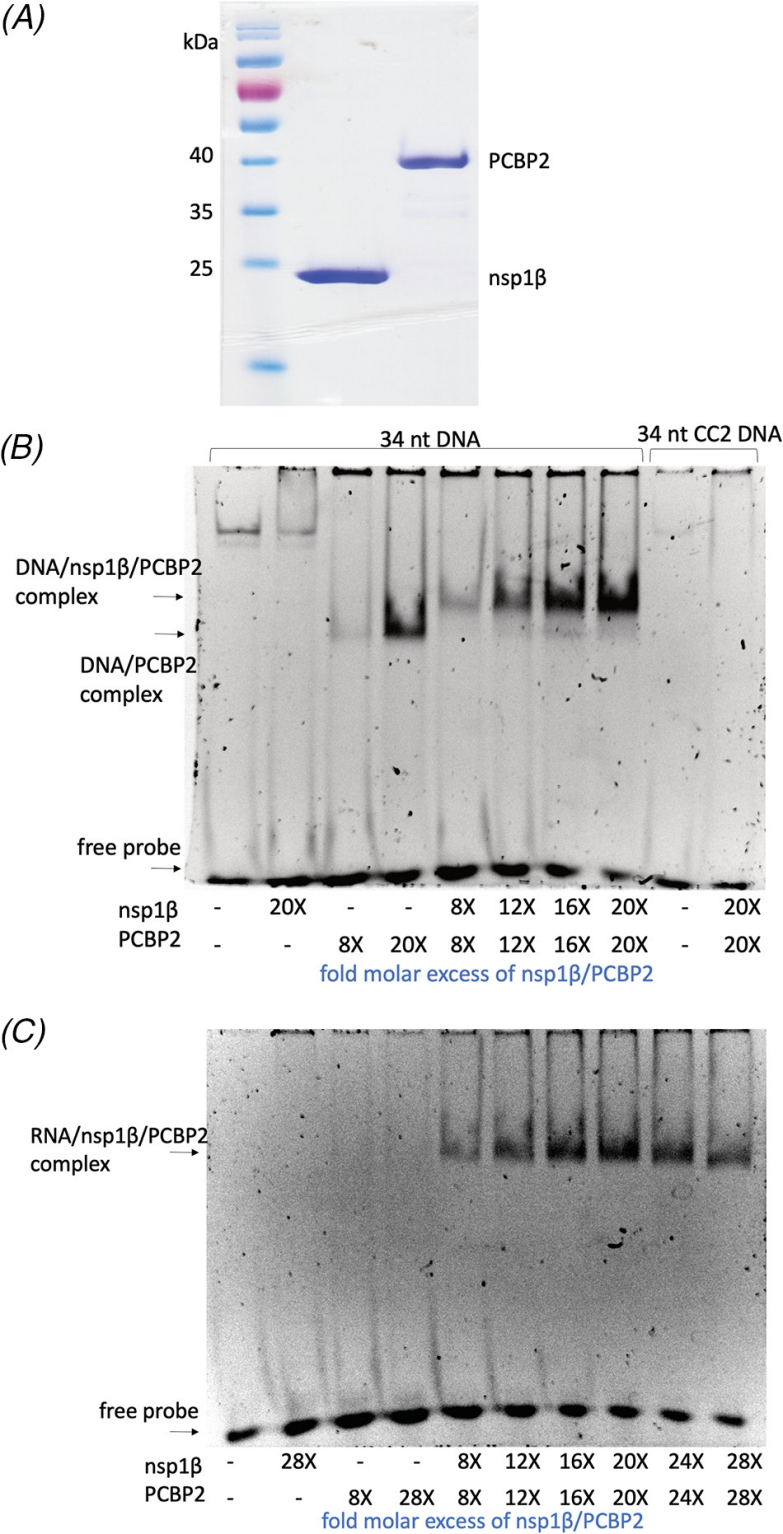
**Purification of nsp1β and PCBP2 and their interaction with DNA and RNA.***A*, SDS-PAGE showing recombinant PRRSV SD01-08 nsp1β (∼23 kDa) and human PCBP2 (∼38 kDa). *B*, EMSA performed with a 20 μm concentration of a 34-nt ssDNA probe corresponding to a stretch of the nsp2-coding region of the PRRSV SD01-08 ssRNA genome where PRF occurs (Fig. 1*B*). Nsp1β and PCBP2 were combined independently or in tandem with the nucleic acid probe. The molar excess of each protein relative to the nucleic acid probe is shown *below* each *lane*. *Lanes 9* and *10* contain a control DNA probe (*CC2*) (14) in which the C-rich region has been altered to adenine/guanine nucleobases. *C*, EMSA performed with a 20 μm concentration of a 34-nt ssRNA probe identical to a stretch of the nsp2-coding region of the PRRSV ssRNA genome where PRF occurs. Nsp1β and PCBP2 were combined independently or in tandem. The molar excess of each protein relative to the RNA probe is shown *below* each well.

**Figure 3:**
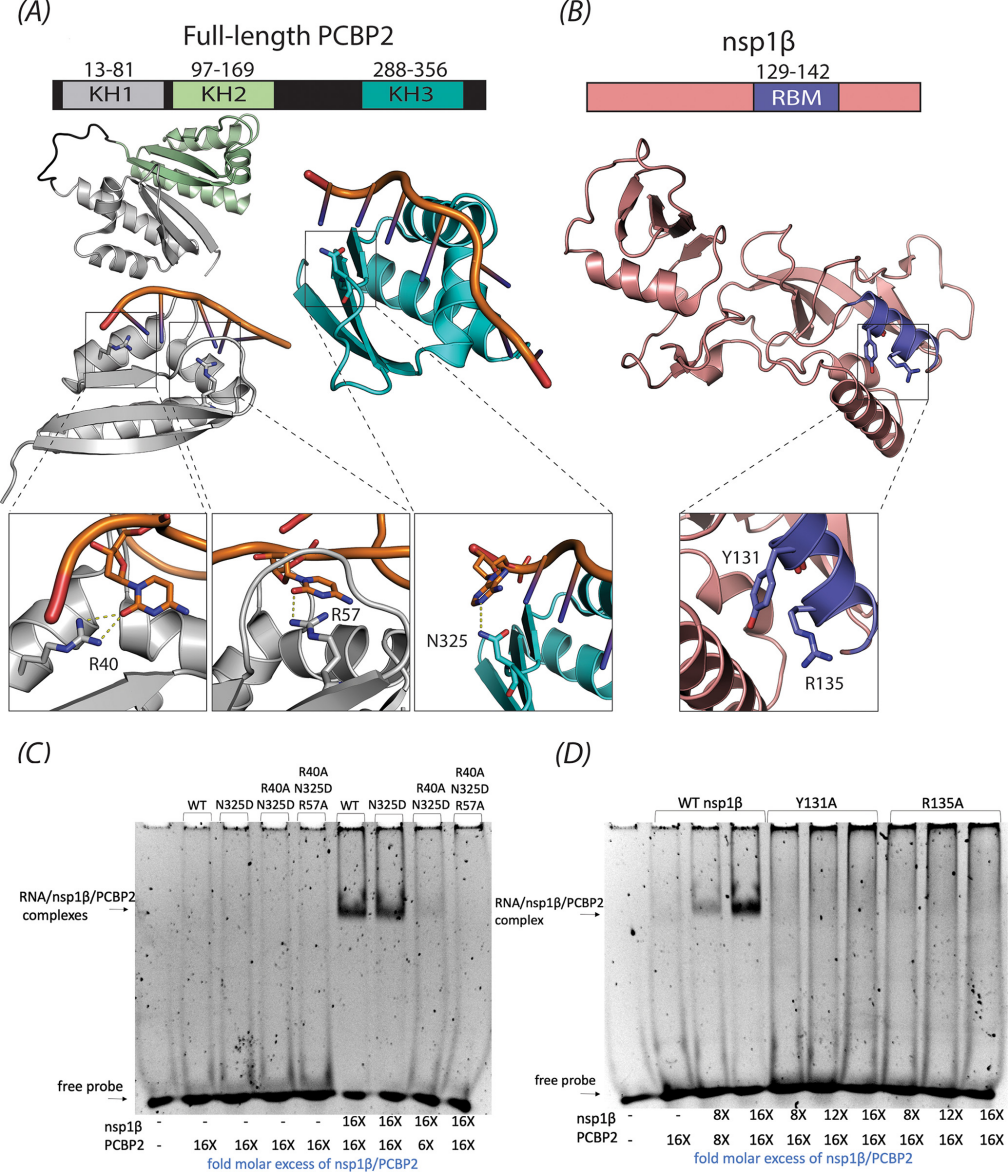
**Structure-guided mutational analysis of PCBP2 and nsp1β binding to PRRSV RNA.***A*, probing nucleic acid interaction sites of PCBP2. A schematic of full-length PCBP2 showing KH1 (*gray*), KH2 (*green*), and KH3 (*teal*) domains with accompanying three-dimensional structures (PDB entries 2P2R (26), 2JZX (24), and 2PQU (25) for DNA-bound KH1, KH1-KH2 fusion, and DNA-bound KH3, respectively). The KH1 guanidino groups of Arg^40^ and Arg^57^ appear to hydrogen-bond with the keto group of a cytosine nucleobase, whereas the side chain of Asn^325^ in KH3 is within hydrogen-bonding distance of an adenine nucleobase. Whereas the published structure has the amino group of Asn^325^ interacting with adenine, it is more likely that the carboxamide is rotated 180° to allow the carbonyl group to interact with the base instead. *B*, probing PRRSV nsp1β interactions with nucleic acid. Shown is a schematic of nsp1β from PRRSV strain XH-GD (PDB entry 3MTV (22)) with the putative RNA-binding motif (14) and residues Tyr^131^ and Arg^135^ shown in purple. Figures were generated using PyMOL (27). *C* and *D*, EMSAs performed with a 20 μm concentration of the 34-nt ssRNA (Fig. 1*B*). In *C*, WT nsp1β and two mutants (Y131A and R135A) were combined with PCBP2 and the ssRNA probe. In *D*, WT PCBP2 and three mutants (single mutant (N325D), double mutant (N325D/R40A), and triple mutant (N325D/R40A/R57A)) were combined with nsp1β and the ssRNA probe. Molar excess of each protein is listed *below* each well compared with the probe.

To gain insights into how nsp1β and PCBP2 interact with specific sequences in the PRRSV RNA genome to induce frameshifting, we first developed a robust expression and purification scheme for the proteins. Of the PRRSV isolates we tested, nsp1β from isolate SD01-08, a low-virulence European isolate from species *Betaarterivirus suid 1* (formerly type I PRRSV), was found to be the most amenable to overexpression and purification using *Escherichia coli* BL21(DE3) as an expression host (Fig. 2*A*). Whereas we tried to also express and purify the structurally characterized nsp1β from the highly pathogenic North American PRRSV isolate XH-GD (PDB code 3MTV) (21, 22) from species *Betaarterivirus suid 2* (formerly type 2 PRRSV), the attempt failed due to the protein's instability in solution, even at low concentrations. Full-length human PCBP2 was also recombinantly expressed in *E. coli* BL21(DE3) (Fig. 2*A*), and whereas a 3D structure of the complete protein has not been determined, structures of its three nucleic acid–binding domains are available (K homology domains; KH1 (23), KH2 (23, 24), and KH3 (25)) (Fig. 3*A*).

Although nsp1β and PCBP2 could be overexpressed as soluble proteins in *E. coli*, both were prone to aggregation and had low solubility during purification, which prompted us to identify optimal buffer systems for the proteins. This was determined empirically by screening 96 buffer conditions (Hampton Research) to find conditions that increase the thermal stability of the proteins. Increased SYPRO Orange (Sigma–Aldrich) fluorescence arising from protein unfolding was used to analyze protein denaturation curves as described (28). A buffer that was found to enhance the thermal stability of both proteins consisted of 1× PBS (pH 7.4), 300 mm NaCl, 100 mm KCl, and 5% glycerol. Further, because nsp1β is cysteine-rich, DTT was added to a final concentration of 2 mm to avoid cysteine oxidation. Using this optimized buffer system, we were ultimately able to isolate each protein to high purity (Fig. 2*A*), although concentrating either protein to above 1 mg/ml invariably led to aggregation. Nevertheless, they were stable and monodisperse at concentrations needed for nucleic acid interaction studies by electrophoretic mobility shift assays (EMSAs; Fig. 2, *B* and *C*). We were ultimately able to increase their stability and concentration for biophysical analyses by complex formation with viral RNA, as will be described below.

### Formation of quaternary complexes of nsp1β and PCBP2 to RNA or DNA probes

Nsp1β and human PCBP2 form a complex with RNA probes that contain the slippery sequence and C-rich motif that is found within the nsp2-coding region of the PRRSV RNA genome (29). To characterize the biochemistry of this complex in greater detail, we carried out a series of EMSAs with nucleic acid probes of systematically decreasing size to identify the shortest RNA fragment to which the proteins would stably bind, with the aim of identifying a compact protein:RNA complex amenable to preparative (milligram) scale purification. It was shown previously that a 58-nt RNA probe could be used for complex formation in a native PAGE EMSA between nsp1β, PCBP2, and an extended nucleic acid probe derivative of the PRRSV (SD01-08) genome (17). Initially, we worked with ssDNA probes (all uracil nucleotides of the frameshift site of the RNA genome changed to thymine) as DNA is more stable and cost-effective to work with. Structural studies indicate that the methyl group of thymine, which is not present on uracil, does not interact with the KH domains of PCBP2 and that it is the O2 and N3 groups of a thymine/uracil nucleoside that interact with the PCBP2 amide backbone directly (23, 25). Systematically, we were able to truncate the nucleic acid probe down to a minimum of 34 nt (Fig. 1*B*), which includes the slippery sequence, C-rich motif, and seven additional nucleotides at the 3′ end (CAGCUUU). Truncations to a size shorter than 34 nt resulted in very weak complex formation and a lack of sample monodispersity.

Using the 34-nt probe, an EMSA was initially performed with WT nsp1β, PCBP2, and the ssDNA nucleic acid probe (analogous to RNA in Fig. 1*B*). As shown in Fig. 2*B*, nsp1β does not appear to interact with the nucleic acid alone even at a 20-fold molar excess in relationship to the probe, which is consistent with previous findings (17). Interestingly, PCBP2 does interact with the ssDNA probe on its own (Fig. 2*B*), but not with ssRNA (Fig. 2*C*). This can be seen as low as an 8-fold molar excess but is highly amplified when the amount of PCBP2 is increased, as seen when a 20-fold molar excess is added in relation to nucleic acid. When both nsp1β and PCBP2 are present with the probe, a shift can be seen compared with PCBP2 bound to DNA alone, indicating the formation of a trimeric complex.

Last, we wanted to confirm the importance of the cytosine-rich motif as it pertains to complex formation. The CCCATCTCC stretch of the ssDNA probe was mutated to GAAATATGG, which is termed the 34-nt CC2 DNA (14). This probe had all cytosine nucleobases of the C-rich motif altered to guanines or adenines to see whether complex formation, as it pertains to this specific probe, could be abolished simply by disrupting this previously implicated binding site (13, 14, 17). As can be seen, even in the presence of a large molar excess of both proteins, no complex formation is detectable with the mutated probe, indicating the crucial role of cytosine or potentially CT repeats (Fig. 2*B*). These repeats have been implicated in being present in ssDNA sequences that interact with the KH domains of PCBP2 (30, 31, 32). It can also be theorized that base stacking within CT repeats may arise in the nucleic acid molecule, further predisposing it towards these nucleobase-amino acid interactions (24, 26, 31).

Results using DNA (Fig. 2*B*) suggest that PCBP2 may facilitate nsp1β binding to the nucleic acid. However, when the assay was performed with a 34-nt ssRNA probe (Fig. 2*C*), even with a large molar excess of PCBP2 (28-fold), PCBP2 did not appreciably interact with the RNA probe alone. This is consistent with previous work demonstrating that PCBP2 has a higher affinity toward ssDNA compared with ssRNA (30). Our results and those of others (17) also demonstrate that nsp1β does not interact with RNA or DNA independently. Thus, nsp1β may bind PCBP2 to enhance its affinity for RNA and thereby enable all three components to assemble into a quaternary complex that promotes frameshifting. Indeed, as we found for the ssDNA probe, when nsp1β, PCBP2, and ssRNA are combined in tandem, they form a readily detectable complex *in vitro* (Fig. 2*C*).

### Probing the protein-RNA binding interface of the PCBP2-nsp1β-RNA complex

Guided by known NMR and X-ray structures of PCBP2 KH domains (25, 26) and full-length nsp1β (22), we generated a number of site-directed mutations aimed at identifying residues of PCBP2 and nsp1β that are essential for RNA binding and PRF. For PCBP2, three variants were made within the first and third KH domains (Fig. 3*A*), including a single mutant (N325D), a double mutant (N325D/R40A) and a triple mutant (N325D/R40A/R57A). The N325D single mutation was constructed on the basis of a crystal structure of the KH3 domain bound to a short piece of C-rich, ssDNA with sequence AACCCTA (PDB entry 2P2R). As shown in Fig. 3*A*, the carboxamide side chain of Asn^325^ is within hydrogen-bonding distance (3.2 Å) of N1 of an adenine base. The N325D mutation retains the overall shape of asparagine but imparts a negative charge that we predicted would disrupt nucleic acid binding without altering the structure of the KH domain. The R40A and R57A mutations were constructed based on the crystal structure of the KH1 domain bound to C-rich ssDNA (AACCCTAACCCT) (PDB entry 2PQU). The guanidinium group of Arg^40^ forms two interactions with the keto group of a cytosine nucleobase by the formation of hydrogen bonds of 3.1 and 2.9 Å (Fig. 3*A*). This interaction suggests Arg^40^ may be a key residue for interaction(s) with the C-rich motif of the PRRSV genome. Similarly, the guanidinium group of Arg^57^ forms a 3.2-Å hydrogen bond with an additional cytosine base that we also predicted participates in binding the C-rich motif (Figs. 1*B* and 3*A*).

Using the above mutations, EMSAs were first carried out using WT nsp1β to gain insight into how the PCPB2 mutations affected complex formation (Fig. 3*C*). Compared with WT PCBP2, the N325D mutation alone did not affect complex formation. Given the significant electrostatic repulsion that was predicted to occur, this finding suggests a lesser role for KH3 in binding to the C-rich motif in the PRRSV genome. In contrast, when the double mutant R40A/N325D was assayed, a marked decrease in complex formation was observed, implicating Arg^40^ as a key player in binding the C-rich motif. The PCBP2 triple mutant (R40A/N325D/R57A), in which two mutations were made in KH1 and one in KH3 domains, abolished PCBP2's binding capabilities to the probe and subsequently complex formation.

To probe nsp1β residues that are crucial for complex formation, the proposed RNA-binding motif (RBM; Fig. 3*B*) that is highly conserved within almost all PRRSV isolates (33) was analyzed by site-directed mutagenesis. This motif with the sequence GKYLQRRLQ is comprised of several basic amino acids that have been implicated in −2/−1 PRF stimulation (13), innate immune suppression (33, 34), and nuclear poly(A) mRNA retention of host cell transcripts, which prevents cytoplasmic entry and subsequent translation of essential cellular mRNAs in PRRSV-infected cells (34). Previous studies have shown that mutations within the RBM decreased the prevalence of frameshifting products nsp2TF and nsp2N (13) and may limit the ability of PRRSV to suppress the host innate immune response (33). Indeed, it has been speculated that the nsp2TF and nsp2N frameshifting products aid in suppressing the innate immune response (15). Nsp2 has an N-terminal papain-like cysteine protease domain (PLP2 in Fig. 1*A*) that functions in viral replicase polyprotein processing but also has deubiquitinating and de-ISGylating activities that are thought to help the virus evade porcine immunity pathways (35, 36). It stands to reason then that these auxiliary functions of nsp2 are heightened with the translation of the nsp2-variant frameshifting products due to the presence of the PLP2 domain within all three proteins (15). Previous studies revealed that nsp2 and nsp2TF are both membrane-associated but are targeted to different compartments in the infected cell (14). Furthermore, nsp2N lacks a predicted transmembrane domain (Fig. 1*A*) that would tether it to a membrane. It may thus be a cytosolic protein (15), possibly acting as a deubiquitinase that corrupts the host ubiquitin system to suppress innate immune responses (37).

To investigate the biochemistry of the nsp1β RBM motif, two point mutations (Y131A and R135A) were independently introduced. In contrast to WT nsp1β, the nsp1β-Y131A and nsp1β-R135A mutations abolished complex formation with RNA when combined with WT PCBP2 (Fig. 3*D*), implicating these residues in the formation of the quaternary complex. Given these results, and previous mutational analyses of the region (17), nsp1β RBM appears to promote RNA binding but only in the presence of PCBP2. Previous yeast two-hybrid experiments have found the two proteins to interact (18), suggesting that their binding may induce conformational changes in one or both proteins that favor RNA binding and stimulation of −1/−2 PRF, because neither appreciably binds RNA on its own (Fig. 2*C*).

### Mutations in the nsp1β RBM motif also prevent −1/−2 PRF product formation

To gain deeper insight into the role of the nsp1β RBM motif in −1/−2 PRF stimulation, we systematically analyzed its role in −1/−2 PRF by mutating each residue to an alanine (Gly^129^–Arg^142^ of the SD01-08 PRRSV strain). The nsp1β expression plasmids were co-transfected with a plasmid expressing SD01-08 nsp2 into RK13 cells that were infected with a recombinant vaccinia virus expressing T7 RNA polymerase. Subsequently, nsp2, nsp2TF, and nsp2N were metabolically labeled, immunoprecipitated, and separated by SDS-PAGE. The expression of the three nsp2 variants was quantified in each condition and compared with the situation in which nsp2 was co-expressed with WT nsp1β. Nsp1β mutant expression was confirmed by immunoprecipitation using an antiserum recognizing the N-terminal 3xFLAG tag.

**Figure 4:**
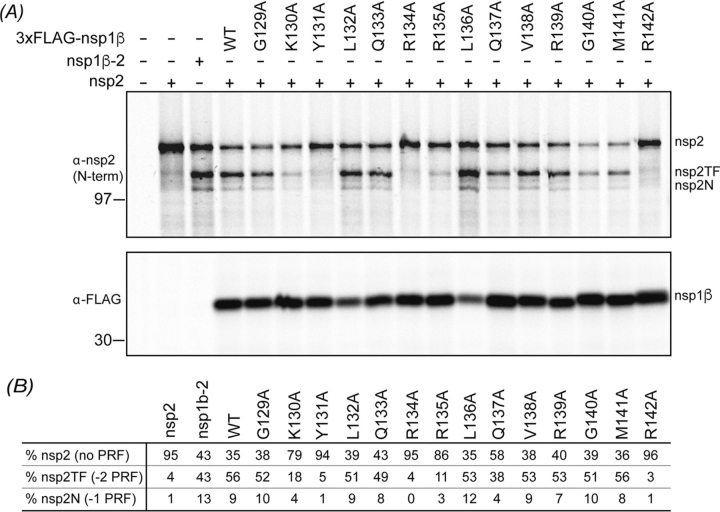
**Analysis of *trans*-activating frameshift stimulation by nsp1β mutants.** Plasmids expressing SD01-08 PRRSV WT or mutant nsp1β were co-transfected with a plasmid expressing nsp2 in RK13 cells infected with a recombinant vaccinia virus expressing T7 RNA polymerase. As controls, single expression of nsp2 or nsp1β, expression of a self-cleaving nsp1β-2 polyprotein, and a nontransfected sample were included. *A*, following ^35^S metabolic labeling, proteins were immunoprecipitated with mAb58-46 (nsp2, nsp2TF, and nsp2N) or mAb-FLAG (nsp1β) and analyzed by SDS-PAGE and autoradiography. Size markers and the positions of bands for nsp2, nsp2TF, nsp2N, and nsp1β are indicated *beside* each *panel*. *B*, band intensities were quantified by phosphor imaging and corrected for amino acid content and Met/Cys incorporation efficiency, after which the nsp2, nsp2TF, and nsp2N levels were used to calculate ribosomal frameshifting efficiencies.

As before, PRRSV −2/−1 PRF was found to be highly efficient in this expression system. As seen in Fig. 4*A*, when only nsp2 was expressed, the nonframeshifted, full-length nsp2 constituted ∼95% of the protein products immunoprecipitated with an antibody recognizing the N-terminal domain of nsp2. In the control expressing a self-cleaving nsp1β-nsp2 polyprotein from a single plasmid, there were equal amounts of nsp2 and nsp2TF produced and ∼13% nsp2N. With WT nsp1β and nsp2 expressed from separate plasmids, ∼56% was the −2 PRF product nsp2TF, and ∼9% was the −1 PRF product nsp2N. For three nsp1β mutants (Y131A, R134A, and R142A), the level of −1/−2 PRF was as low as in the control expressing nsp2 only (Fig. 4*A*), highlighting the importance of these residues in PRF. The Y131A and R134A mutations in this nsp1β variant (from PRRSV isolate SD01-08) correspond to the Y125A and R128A mutations in nsp1β from PRRSV isolate SD95-21. For this previously used isolate, mutations Y125A and R128A were also found to almost completely abolish PRF stimulation (13, 16). For SD01-08 mutants K130A and R135A there was a significant reduction in both −2 (∼70–80% reduction) and −1 PRF (∼55–65% reduction). For Q137A, the reduction observed was less, with ∼30% reduction in −2 PRF and a ∼55% reduction for −1 PRF. The nsp1β expression level likely affects frameshifting efficiency, as previously described for viral protein 2A in the encephalomyocarditis virus. This protein acts as a PRF transactivator by binding to a genomic stem-loop structure, resulting in variable frameshift stimulation, from 0% at the start of infection to 70% late in infection as the concentration of 2A protein in the cells increased (29). Poor nsp1β expression could, therefore, result in reduced frameshift stimulation. However, the decrease in frameshifting that we observed for mutants K130A, Y131A, R134A, R135A, Q137A, and R142A could not be explained by insufficient expression of the nsp1β mutants in those samples because protein levels of these nsp1β mutants were comparable with WT nsp1β. The L132A and L136A mutants were expressed to lower levels than WT, but for these mutants, frameshift efficiencies were as high as with WT nsp1β, so the amount of protein expressed was still sufficient for efficient frameshift stimulation (Fig. 4*A*).

### Mutations in the nsp1β RBM motif affect innate immune suppression

Nsp1β and both −2/−1 PRF products, nsp2TF and nsp2N, have been implicated in suppressing host innate immune responses (15, 35, 38, 39, 40). Nsp1β may influence innate immune suppression in multiple ways. The protein was proposed to modulate the host immune response directly but may also influence it indirectly through the −2/−1 PRF mechanism that directs nsp2TF and nsp2N expression. Specifically, the nsp1β RBM motif has been associated with both innate immune suppression and −2/−1 PRF stimulation (13, 34). When recombinant viruses with nsp1β RBM mutations are studied, it is not possible to establish whether phenotypic changes are caused by a reduced innate immune evasion capacity of nsp1β, altered −2/−1 PRF and nsp2TF/nsp2N expression levels, or a combination of the two.

**Figure 5:**
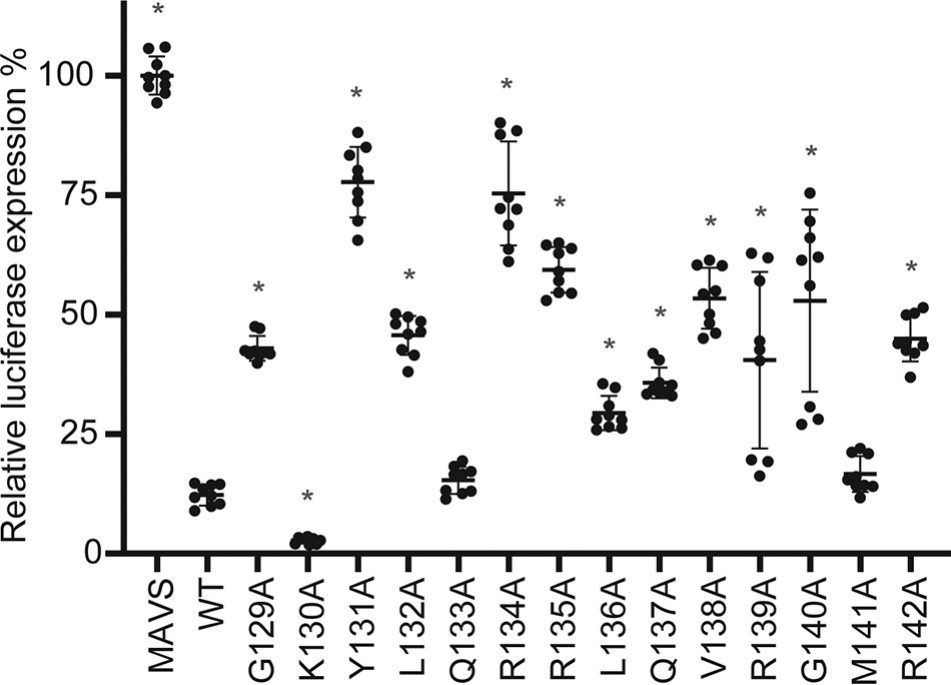
**Analysis of innate immune suppression by expression of PRRSV nsp1β mutants.** HEK293T cells were co-transfected with plasmids expressing innate immune response inducer MAVS, a firefly luciferase reporter gene under control of the IFN-β promoter, *Renilla* luciferase, and WT or mutant PRRSV nsp1β. Cells were lysed 18 h post-transfection, and the *Renilla and* firefly luciferase activities were measured. Firefly luciferase activity was normalized to *Renilla* activity in the same well. Three independent biological replicates with three technical replicates each are shown with S.D. values. Significance (*p* < 0.001) was assessed using an unpaired two-tailed Student's *t* test and is indicated with an *asterisk*.

To study the different roles of nsp1β independently, it is important to uncouple its innate immune suppression function(s) from its PRF-stimulatory activity. We have, therefore, also tested the impact of RBM mutations on nsp1β's ability to antagonize activation of the IFN-β response by using a Dual-Luciferase reporter assay. Cells were co-transfected with plasmids expressing mitochondrial antiviral signaling protein (MAVS), which stimulates the pathway leading to IFN-β production, and either WT or mutant nsp1β. The inhibitory effect of nsp1β mutants on IFN-β promoter activation was measured via co-transfection of a firefly luciferase reporter gene construct under control of the IFN-β promoter. To correct for transfection efficiency variability, a plasmid encoding *Renilla* luciferase was co-transfected to provide an internal standard. At 18 h post-transfection, *Renilla* and firefly luciferase activities were measured. Activation of the IFN-β promoter induced by MAVS expression only was set to 100%. As seen in Fig. 5, three mutants, K130A, Q133A, and M141A, suppressed activation of the IFN-β promoter to an extent that was comparable with the suppression by WT nsp1β. Expression of mutants Y131A, R134A, R135A, V138A, and G140A still allowed >50% of luciferase expression, indicating a strongly reduced ability to suppress IFN-β promoter activation. Mutants Y131A and R134A seem to be severely affected in both PRF stimulation (Fig. 4) and innate immune suppression (Fig. 5). Interestingly, mutant R142A, which was incapable of PRF stimulation, reduced luciferase expression by only 50%. Mutant K130A appeared to antagonize IFN-β activation even better than the WT protein, whereas its reduction in −2 PRF stimulation is ∼70%. For most other mutants, some reduction in innate immune suppression capability was observed, whereas PRF stimulation did not appear to be affected. Consequently, for future studies with recombinant viruses carrying nsp1β mutations that reduce PRF stimulation, it may be advisable to use mutant K130A rather than Y131A, R134A, or R135A, because the latter three mutations may also affect the protein's ability to counter innate immune responses in infected cells. The nsp1β mutants most able to suppress innate immune responses also suffer from strongly reduced −2/−1 PRF stimulation capability, which will complicate the assessment of the direct role of nsp1β in innate immune suppression during viral infection.

### Structural insights into the −1/−2 PRF-stimulatory complex

Having identified the minimal viral RNA sequence that forms a complex with nsp1β and PCBP2, we developed an approach to purify the protein:nucleic acid complex to assess its stoichiometry and structural biology. Assuming a 1:1:1 stoichiometry, nsp1β and PCBP2 were initially mixed in a 1:1 molar ratio at concentrations <1 mg/ml with a slight excess of nucleic acid (1.1-fold molar excess) to generate the trimeric complex. After a 3-h incubation period at 4 °C, the mixture was concentrated for loading on a gel filtration column. A fair amount of precipitation arose during this step, some of which may have been PCBP2 and nsp1β molecules that had not bound nucleic acid, as we found the proteins to be unstable in the absence of nucleic acid. Regardless, the resulting protein:RNA complex could be concentrated to 10 mg/ml at this stage, which was already an order of magnitude higher than the maximum concentrations of 1 mg/ml that could be achieved for nsp1β and PCBP2 on their own.

**Figure 6:**
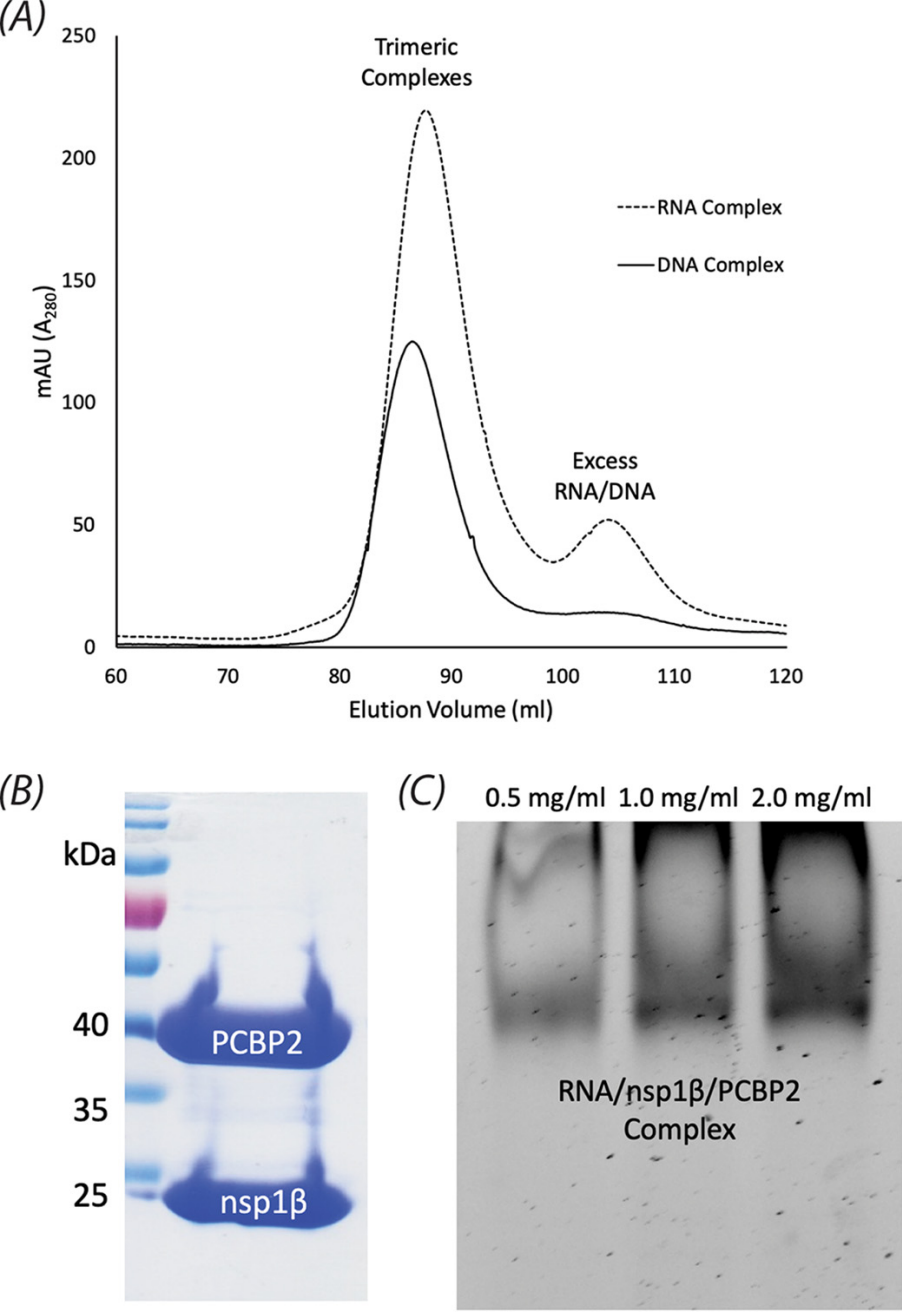
**Purification of nsp1β:PCBP2:nucleic acid complexes for biophysical analysis.** Purified PRRSV nsp1β and PCBP2 were mixed together in equimolar concentrations with a 1.1-fold molar excess of the 34-nt ssRNA or ssDNA oligonucleotides that were identical or analogous, respectively, to the PRF signal in the nsp2-coding region of PRRSV SD01-08, as portrayed in Fig. 1*B*. Both quaternary complexes were found to be stable and could be concentrated to ∼20 mg/ml prior to purification by size exclusion chromatography. *A*, elution trace of the protein:RNA complex from a Superdex200 gel filtration column monitored by UV light at 280 nm. *B*, Coomassie Blue–stained SDS-PAGE of the purified complexes bound to RNA/DNA, revealing the presence of both proteins. *C*, nondenaturing 8% TBE polyacrylamide gel of the purified complex shown in *C* stained with SYBR Gold, revealing the presence of the 34-nt RNA.

The supernatant of the concentrated sample was separated from the precipitate and subsequently purified by size exclusion chromatography (Fig. 6*A*). SDS-PAGE and native PAGE were carried out on the purified samples to assess the composition of each complex, which revealed both proteins to be present (Fig. 6*B*), as well as the nucleic acid probe (Fig. 6*C*). Remarkably, the final purified complex could be concentrated to >20 mg/ml. The complex was found to be stable for at least 10 days at 4 °C.

### The frameshift stimulatory complex exists in a 1:1:1 stoichiometry

To gain insight into the stoichiometry of the frameshifting complex, we characterized the nsp1β:PCBP2:ssRNA triple complex by the sedimentation velocity method using an analytical ultracentrifuge. The solvent and hydrodynamic parameters used during data analysis can be found in Table S3. We first measured a series of concentrations from 8 to 64 μm of the 34-nt ssRNA probe alone (Fig. S1 and Table S4). Two populations of species were apparent with roughly 80% of the material in species 1 and 20% in species 2. This ratio remained constant over the concentration range that was investigated (Fig. S1C). The experimental sedimentation constant *s_e_* and hydrodynamic radius *R_h_* of species 1 are nearly independent of concentration. Regarding species 2, *s_e_* decreases very slightly, and *R_h_* decreases moderately with increasing concentration (Fig. S1, A and B). Concentration independence or a decrease of *s_e_* and *R_h_* is a sign that the kinetics of RNA chain exchange between the two species was very slow in relation to the time course of the experiment (24 h). Both species thus effectively acted like independent molecules. Self-interaction between macromolecules with a faster kinetics would manifest as an increase of *s_e_* and *R_h_* with increasing loading concentration.

If the partial specific volume $\bar{v}$ is known, the molecular mass *M* can be calculated from the extrapolated experimental sedimentation $s_{e}^{0}$ and experimental diffusion constant $D_{e}^{0}$ (the latter is represented here as hydrodynamic radius, $R_{h}^{0}$) using the Svedberg equation. The extrapolation to infinite dilution is done to account for buffer effects. For nucleic acids, however, $\bar{v}$ is a function of ionic strength and is expected to be in the range of 0.50–0.65 cm^3^/g. Using a value of 0.628 cm^3^/g, we obtain a mass of 10.7 kDa for species 1, which corresponds to monomeric ssRNA. Dimeric or higher-order RNA could be ruled out as this would require a $\bar{v}$ outside of the expected range. For species 2, this $\bar{v}$ value yielded a mass of 79.3 kDa and would correspond to an assembly of 7–8 RNA strands (Table S4). *c*(*s, f_r_*) distributions, *c*(*s, M*) distributions, and direct fitting of *s_e_* and *D_e_* using the Lamm equation (species analysis) are provided in Fig. S2 (A–D) together with the fit to the data and the residuals.

For the second step, we prepared three concentrations of 1.0, 2.0, and 4.0 mg/ml of the SEC-purified trimeric complex (Fig. 6*A*) and conducted sedimentation velocity experiments at 30,000 rpm. We also repeated the experiments at 42,000 rpm, using the previously used samples and cells. Comparing the total signal *versus* loading concentration of both runs revealed a significant loss of signal in the second run (Fig. S3 (A, D, G, and J)). The increasing concentration of material at the bottom of the cell had led to irreversible aggregation and removal of material from the solution during the first run. We determined that the loading concentrations during the second run had reduced to 0.44, 0.75, and 1.09 mg/ml, thus expanding the investigated concentration range. Fig. S6 (A, B, E, F, I, and J) shows the calculated two-dimensional *c*(*s, f_r_*) distributions obtained from the data with the sedimentation constant *s* on the *x* axis and the diffusion constant expressed as frictional ratio *f_r_* on the *y* axis. A zoomed part of the plot was converted to mass and is represented as a two-dimensional *c*(*s*,*M*) distributions in the same figure. The one-dimensional distributions *c*(*s*,*), *c*(*s*), and fit to the data together with residuals are shown as well. An overlay of the one-dimensional distributions of all concentrations is shown in Fig. S3. The *c*(*s*,*) distributions (Fig. S3 (B, E, H, and K)) were obtained by integrating the two-dimensional distributions along the *f_r_* direction. Traditional *c*(*s*) distributions are also shown (Fig. S3, C, F, I, and L); however, these suffer from the incorrect assumption of an identical *f_r_* value for all species. Both absorbance and interference optics of the XL-I instrument were used, because the former is particularly sensitive to the nucleic acid due to its high extinction coefficient, whereas the interference optics is equally sensitive to all components. The *c*(*s*,*) distributions obtained from the interference data in Fig. S3 (E and K) revealed three major, distinct populations of particles, especially in the data recorded during the first run. The absorbance optics could not resolve individual populations (Fig. S3, B and H); however, comparing the one-dimensional distributions obtained from the absorbance optics with those obtained from the interference optics indicates that they cover the same *s* range. Thus, all three populations contained nucleic acid. Notably, the amount of nucleic acid decreases with increasing *s* values. Free RNA would show up at ∼1.2 S. No such population was present, confirming that all RNA was bound to protein in the three populations of particles we observed.

**Figure 7:**
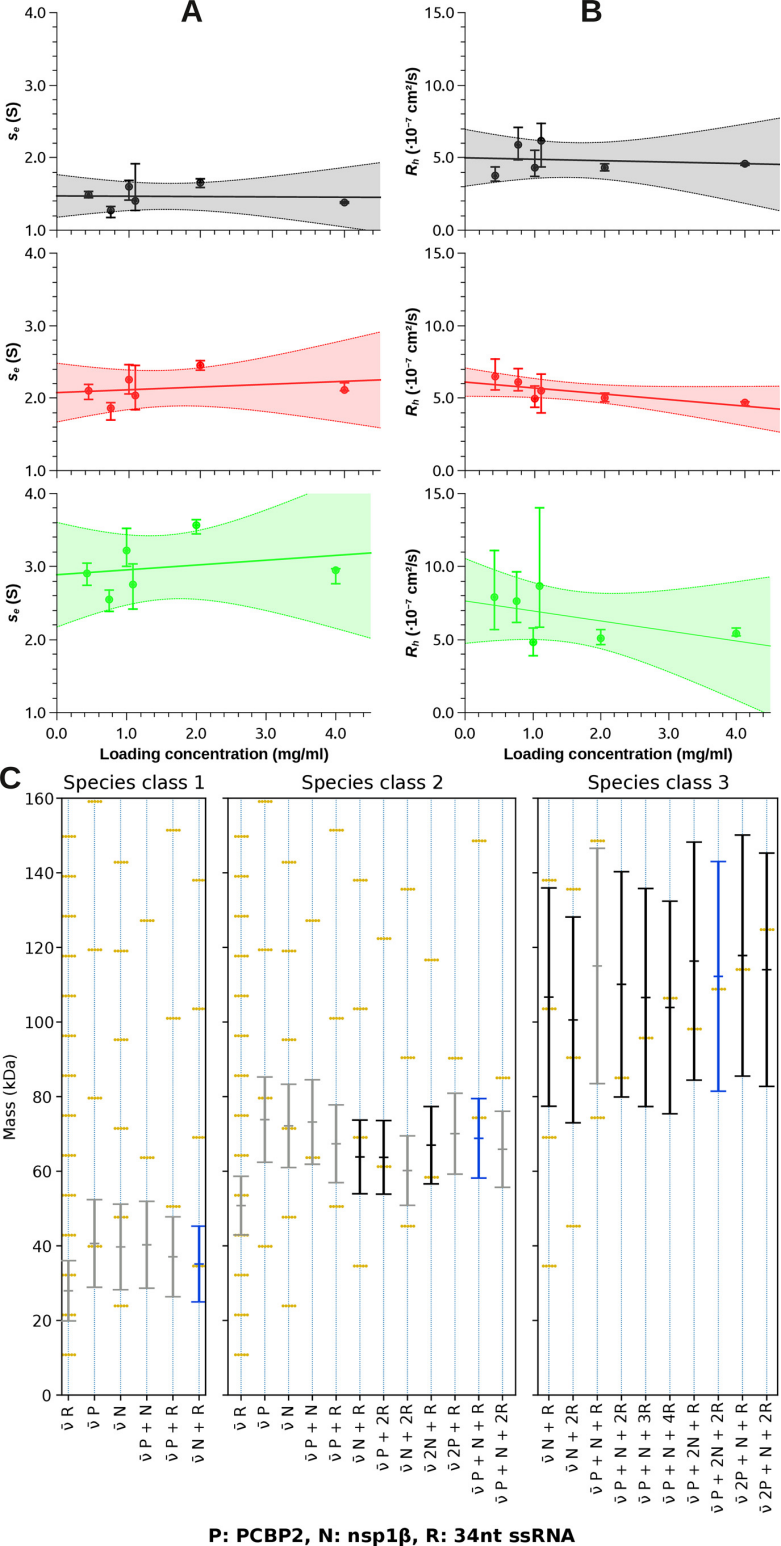
**Analytical ultracentrifugation of the trimeric complex shows a 1:1:1 stoichiometry.***A* and *B*, results of the species analysis obtained by direct fitting the sedimentation coefficient *s* and diffusion coefficient *D* of each observed particle population to the Lamm equation (41) at each loading concentration. Only species classes 1–3 are shown. Shown are experimental sedimentation coefficient *s_e_* (*A*) and *D_e_* (*B*) converted to hydrodynamic radius *R_h_ versus* loading concentration of species classes 1 (*black*), 2 (*red*), and 3 (*green*). *Vertical error bars*, 95% confidence intervals of the fitted parameter (*s_e_* or *R_h_*). The values were then extrapolated to zero concentration using an unweighted linear fit (*continuous line*), yielding $s_{e}^{0}$ and $R_{h}^{0}$. The *shaded area* shows the 95% confidence interval of the extrapolation. *C*, conversion of $s_{e}^{0}$ and $D_{e}^{0}$ to mass. The conversion relies on the partial specific volume $\bar{v}$, which depends on the ratio of the components of the complex that is shown at the *bottom* of the plot. Multiples of the same ratio have the same $\bar{v}$; the corresponding mass ladders are shown as *golden rungs*. Due to the ambiguity of $\bar{v}$, multiple solutions are possible. From the absorbance optics, we know that species 1–3 must contain RNA. All solutions without RNA and solutions that do not intersect with a mass ladder rung can be excluded (*gray bars*). We have marked the solution for each species class we deem the most likely with a *blue bar*. *Black bars* show alternate possible solutions. The *error bars* represent 95% confidence intervals, which are based on the experimental uncertainties of $s_{e}^{0}$, $D_{e}^{0}$, and the solvent density and viscosity.

To analyze the data in more detail, we directly fitted *s_e_* and *D_e_* of the observed populations (Fig. 7 (*A* and *B*) and Fig. S4) using the Lamm equation. As a courtesy to the reader, a copy of Fig. 7 is included in the supporting information (Fig. S5). Depending on their sedimentation coefficients and hydrodynamic radii, we sorted the observed species into classes (Fig. S4, A and B). Three main species classes were present at all concentrations, with species class 1 and 2 each contributing ∼30–40% and species class 3 contributing ∼10–20% to the signal (Fig. S4C). At loading concentrations from 0.754 mg/ml and higher, we observed additional species classes with larger *s* values. Species class 4 contributed ∼5–10% to the signal. The remaining signal (5–15%) was shared by species classes 5–7 with very large sedimentation constants and very small hydrodynamic radii (equivalent to very large diffusion coefficients). These represent either extreme shapes or nonideality.

Surprisingly, *s* and *R_h_* of species class 1, 2, and 3 remained constant or decreased with increasing loading concentration (Fig. 7, *A* and *B*), indicating stable particles with a very slow exchange of components with the other species (relative to the time course of the experiment). We could therefore extrapolate the values to infinite dilution and determine $s_{e}^{0}$, $D_{e}^{0}$, and $R_{h}^{0}$ (Fig. 7 *(A* and *B*) and Table S5, which shows the values converted to standard conditions).

As mentioned earlier, $s_{e}^{0}$ and $D_{e}^{0}$ can be converted to mass if the $\bar{v}$ is known. Nsp1β and PCBP2 were produced by bacterial expression and therefore not glycosylated, and their $\bar{v}$ could be accurately predicted from the amino acid sequence (42). As described above, we measured the $\bar{v}$ of the 34-nt ssRNA probe alone in the same buffer environment in which we produced the complex. We thus knew the $\bar{v}$ of every component and could calculate the resulting $\bar{v}$ and mass of each species class for every conceivable composition. Fig. 7*C* shows a collection of conceivable masses for species classes 1–3.

Particles in species class 1 were roughly half the molecular weight of those in species class 2. As the absorbance optics tell us, there must be nucleic acid and protein present within all three species. As seen in Fig. 7*C* (species class 1), the only possible composition involving nucleic acid could be a monomer of nsp1β bound to a monomer of RNA (total mass of ∼35 kDa). This was unexpected because neither protein alone appears to bind RNA in EMSAs (Fig. 2*C*). Nevertheless, there is precedence for this behavior due to the presence of the RBM of nsp1β, and it is possible that this interaction is not be detectable by EMSA. This suggests that nsp1β directly interacts with nucleic acid in the final tricomponent system.

Regarding species class 2, more than a single stoichiometry of the components would match the experimentally determined mass (*e.g.* nsp1β:RNA (2:2) or PCBP2:RNA (1:2)). However, the eluate from the size exclusion column contains both nsp1β and PCBP2 (Fig. 6*B*), and PCBP2 on its own is not competent to bind the RNA probe. We therefore deem the nsp1β:PCBP2:RNA 1:1:1 stoichiometry (triple complex) that also falls within the 95% confidence interval the most likely solution.

The third species class was present in lower abundance (10–20%), which resulted in a noisier signal and, therefore, a larger uncertainty of the mass, which falls in the range from 75 to 150 kDa, depending on $\bar{v}$ and the size of the confidence interval (Fig. 7*C*). Still, the mass is outside of the confidence interval of a supercomplex composed of two copies of the tricomponent complex in a 1:1:1 stoichiometry (∼150 kDa). We theorize that two independent monomers of nsp1β have bound two individual monomers of the RNA probe, and they are held together by one copy of PCBP2 to give a mass of ∼110 kDa. It is possible that PCBP2 is tethering together this supercomplex by utilizing both the KH1 and KH3 domains independently; however, given its low abundance and stoichiometry, this complex may not be biologically relevant.

The above results suggest that nsp1β from PRRSV isolate SD01-08 from species *Betaarterivirus suid 1* exists as a monomer both in solution and in the PRF-stimulatory complex; however, nsp1β from PRRSV isolate XH-GD from species *Betaarterivirus suid 2* is reported as a dimer according to its X-ray structure (PDB entry 3MTV (22)). The two proteins share an amino acid sequence identity of 40% (EMBOSS Needle (43)), and of the 32 amino acids identified by PISA (44) in the proposed homodimer interface of XH-GD nsp1β, 12 (38%) are conserved with nsp1β from SD01-08. Given our observations, the multimeric state of nsp1β may differ between the two isolates; however, it is also possible that nsp1β from PRRSV isolate XH-GD dimerizes at concentrations required for its crystallization.

### Small-angle X-ray scattering (SAXS) supports a 1:1:1 binding stoichiometry for the nsp1β:PCBP2:ssRNA complex

To further understand the structure of the nsp1β:PCBP2:RNA complex, a three-dimensional molecular envelope of the complex was determined experimentally by SAXS, which provides insights into the low-resolution structural information of biomolecules and their complexes under physiological buffer conditions. We used a HPLC-SAXS setup to collect scattering data for the complex of nsp1β:PCBP2:ssRNA. The X-ray scattering trace and UV traces of the 10 mg/ml sample eluting from the 4.5-ml Shodex KW40 column are shown in Fig. S7.

**Figure 8:**
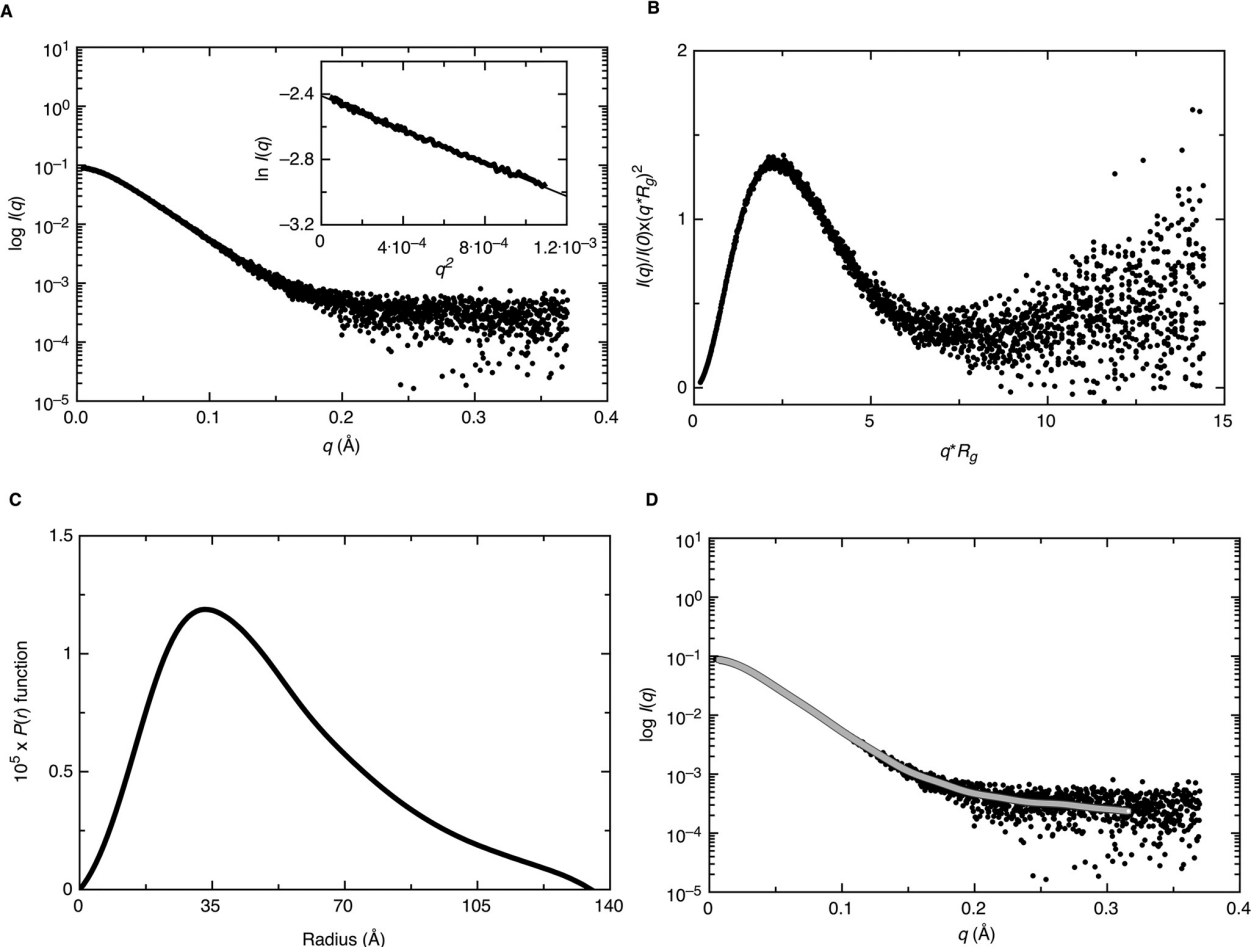
**Characterization of the PRRSV nsp1β:PCBP2:nucleic acid complexes using SAXS.***A*, plot of scattering intensity *versus* scattering angle presenting the merged data for nsp1β:PCBP2:ssRNA. The *inset* to this plot is the Guinier analysis, which confirms the homogeneity of the complex. *B*, dimensionless Kratky plot for the nsp1β:PCBP2:ssRNA, demonstrating its extended structure in solution. *C*, a pair-distance distribution (*P*(*r*)) plot for nsp1β:PCBP2:ssRNA complex allowing the determination of *R_g_* and *D*_max_. *D*, alignment between experimentally collected SAXS data (*dark circles*) and calculated data from representative low-resolution models (*solid lines*).

Consistent with the sedimentation velocity results, the UV trace reveals some heterogeneity of the sample. However, the main peak of the X-ray trace is symmetric and homogeneous and originates predominantly from a single species. The buffer-subtracted and merged SEC-SAXS data taken from frames at the peak center are presented in Fig. 8*A*. Next, we performed the Guinier analysis of merged data to ensure the purity of the complex and to determine the *R_g_* (radius of gyration) from the SAXS data belonging to the low-*q* region (45). The *inset* to Fig. 8*A* represents the Guinier plot for the complex with a linear region at low-*q* values, indicating that the complex is monodispersed. The Guinier analysis for the complex also provided an *R_g_* value of 3.900 ± 0.011 Å (Table 1). Next, we processed the SAXS scattering data from Fig. 8*A* to perform Kratky analysis to investigate the folding state of biomolecules (46, 47). The globular-shaped biomolecules typically display a well-defined maximum value of 1.1 at *q*·*R_g_* = 1.73 (48). As presented in Fig. 8*B*, the Kratky analysis for the complex under investigation suggests that it is well-folded and has extended conformation in solution. Now that we had confirmed the homogeneity and folded state of this complex, we converted the SAXS raw data into the real space electron pair-distance distribution function (*P*(*r*)), as presented in Fig. 8*C* using the program GNOM (49). We also obtained *R_g_* and *D*_max_ (maximum particle dimension) values of 4.002 ± 0.008 and 13.5 nm, respectively (Table 1). The *R_g_* values from Guinier analysis, which only takes in account the low-*q* region, and from *P*(*r*) analysis, which utilizes a wider range of SAXS data, agree with each other as well as the Kratky analysis suggests that the complex is folded, implying that we had suitable data for low-resolution structure analysis.

**Table 1:** Biophysical parameters of the PRRSV nsp1β:PCBP2:ssRNA 1:1:1 complex Uncertainties are given in parentheses as 95% confidence intervals.

Parameter	Experimental value	Experimental method	Dammin models
Sedimentation coefficient $s_{20,w}^{0}$ (S)	2.84 (2.28–3.41)	SV	4.22 ± 0.02^a^
Hydrodynamic radius $R_{h20{}^{\circ}C,w}^{0}$ (nm)	6.11 (5.17–7.46)	SV	4.40 ± 0.02^a^
Molecular mass *M*^b^ (kDa)	68.8 (58.2–79.5)	SV	
Molecular mass *M* (kDa)	86 ± 6	SAXS, Primus	
Formula mass (Da)	*74,224.49*		
Extrapolated scattering intensity at 0 angle *I*(*0*)	(0.089 ± 1.60)·10^4^	SAXS, Guinier	
Radius of gyration *R_g_* (nm)	3.900 ± 0.011	SAXS, Guinier	
Extrapolated scattering intensity at 0 angle *I*(*0*)	(0.089 ± 1.35)·10^4^	SAXS, *P(r)*	
Radius of gyration *R_g_* (nm)	4.002 ± 0.008	SAXS, *P(r)*	4.044 ± 0.003
Longest dimension *D*_max_ (nm)	13.5	SAXS, *P(r)*	13.98 ± 0.02
Volume *V* (nm^3^)		SAXS	165 ± 1
χ^2^ of fit		SAXS	∼1.3
Normalized spatial discrepancy (NSD)		SAXS	0.60 ± 0.01


a Calculated in Hydropro.

b Using $\bar{v}$ = 0.7166 cm³/g.

We utilized the DAMMIN program (50) to calculate 20 models for the nsp1β:PCBP2:nucleic acid complex, as described earlier (51, 52). The χ^2^ values in each case were ∼1.3, representing a good agreement between the experimentally collected and low-resolution structure–derived data (Table 1), as presented in Fig. 8*D*. Finally, we used the DAMAVER package (53) to rotate and align all 20 low-resolution structures and to obtain an averaged filtered structure of the complex. The normalized spatial discrepancy (NSD) parameter in DAMAVER describes the goodness of the superimposition of individual models. For the nsp1β:PCBP2:nucleic acid complex, we obtained an NSD value of 0.60 ± 0.01, indicating that all 20 low-resolution structures are very similar to each other. DAMMIN reported an *R_g_* of 4.044 ± 0.003 nm and a *D*_max_ of 13.98 ± 0.02 nm for its models, which agrees with the numbers obtained from GNOM analysis (Table 1). We also performed HYDROPRO (54) calculations of all 20 low-resolution structures using the partial specific volume of the triple complex to calculate the sedimentation coefficients and hydrodynamic radii of the models for comparison with the experimental data (Table 1) (55).

**Figure 9:**
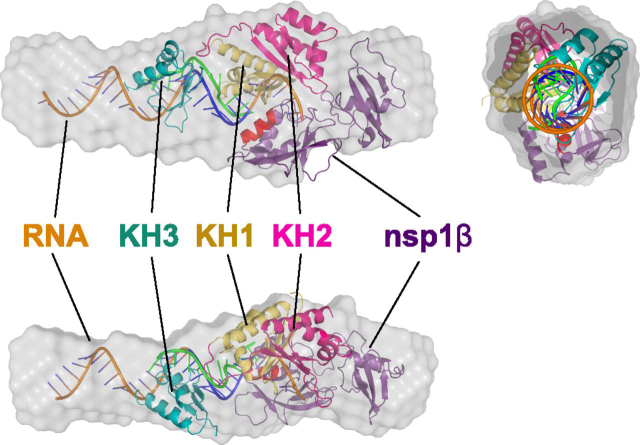
**Low-resolution SAXS structure of the trimeric complex is consistent with a 1:1:1 stoichiometry.** The SAXS envelope is shown in three different orientations with the calculated density filled in with existing protein structures and modeled nucleic acid. In *purple* is a monomer of nsp1β from PRRSV strain XH-GD (PDB entry 3MTV (22)) with the theorized RBM helix depicted in *red*. In *gold* and *fuchsia* are the KH1 and KH2 domains of PCBP2, respectively (PDB entry 2JZX (24)). In *green* is shown a short, C-rich RNA motif that was co-crystalized with KH1 alone (PDB entry 2PY9 (23)), which is shown as a frame of reference for how our idealized RNA probe (*orange*) may fit. In *teal* is the KH3 domain of PCBP2 (PDB entry 2P2R (25)). The RNA molecule is shown in *orange* with the C-rich motif shown in *blue*. All fitting was carried out manually using PyMOL (27).

Fig. 9 presents the elongated averaged filtered structures for the nsp1β:PCBP2:nucleic acid complex into which existing 3D structures of nsp1β (PDB entry 3MTV (22)), the KH1-KH2 region (PDB entry 2JZX) (24), and KH3 (PDB entry 2P2R) of PCBP2, respectively, were manually fitted along with a helical model of the 34-nt ssRNA probe (generated by w3DNA 2.0 (56)) using the known biochemical interactions that occur between the RNA and proteins as a guide. Whereas rigid-body modeling of the components into the SAXS envelope was attempted using CORAL (57), the results did not converge on a consistent solution that fit within the envelope nor satisfy known biochemical interactions between nsp1b, PCBP2, and the viral RNA. The poor fitting was likely due to 1) 35% (126 amino acids (9 at the N terminus and a 117-amino acid linker)) of PCBP2 being disordered and absent from known X-ray structures of the protein and 2) being limited to using an idealized helix for the RNA, which is unlikely to be the conformation adopted in the natural complex.

In lieu of computation-based fitting, we chose to build a rudimentary prediction of the complex by manually fitting components into the SAXS envelope. Given our sedimentation velocity data above, the crystal structure of nsp1β (from PRRSV strain XH-GD (22)) was fitted as a monomer. The NMR solution structure of the PCBP2 KH1-KH2 fusion was also fitted along with the X-ray structure of the third KH domain (PDB entry 2P2R) (25). It was previously found that KH1 and KH3 participate in nucleic acid binding, whereas KH2 does not (17). Given this constraint, the nucleic acid–binding regions of KH1 and KH3 were oriented toward the C-rich region of the ssRNA within the modeled complex, as was the RBM helix of nsp1β. The relative position and orientation of KH1, KH2, and KH3 of PCBP2 to each other and to the ssRNA probe were modeled based on the X-ray structure of KH1 bound to RNA (PDB entry 2PY9 (23)). All fitting was carried out in PyMOL (27).

As shown in Fig. 9, the SAXS envelope is tubular with a distinct bulge at one end. An idealized helical model of the 34-nt ssRNA fits within the tubular portion of the envelop, whereas the bulge is large enough to account for one molecule each of nsp1β and PCBP2, which is in agreement with our AUC findings of a 1:1:1 stoichiometry. It should be noted that in the X-ray structure of KH1 bound to RNA, the RNA does not adopt a perfectly helical conformation and is instead more linear (23). Thus, it is likely that the RNA within the nsp1β:PCBP2:RNA complex is also not perfectly helical throughout. Nevertheless, the resolution of our SAXS data are insufficient to gain insight into the true conformation of the ssRNA probe and thus was left in an ideal helical conformation. Further, 3D structural information is not available for residues 170–287 of PCBP2 that span between the KH2 and KH3 domains. This 117-amino acid region, plus the 9 amino acids missing from the N terminus accounts for ∼35% of the total PCBP2 structure and is thought to be highly disordered (24, 25, 26). In keeping with this, the SAXS envelope does not appear to account for this mass, which we assume is due to the high degree of disorder in this region of PCBP2.

Finally, the C-rich motif, and presumed binding site of the nsp1β:PCBP2 complex, is positioned near the 3′-end of the 34-nt ssRNA probe. This position is consistent with the location of the bulge that appears in the SAXS envelope and thus where we believe nsp1β and PCPB2 bind to the RNA (Fig. 1*B*). The co-localization of nsp1β and PCPB2 in the model is consistent with our hypothesis that both nsp1β and PCBP2 interact directly with the viral RNA genome at the C-rich motif.

### Conclusion

Together, our results provide new structural and functional insights into the unique PRF mechanism that is employed by arteriviruses, in which a viral and a host protein cooperate with a specific signal in the viral RNA genome to direct the expression of two additional viral protein species. A number of residues within both nsp1β and PCBP2 are required for a nucleic acid–binding event that triggers the frameshifting during PRRSV genome translation. The complex may be dynamic and readily able to assemble and disassemble to interact with the ribosome and thereby facilitate ribosomal stalling, which allows for PRF, and subsequently detach from the genome to allow for downstream translation. Interestingly, sedimentation analysis by analytical ultracentrifugation revealed that nsp1β and PCBP2 each bind to the viral RNA genome as monomers, which is consistent with our structural analysis for the complex by SAXS. Further, the monomeric form of nsp1β in the PRF complex differs from a previously determined X-ray structure of nsp1β, which appears as a dimer. The ability of nsp1β to form a dimer *versus* its monomeric interaction with PCBP2 may underlie a mechanism that regulates the frequency of PRF during virus replication. Future X-ray crystallographic and cryo-EM studies will hopefully reveal the finer structural details of this fascinating example of the noncanonical translation of viral mRNAs.

## Materials and methods

### Expression and purification of nsp1β

Plasmid pGEX-nsp1β WT (GE Healthcare) encoding the nsp1β gene from PRRSV isolate SD01-08 was kindly provided by Dr. Ian Brierley (Department of Pathology, University of Cambridge). The 5′ end of the nsp1β ORF is fused in-frame with a GSH *S*-transferase (GST) affinity tag to assist purification. The plasmid was used to transform *Escherichia coli* BL21 (DE3) GOLD cells (Stratagene). The transformed cells were grown overnight at 37 °C in 20 ml of lysogeny broth (LB) containing 150 μg/ml ampicillin. The overnight culture was used to inoculate 1 liter of fresh ampicillin-containing LB and was subsequently grown at 37 °C with shaking to an *A*_600_ of 0.7–0.8. Expression of the GST-nsp1β fusion protein was then induced by the addition of 0.5 mm isopropyl 1-thio-β-d-galatopyranoside (IPTG) and left to incubate with shaking at 16 °C for an additional 18 h. Cells were then pelleted by centrifugation and stored at −80 °C.

Cell pellets were resuspended in ice-cold lysis buffer (1× PBS, pH 7.4, 300 mm NaCl, 100 mm KCl, 5% glycerol, 2 mm DTT) and lysed using a French pressure cell (AMINCO). Cell lysate was clarified by centrifugation (17,211 × *g* at 4 °C), and the supernatant containing the GST-nsp1β fusion was mixed end-over-end for 1 h at 4 °C with GST-Bind resin (Millipore) that had been pre-equilibrated in lysis buffer. The lysate/resin slurry was poured into a gravity column and washed with 10 column volumes of lysis buffer, followed by elution of the fusion protein with lysis buffer supplemented with 10 mm reduced GSH (adjusted to pH 7.4).

The GST tag was removed from nsp1β using GST-tagged HRV 3C PreScission Protease, which was incubated with the eluted fusion protein in dialysis tubing overnight at 4 °C in 2 liters of lysis buffer lacking additional NaCl. Tag-free nsp1β was separated from free GST and HRV 3C PreScission Protease by passing the dialyzed protein mixture through GST-Bind resin (pre-equilibrated in dialysis buffer). The flow-through contained purified nsp1β, and its concentration was quantified using a NanoDrop instrument (*A*_280_, ε/1000 = 23,786 m^−1^ cm^−1^). Nsp1β variants Y131A and R135A were purified using the same method as described for the WT enzyme.

### Expression and purification of PCBP2

Plasmid pQE-30-PCBP2 (Qiagen) encoding the full-length ORF for human PCBP2 with an in-frame polyHIS tag at its 5′ end was provided by Dr. Ian Brierley (Department of Pathology, University of Cambridge). The plasmid was used to transform *E. coli* M15 (Qiagen). The transformed cells were grown overnight at 37 °C in LB containing both 35 μg/ml kanamycin and 150 μg/ml ampicillin. Subsequent culturing, IPTG-mediated induction of protein expression, cell lysis, and lysate clarification were carried out as described above for nsp1β. Clarified lysate was mixed end-over-end for 1 h at 4 °C with nickel-nitrilotriacetic acid resin (Qiagen) pre-equilibrated with lysis buffer. The lysate/resin slurry was then poured into a gravity column and washed with 10 column volumes of lysis buffer, followed by 10 column volumes of lysis buffer supplemented with 30 mm imidazole, and finally eluted with lysis buffer supplemented with 250 mm imidazole. The eluted protein was dialyzed against 2 liters of buffer (1× PBS, pH 7.4, 100 mm KCl, 5% glycerol) overnight at 4 °C and then further purified by gel filtration using a Superdex75 (GE Healthcare) gel filtration column. The concentration of purified PCBP2 was quantified using a NanoDrop instrument (*A*_280_, ε/1000 = 45,525 m^−1^ cm^−1^). PCBP2 mutants (N325D, R40A/N325D, and R40A/R57A/N325D) were purified using the same method as described for the WT protein.

### Site-directed mutagenesis

Nsp1β variants Y131A and R135A were constructed using round-the-horn site-directed mutagenesis (58) using plasmid pGEX-nsp1β WT as template (phosphorylated primers in Table S1). The linear PCR amplicon was purified (Qiagen), followed by DpnI treatment to remove any plasmid template and then recircularized using instant sticky-end DNA ligase (New England Biolabs). The ligation product was used to transform *E. coli* NEB5α (New England Biolabs). Once successful mutagenesis by DNA sequencing was confirmed, plasmid pGEX-nsp1β Y131A and pGEX-nsp1b R135A were independently transformed into *E. coli* BL21 (DE3) GOLD cells.

PCBP2 variants N325D, R40A/N325D, and R40A/R57A/N325D were constructed using a Q5 site-directed mutagenesis kit (New England Biolabs) and plasmid pQE-30-PCBP2 as template. Primers (Table S1) were designed using the NEBaseChanger tool to produce individual point mutations. Multiple rounds of site-directed mutagenesis were carried out to construct the double and triple PCBP2 mutations. All variants were confirmed by DNA sequencing.

Constructs used in cell culture assays were generated by standard PCR-based mutagenesis and recombinant DNA techniques. Expression vector pL1a was a derivative of an equine arteritis virus ORF1a expression vector, in which the foreign gene is under control of a T7 RNA polymerase promoter and an encephalomyocarditis virus internal ribosomal entry site and is followed by a downstream T7 terminator sequence (59). pCAGGS-nsp1b-WT, R134A, and R135A containing the European PRRSV strain SD01-08 nsp1β sequence were a kind gift from Dr. Ying Fang (Department of Pathobiology, College of Veterinary Medicine, University of Illinois, Urbana-Champaign, IL, USA). The WT nsp1β sequence was amplified using oligonucleotides EUnsp1b-fw and EUnsp1b-rev (all oligonucleotide sequences listed in Table S2), which introduced EcoRI and NcoI sites upstream and XhoI, NotI, and SbfI sites downstream of nsp1β for cloning purposes. The PCR product was transferred to pUC19 vector for PCR-based mutagenesis to create mutants G129A, K130A, Y131A, L132A, Q133A, L136A, Q137A, V138A, R139A, G140A, M141A, and R142A. The WT and mutant sequences were amplified using oligonucleotides 3xFLAG-EUnsp1b-fw and EUnsp1b-rev, which introduced EcoRI and NcoI sites upstream of a 3xFLAG tag and transferred to the pL1a and pCAGGS (Addgene) expression vectors for cloning purposes. Correct introduction of the mutations was verified using Sanger sequencing. pL-EUnsp2 (14), pL-EUnsp1β-2 (14), pLuc-IFN-β (60), and pcDNA-FLAG-MAVS were described elsewhere (61).

### EMSAs

EMSAs were performed using synthetic ssRNA or ssDNA probes (Integrated DNA Technologies). Nsp1β and PCBP2 proteins used in the assays had been previously concentrated to 20 μm and frozen at −80 °C in single-use aliquots. Each protein was thawed and diluted to 2 μm in EMSA reaction buffer (PBS, pH 7.4, 100 mm KCl, 5% glycerol, and 2 mm DTT). Nucleic acid probes were used at a final concentration of 20 nm. Protein(s) and nucleic acid were combined and co-incubated for each reaction with buffer up to 20 μl for 10 min at 30 °C. Following incubation, each reaction was loaded onto a nondenaturing 8% TBE polyacrylamide gel. Electrophoresis was performed for 70 min in ice-cold 0.5× TBE buffer at 140 V. The gel was subsequently stained with SYBR gold (Thermo Fisher Scientific) in 0.5× TBE for 30 min in the dark (to avoid photobleaching) prior to visualization using UV light.

### Cell culture and antibodies

RK-13 and HEK293T cells were cultured essentially as described previously (59, 62). mAb 58-46 (α-EU-nsp2) (14), which recognizes the N-terminal domain of nsp2, nsp2TF, and nsp2N, was a kind gift from Dr. Ying Fang. mAb-FLAG (M2) was from Sigma.

### Radioactive labeling and radioimmunoprecipitation analysis to determine frameshifting efficiencies

The frameshift-stimulating abilities of the nsp1β mutants were determined by transient expression in RK-13 cells, using plasmid pL1a and the recombinant vaccinia virus/T7 polymerase expression system, which was performed essentially as described previously (59) by labeling transfected cells for 2 h using 150 μCi/ml of a [^35^S]Met/Cys mixture (EXPRE35S35SProtein Labeling Mix, PerkinElmer Life Sciences). Cells were transfected with nsp2 alone, nsp2 and WT nsp1β expressed from the same plasmid, or nsp2 and 3xFLAG-nsp1β WT or mutants co-expressed from separate plasmids. Protocols for cell lysis, immunoprecipitation, SDS-PAGE, and quantification with a Typhoon Variable Mode Imager (GE Healthcare) have been described previously (63). Nsp2, nsp2TF, and nsp2N were immunoprecipitated using mouse mAb 58-46, and the 3xFLAG-nsp1β mutants were immunoprecipitated using mouse mAb-FLAG (M2, Sigma). Band intensities (nsp2, nsp2TF, and nsp2N) were quantified with ImageQuant TL (GE Healthcare) and normalized by the Met + Cys content of the respective products (nsp2: 14 Met, 32 Cys; nsp2TF: 14 Met, 24 Cys; and nsp2N: 11 Met, 18 Cys), assuming that [^35^S]Met and [^35^S]Cys are incorporated with an efficiency ratio of 73:22 (the Met/Cys ratio in the mixture according to the manufacturer's documentation). We previously determined that calculated frameshifting efficiencies are only 1.06–1.07 times higher if equal incorporation efficiencies are assumed instead (14). Using these values, frameshifting efficiencies were calculated as (nsp2TF)/(nsp2 + nsp2TF + nsp2N) for −2 frameshifting and (nsp2N)/(nsp2 + nsp2TF + nsp2N) for −1 frameshifting. The experiment was repeated three times.

### Dual-Luciferase assay to determine interferon suppression

To determine the interferon suppression abilities of the nsp1β mutants, 80% confluent HEK293T cells in 24-well plates were transfected using the calcium phosphate transfection method (64). Cells were cotransfected with 5 ng of pRL-TK, encoding *Renilla* luciferase (Promega), 25 ng of pcDNA-FLAG-MAVS to induce an innate immune response, 50 ng of pLuc-IFN-β, firefly reporter, and 75 ng of pCAGGS-3xFLAG-nsp1β expression plasmids. At 18 h post-transfection, cells were harvested, and luciferase expression was measured using the Dual-Luciferase Stop & Glo Reporter Assay System (Promega) and the EnVision Multimode Microplate Reader (PerkinElmer Life Sciences). Experiments were performed in triplicate and independently repeated three times. Firefly luciferase activity was normalized by dividing the activity by the *Renilla* luciferase activity in the same well. Statistical significance (*p* < 0.001) was determined using an unpaired two-tailed Student's *t* test in GraphPad Prism 8.1.1 (GraphPad Software, San Diego, CA, USA).

### Large-scale purification of the trimeric complexes for AUC and SAXS

Low concentrations of equimolar nsp1β and PCBP2 in PBS (pH 7.4), 100 mm KCl, and 5% glycerol were co-incubated with a 1.1-fold molar excess of DNA/RNA probe for 3 h at 4 °C. Following incubation, the protein–nucleic acid complex was concentrated using a centrifugal filter unit (Amicon) to a volume of 2 ml, which was loaded onto a Superdex200 (GE Healthcare) gel filtration column and purified. The integrity of purified complexes was evaluated by SDS-PAGE, nondenaturing TBE PAGE, and dynamic light scattering prior to further analysis by analytical ultracentrifugation and small-angle X-ray scattering.

### Characterization of the nsp1β:PCBP2:ssRNA complex by sedimentation velocity

Sedimentation velocity data were collected on a Beckman-Coulter ProteomeLab™ XL-I analytical ultracentrifuge equipped with an 8-hole An50Ti rotor. All samples were temperature-equilibrated in the rotor for at least 2 h under vacuum.

Seven samples of 34-nt ssRNA at 8 μm (1×), 16 μm (2×), 32 μm (2×), and 64 μm (2×) concentrations were centrifuged at rotor speeds of 42,000 rpm (samples ≥ 16 μm) or 30,000 rpm (8 μm) for 24 h at 20 °C in the specified solvent. The concentration gradients in the cell were monitored by the absorbance optics at wavelengths of 278 nm (8 μm sample), 294 nm (samples ≥16 μm, first run), and 291 nm (samples ≥16 μm, second run).

SEC-purified triple complex was prepared at 1.00, 2.00, and 4.00 mg/ml total material concentration. Each sample was centrifuged at 30,000 rpm for 24 h at 20 °C in the specified solvent. The concentration gradients in the cells were monitored by the interference optics and by the absorbance optics at 300-nm wavelength. Due to its 1-order of magnitude higher absorption coefficient, the RNA dominated the signal in the absorbance optics. By contrast, the interference optics could detect all components. We repeated the experiment using the same samples in the same cells at 42,000 rpm. Comparing the signal *versus* loading concentrations of both runs, we detected that we had suffered material loss due to aggregation at the bottom of the cells. Actual sample concentrations in the second run had reduced to 0.44, 0.75, and 1.09 mg/ml.

The *c*(*s, f_r_*) analysis (65) and *c*(*s*) analysis (66) were performed in SEDFIT. Direct fitting to the LAMM equation of sedimentation coefficient *s* and diffusion coefficient *D* of the observed species was executed in SEDPHAT using the *Global Discrete Species* model (41). In practice, due to the design of the software, we fitted *s*_20,_*_w_* and *M* using a substitute $\bar{v}$ of 0.73 and then converted the values back to experimental *s_e_* and *D_e_*. 95% confidence intervals of the fitted parameters were determined in SEDPHAT (automatic confidence interval search with projection method (67, 68, 69)). The partial specific volume $\bar{v}$ of the protein components was calculated using the program SEDNTERP 2 (42). The results were plotted using QTIPLOT (68), GUSSI (70), and MATPLOTLIB (71).

### Measurement of the volumetric mass density of the solvent for sedimentation velocity

The density of the solvent was measured using a 1.000–1.220 floating hydrometer (Ertco, Wertheim, Germany) in a temperature-controlled room. Six independent measurements were taken. We measured three independent preparations of the buffer solution to assess variations between them. The confidence intervals in Table S3 include the deviations between the three preparations. The instrument yields the viscosity relative to pure water (ρ*_r_*) and the readings were converted to absolute density (ρ), using the volumetric mass density of water (ρ*_w_* = 0.998234 g/cm^3^).

(Eq. 1) $$\rho =\rho_{r}\times \rho_{w}$$

### Solvent viscosity measurements for sedimentation velocity

The viscosity of the solvent was measured using an SV-10 tuning fork vibro viscometer (A&D Company, Missisauga, Canada) in a temperature-controlled room set to 19 ± 1 °C. The sample cup was filled with 40.0-ml ultrapure water, and the pedestal height was adjusted until the water surface reached the tapered region on the sensor plates. The instrument was then calibrated with this water using the “Simplified Calibration” function. The water was carefully drained from the sample cup using a syringe with an attached flexible tube without disturbing the geometry of the setup (such as lowering the pedestal or moving the sample cup or shifting the sensor protector). 40.0 ml of solvent with a temperature of approximately 21 °C was then added to the sample cup (without disturbing the geometry) and allowed to cool while continuously monitoring the viscosity every 15 s using the RsVisco control program. All measurement values from 20.1 to 19.9 °C were selected and averaged. The calibration/measurement cycle was repeated six times, yielding six independent measurements. We measured three independent preparations of the buffer solution to assess variations between them. The confidence intervals in Table S3 include the deviations between the three preparations. The instrument yields the viscosity relative to pure water (η*_r_*), which had to be converted to absolute viscosity (η).

(Eq. 2) $$\eta =\eta_{r}/\rho_{r}$$

### SAXS data collection and processing

The SAXS data for nsp1β:PCBP2:ssRNA complex was collected and processed as described previously (72, 73) at the B21 beamline at Diamond Light Source (Didcot, Oxfordshire, UK). Briefly, 50 μl of the 10 mg/ml complex was injected into a 4.5-ml Shodex KW40 size exclusion column connected to an in-line Agilent 1200 (Agilent Technologies, Stockport, UK) HPLC, a flow cell, and an Eiger 4M X-ray detector. We collected ∼600 frames where each frame was exposed to the X-rays for 3 s. Using the ATSAS version 2.8 software package (74), the peak region was buffer-subtracted and merged using Primus (75), followed by Guinier analysis of merged data. Dimensionless Kratky analysis was also performed to ensure that the complex was folded. The pair-distance distribution (*P*(*r*)) analysis was performed using the program GNOM (49) to obtain the *R_g_* and *D*_max_. Next, we calculated 20 low-resolution structures using the *P*(*r*) information and program DAMMIN (50). Last, the 20 low-resolution structures were averaged and filtered to obtain a representative structure using the DAMAVER package (53), as described previously (72).

### SAXS envelope fitting

The experimentally determined envelope was used to manually fit in preexisting protein structures and a modeled nucleic acid molecule representing the 34-nt ssRNA. The crystal structure of nsp1β from PRRSV strain XH-GD (PDB entry 3MTV (22); *purple* with the RBM helix in *red*) was fit in as a monomer. The NMR solution structure of the KH1-KH2 fusion was also fit in (PDB entry 2JZX (24); *yellow* and *pink*, respectively). The third KH domain was fit in from an existing crystal structure bound to C-rich DNA with the nucleic acid removed (PDB entry 2P2R; *teal*). RNA in *green* is from a structure of KH1 bound to C-rich RNA as a reference (PDB entry 2PY9 (23)). The 34-nt ssRNA molecule (*orange* with C-rich motif shown in *blue*) was modeled using w3DNA 2.0 (56) and subsequently fit into the experimentally determined density. All fitting was completed in PyMOL (27).

## Data availability

X-ray/NMR structures reported in this paper are available in the Protein Data Bank (PDB). All other data are presented in the article.

## Author Profile

Ankoor Patel

## References

[1] Firth A.E., Brierley I. (2012). Non-canonical translation in RNA viruses. J. Gen. Virol.

[2] Atkins J.F., Loughran G., Bhatt P.R., Firth A.E., Baranov P.V. (2016). Ribosomal frameshifting and transcriptional slippage: from genetic steganography and cryptography to adventitious use. Nucleic Acids Res.

[3] Somogyi P., Jenner A.J., Brierley I., Inglis S.C. (1993). Ribosomal pausing during translation of an RNA pseudoknot. Mol. Cell Biol.

[4] Kontos H., Napthine S., Brierley I. (2001). Ribosomal pausing at a frameshifter RNA pseudoknot is sensitive to reading phase but shows little correlation with frameshift efficiency. Mol. Cell Biol.

[5] Brierley I., Digard P., Inglis S. (1989). Characterization of an efficient coronavirus ribosomal frameshifting signal: requirement for an RNA pseudoknot. Cell.

[6] Giedroc D., Cornish P. (2009). Frameshifting RNA pseudoknots: structure and mechanism. Virus Res.

[7] Jacks T., Varmus H.E. (1985). Expression of the Rous sarcoma virus pol gene by ribosomal frameshifting. Science.

[8] Jacks T., Madhani H.D., Masiarz F.R., Varmus H.E. (1988). Signals for ribosomal frameshifting in the Rous sarcoma virus gag-pol region. Cell.

[9] Jacks T., Townsley K., Varmus H.E., Majors J. (1987). Two efficient ribosomal frameshifting events are required for synthesis of mouse mammary tumor virus gag-related polyproteins. Proc. Natl. Acad. Sci. U. S. A.

[10] Tu C., Tzeng T.H., Bruenn J.A. (1992). Ribosomal movement impeded at a pseudoknot required for frameshifting. Proc. Natl. Acad. Sci. U. S. A.

[11] Snijder E.J., Kikkert M., Fang Y. (2013). Arterivirus molecular biology and pathogenesis. J. Gen. Virol.

[12] den Boon J.A., Snijder E.J., Chirnside E.D., de Vries A.A., Horzinek M.C., Spaan W.J. (1991). Equine arteritis virus is not a togavirus but belongs to the coronaviruslike superfamily. J. Virol.

[13] Li Y., Treffers E.E., Napthine S., Tas A., Zhu L., Sun Z., Bell S., Mark B.L., Veelen P.A., van Hemert M.J., Firth A.E., Brierley I., Snijder E.J., Fang Y. (2014). Transactivation of programmed ribosomal frameshifting by a viral protein. Proc. Natl. Acad. Sci. U. S. A.

[14] Fang Y., Treffers E.E., Li Y., Tas A., Sun Z., van der Meer Y., de Ru A.H., van Veelen P.A., Atkins J.F., Snijder E.J., Firth A.E. (2012). Efficient -2 frameshifting by mammalian ribosomes to synthesize an additional arterivirus protein. Proc. Natl. Acad. Sci. U. S. A.

[15] Li Y., Shang P., Shyu D., Carrillo C., Naraghi-Arani P., Jaing C.J., Renukaradhya G.J., Firth A.E., Snijder E.J., Fang Y. (2018). Nonstructural proteins nsp2TF and nsp2N of porcine reproductive and respiratory syndrome virus (PRRSV) play important roles in suppressing host innate immune responses. Virology.

[16] Li Y., Firth A.E., Brierley I., Cai Y., Napthine S., Wang T., Yan X., Kuhn J.H., Fang Y. (2019). Programmed −2/−1 ribosomal frameshifting in Simarteriviruses: an evolutionarily conserved mechanism. J. Virol.

[17] Napthine S., Treffers E.E., Bell S., Goodfellow I., Fang Y., Firth A.E., Snijder E.J., Brierley I. (2016). A novel role for poly(C) binding proteins in programmed ribosomal frameshifting. Nucleic Acids Res.

[18] Beura L.K., Dinh P.X., Osorio F.A., Pattnaik A.K. (2011). Cellular poly(C) binding proteins 1 and 2 interact with porcine reproductive and respiratory syndrome virus nonstructural protein 1β and support viral replication. J. Virol.

[19] Lunney J.K., Fang Y., Ladinig A., Chen N., Li Y., Rowland B., Renukaradhya G.J. (2016). Porcine reproductive and respiratory syndrome Virus (PRRSV): pathogenesis and interaction with the immune system. Annu. Rev. Anim. Biosci.

[20] Hu J., Zhang C. (2014). Porcine reproductive and respiratory syndrome virus vaccines: current status and strategies to a universal vaccine. Transbound. Emerg. Dis.

[21] Sattler T., Pikalo J., Wodak E., Revilla-Fernández S., Steinrigl A., Bagó Z., Entenfellner F., Claude J.B., Pez F., Francillette M., Schmoll F. (2018). Efficacy of live attenuated porcine reproductive and respiratory syndrome virus 2 strains to protect pigs from challenge with a heterologous Vietnamese PRRSV 2 field strain. BMC Vet. Res.

[22] Xue F., Sun Y., Yan L., Zhao C., Chen J., Bartlam M., Li X., Lou Z., Rao Z. (2010). The crystal structure of porcine reproductive and respiratory syndrome virus nonstructural protein Nsp1β reveals a novel metal-dependent nuclease. J. Virol.

[23] Du Z., Lee J.K., Fenn S., Tjhen R., Stroud R.M., James T.L. (2007). X-ray crystallographic and NMR studies of protein-protein and protein-nucleic acid interactions involving the KH domains from human poly(C)-binding protein-2. RNA.

[24] Du Z., Fenn S., Tjhen R., James T.L. (2008). Structure of a construct of a human poly(C)-binding protein containing the first and second KH domains reveals insights into its regulatory mechanisms. J. Biol. Chem.

[25] Fenn S., Du Z., Lee J.K., Tjhen R., Stroud R.M., James T.L. (2007). Crystal structure of the third KH domain of human poly (C) -binding protein-2 in complex with a C-rich strand of human telomeric DNA at 1. 6 Å resolution. Nucleic Acids Res.

[26] Du Z., Lee J.K., Tjhen R., Li S., Pan H., Stroud R.M., James T.L. (2005). Crystal structure of the first KH domain of human poly(C)-binding protein-2 in complex with a C-rich strand of human telomeric DNA at 1.7 Å. J. Biol. Chem.

[27] DeLano W.L. (2015). The PyMOL Molecular Graphics System.

[28] Ericsson U.B., Hallberg B.M., DeTitta G.T., Dekker N., Nordlund P. (2006). Thermofluor-based high-throughput stability optimization of proteins for structural studies. Anal. Biochem.

[29] Napthine S., Ling R., Finch L.K., Jones J.D., Bell S., Brierley I., Firth A.E. (2017). Protein-directed ribosomal frameshifting temporally regulates gene expression. Nat. Commun.

[30] Tomonaga T., Levens D. (1995). Heterogeneous nuclear ribonucleoprotein K is a DNA-binding transactivator. J. Biol. Chem.

[31] Dickey T.H., Altschuler S.E., Wuttke D.S. (2013). Single-stranded DNA-binding proteins: multiple domains for multiple functions. Structure.

[32] Makeyev A.V., Liebhaber S.A. (2002). The poly(C)-binding proteins: a multiplicity of functions and a search for mechanisms. RNA.

[33] Li Y., Zhu L., Lawson S.R., Fang Y. (2013). Targeted mutations in a highly conserved motif of the nsp1 β protein impair the interferon antagonizing activity of porcine reproductive and respiratory syndrome virus. J. Gen. Virol.

[34] Han M., Ke H., Zhang Q., Yoo D. (2017). Nuclear imprisonment of host cellular mRNA by nsp1β protein of porcine reproductive and respiratory syndrome virus. Virology.

[35] Beura L.K., Sarkar S.N., Kwon B., Subramaniam S., Jones C., Pattnaik A.K., Osorio F.A. (2010). Porcine reproductive and respiratory syndrome virus nonstructural protein 1 modulates host innate immune response by antagonizing IRF3 activation. J. Virol.

[36] van Kasteren P.B., Beugeling C., Ninaber D.K., Frias-Staheli N., van Boheemen S., Garcia-Sastre A., Snijder E.J., Kikkert M. (2012). Arterivirus and nairovirus ovarian tumor domain-containing deubiquitinases target activated RIG-I to control innate immune signaling. J. Virol.

[37] Sun Z., Chen Z., Lawson S.R., Fang Y. (2010). The cysteine protease domain of porcine reproductive and respiratory syndrome virus nonstructural protein 2 possesses deubiquitinating and interferon antagonism functions. J. Virol.

[38] Chen Z., Lawson S., Sun Z., Zhou X., Guan X., Christopher-Hennings J., Nelson E.A., Fang Y. (2010). Identification of two auto-cleavage products of nonstructural protein 1 (nsp1) in porcine reproductive and respiratory syndrome virus infected cells: nsp1 function as interferon antagonist. Virology.

[39] Han M., Kim C.Y., Rowland R.R.R., Fang Y., Kim D., Yoo D. (2014). Biogenesis of non-structural protein 1 (nsp1) and nsp1-mediated type I interferon modulation in arteriviruses. Virology.

[40] Li Y., Shyu D.-L., Shang P., Bai J., Ouyang K., Dhakal S., Hiremath J., Binjawadagi B., Renukaradhya G.J., Fang Y. (2016). Mutations in a highly conserved motif of nsp1β protein attenuate the innate immune suppression function of porcine reproductive and respiratory syndrome virus. J. Virol.

[41] Schuck P. (1998). Sedimentation analysis of noninteracting and self-associating solutes using numerical solutions to the Lamm equation. Biophys. J.

[42] Tucker H., Wright A., Deubler G., Bashir B., Hayes D.B., Laue T.M., Philo J. (2013). SEDNTERP 2: Sedimentation Interpretation Program.

[43] Madeira F., Park Y.M., Lee J., Buso N., Gur T., Madhusoodanan N., Basutkar P., Tivey A.R.N., Potter S.C., Finn R.D., Lopez R. (2019). The EMBL-EBI search and sequence analysis tools APIs in 2019. Nucleic Acids Res.

[44] Krissinel E., Henrick K. (2007). Inference of macromolecular assemblies from crystalline state. J. Mol. Biol.

[45] Guinier A., Fournet G., Walker C.B., Vineyard G.H. (1956). Small-angle scattering of X-rays. Phys. Today.

[46] Pérez J., Vachette P. (2017). A successful combination: coupling SE-HPLC with SAXS. Adv. Exp. Med. Biol.

[47] Patel T.R., Chojnowski G., Astha, Koul A., McKenna S.A., Bujnicki J.M. (2017). Structural studies of RNA-protein complexes: a hybrid approach involving hydrodynamics, scattering, and computational methods. Methods.

[48] Durand D., Vivès C., Cannella D., Pérez J., Pebay-Peyroula E., Vachette P., Fieschi F. (2010). NADPH oxidase activator p67phox behaves in solution as a multidomain protein with semi-flexible linkers. J. Struct. Biol.

[49] Svergun D.I. (1992). Determination of the regularization parameter in indirect-transform methods using perceptual criteria. J. Appl. Crystallogr.

[50] Svergun D.I. (1999). Restoring low resolution structure of biological macromolecules from solution scattering using simulated annealing. Biophys. J.

[51] Patel T.R., Bernards C., Meier M., McEleney K., Winzor D.J., Koch M., Stetefeld J. (2014). Structural elucidation of full-length nidogen and the laminin-nidogen complex in solution. Matrix Biol.

[52] Mrozowich T., Henrickson A., Demeler B., Patel T.R. (2020). Nanoscale structure determination of Murray Valley encephalitis and Powassan virus non-coding RNAs. Viruses.

[53] Volkov V.V., Svergun D.I. (2003). Uniqueness of *ab initio* shape determination in small-angle scattering. J. Appl. Cryst.

[54] Ortega A., Amorós D., García de la Torre J. (2011). Prediction of hydrodynamic and other solution properties of rigid proteins from atomic- and residue-level models. Biophys. J.

[55] Meier M., Moya-Torres A., Krahn N.J., McDougall M.D., Orriss G.L., McRae E.K.S., Booy E.P., McEleney K., Patel T.R., McKenna S.A., Stetefeld J. (2018). Structure and hydrodynamics of a DNA G-quadruplex with a cytosine bulge. Nucleic Acids Res.

[56] Li S., Olson W.K., Lu X.J. (2019). Web 3DNA 2.0 for the analysis, visualization, and modeling of 3D nucleic acid structures. Nucleic Acids Res.

[57] Petoukhov M.V., Franke D., Shkumatov A.V., Tria G., Kikhney A.G., Gajda M., Gorba C., Mertens H.D.T., Konarev P.V., Svergun D.I. (2012). New developments in the *ATSAS* program package for small-angle scattering data analysis. J. Appl. Crystallogr.

[58] Moore S.D., Prevelige P.E. (2002). A P22 scaffold protein mutation increases the robustness of head assembly in the presence of excess portal protein. J. Virol.

[59] Snijder E.J., Wassenaar A.L., Spaan W.J. (1994). Proteolytic processing of the replicase ORF1a protein of equine arteritis virus. J. Virol.

[60] Fitzgerald K.A., McWhirter S.M., Faia K.L., Rowe D.C., Latz E., Golenbock D.T., Coyle A.J., Liao S.M., Maniatis T. (2003). IKKE and TBKI are essential components of the IRF3 signalling pathway. Nat. Immunol.

[61] Seth R.B., Sun L., Ea C.K., Chen Z.J. (2005). Identification and characterization of MAVS, a mitochondrial antiviral signaling protein that activates NF-κB and IRF3. Cell.

[62] Bailey-Elkin B.A., Knaap R.C.M., Johnson G.G., Dalebout T.J., Ninaber D.K., Van Kasteren P.B., Bredenbeek P.J., Snijder E.J., Kikkert M., Mark B.L. (2014). Crystal structure of the middle east respiratory syndrome coronavirus (MERS-CoV) papain-like protease bound to ubiquitin facilitates targeted disruption of deubiquitinating activity to demonstrate its role in innate immune suppression. J. Biol. Chem.

[63] Li Y., Tas A., Snijder E.J., Fang Y. (2012). Identification of porcine reproductive and respiratory syndrome virus ORF1a-encoded non-structural proteins in virus-infected cells. J. Gen. Virol.

[64] Graham F.L., Van der Eb A. (1973). A new technique for the assay of infectivity of human adenovirus 5 DNA. Virology.

[65] Brown P.H., Schuck P. (2006). Macromolecular size-and-shape distributions by sedimentation velocity analytical ultracentrifugation. Biophys. J.

[66] Schuck P. (2000). Size-distribution analysis of macromolecules by sedimentation velocity ultracentrifugation and Lamm equation modeling. Biophys. J.

[67] Johnson M.L., Straume M., Schuster T.M., Laue T.M. (1994). Comments on the analysis of sedimentation equilibrium experiments. Modern Analytical Ultracentrifugation: Emerging Biochemical and Biophysical Techniques.

[68] Straume M., Veldhuis J.D., Johnson M.L. (1994). Model-independent quantification of measurement error: empirical estimation of discrete variance function profiles based on standard curves. Methods Enzymol.

[69] Bevington P.R., Robinson D.K. (2003). Data Reduction and Error Analysis for the Physical Sciences.

[70] Brautigam C.A. (2015). Calculations and publication-quality illustrations for analytical ultracentrifugation data. Methods Enzymol.

[71] Hunter J.D. (2007). Matplotlib: a 2D graphics environment. Comput. Sci. Eng.

[72] Kim D.N., Thiel B.C., Mrozowich T., Hennelly S.P., Hofacker I.L., Patel T.R., Sanbonmatsu K.Y. (2020). Zinc-finger protein CNBP alters the 3-D structure of lncRNA Braveheart in solution. Nat. Commun.

[73] Yazdi M.M., Saran S., Mrozowich T., Lehnert C., Patel T.R., Sanders D.A.R., Palmer D.R.J. (2020). Asparagine-84, a regulatory allosteric site residue, helps maintain the quaternary structure of *Campylobacter jejuni* dihydrodipicolinate synthase. J. Struct. Biol.

[74] Franke D., Petoukhov M.V., Konarev P.V., Panjkovich A., Tuukkanen A., Mertens H.D.T., Kikhney A.G., Hajizadeh N.R., Franklin J.M., Jeffries C.M., Svergun D.I. (2017). *ATSAS 2.8*: a comprehensive data analysis suite for small-angle scattering from macromolecular solutions. J. Appl. Crystallogr.

[75] Konarev P.V., Volkov V.V., Sokolova A.V., Koch M.H.J., Svergun D.I. (2003). PRIMUS: a Windows PC-based system for small-angle scattering data analysis. J. Appl. Crystallogr.

