# Supplementary material for: Molecular characterization of the RNA-protein complex directing −2/−1 programmed ribosomal frameshifting during arterivirus replicase expression
Source: J Biol Chem. 2021 Jan 13;295(52):17904–21. doi: 10.1074/jbc.RA120.016105 (PMC7939443; doi:10.1074/jbc.RA120.016105)
Supplement: Supplementary file 1 [file mmc1.pdf]

Molecular characterization of the RNA-protein complex directing -2/-1 programmed ribosomal frameshifting during arterivirus replicase expression

Ankoor Patel, Emmely E. Treffers, Markus Meier, Trushar R. Patel, Jörg Stetefeld, Eric J. Snijder, Brian L. Mark[1*]

Supporting Information

Table S1. Primers used for nsp1β and PCBP2 mutations for EMSAs

Oligo Name	Sequence (5'to 3')
nsp1β Y131A Forward	CCAACGCAGGCTTCAAGTCCG
nsp1β Y131A Reverse	AGCGCCTTACCATGCACGCCCC
nsp1β R135A Forward	CAAGTCCGCGGCATGCGTGC
nsp1β R135A Reverse	AGCGCGCGTTGGAGGTACTTAC
PCBP2 R40A Forward	GGAGAGTGGTGCACGTATCAAC
PCBP2 R40A Reverse	TCCGCCATCTTCTTAACTGATTC
PCBP2 R57A Forward	TATCACTTTGGCTGGACCCAC
PCBP2 R57A Reverse	ATCGCCTCAGGACAATTCCCTTC
PCBP2 N325D Forward	CAAAATTGCGGATCCAGTGGAAGG
PCBP2 N325D Reverse	ATCTGCGCCCCAGACATC

Table S2. Primers used for frameshifting assays

Oligo Name	Sequence (5' to 3')	Purpose
EUnsp1b-G129A-fw	GGGCGTGCATGCTAAGTACCTCCAACGCAG	Site directed mutagenesis G129A
EUnsp1b-G129A-rev	TGGAGGTACTTAGCATGCACGCCCCACTTGG	Site directed mutagenesis G129A
EUnsp1b-K130A-fw	GGCGTGCATGGTGCUTACCTCCAACGCAG	Site directed mutagenesis K130A
EUnsp1b-K130A-rev	TGCGTTGGAGGTAAGCACCATGCACGCC	Site directed mutagenesis K130A
EUnsp1b-Y131A-fw	TGCATGGTAAGGCCCTCCAACGCAGGCTTC	Site directed mutagenesis Y131A
EUnsp1b-Y131A-rev	TGCGTTGGAGGGCCTTACCATGCACGCCCC	Site directed mutagenesis Y131A
EUnsp1b-L132A-fw	GCATGGTAAGTACGCCCAACGCAGGCTTCAAGTC	Site directed mutagenesis L132A
EUnsp1b-L132A-rev	GAAGCCTGCGTTGGGCGTACTTACCATGCACG	Site directed mutagenesis L132A
EUnsp1b-Q133A-fw	TAAGTACCTCGCACGCAGGCTTCAAGTCCG	Site directed mutagenesis Q133A
EUnsp1b-Q133A-rev	GAAGCCTGCGTGCGAGGTACTTACCATGCAC	Site directed mutagenesis Q133A

EUnsp1b-L136A-fw	CCAACGCAGGGCTCAAGTCCGCGGCATGC	Site directed mutagenesis L136A
EUnsp1b-L136A-rev	CATGCCGCGGACTTGAGCCCTGCGTTGGAG	Site directed mutagenesis L136A
EUnsp1b-Q137A-fw	TCCAACGCAGGCTTGCAGTCCGCGGCATGC	Site directed mutagenesis Q137A
EUnsp1b-Q137A-rev	CCGCGGACTGCAAGCCTGCGTTGGAGGTAC	Site directed mutagenesis Q137A
EUnsp1b-V138A-fw	GCAGGCTTCAAGCCCGCGGCATGCGTGCTG	Site directed mutagenesis V138A
EUnsp1b-V138A-rev	GCATGCCGCGGGCTTGAAGCCTGCGTTGGAG	Site directed mutagenesis V138A
EUnsp1b-R139A-fw	CTTCAAGTCGCTGGCATGCGTGCTGTGGTC	Site directed mutagenesis R139A
EUnsp1b-R139A-rev	CACAGCACGCATGCCAGCGACTTGAAGCC	Site directed mutagenesis R139A
EUnsp1b-G140A-fw	TTCAAGTCCGCGCCATGCGTGCTGTGGTCG	Site directed mutagenesis G140A
EUnsp1b-G140A-rev	CAGCACGCATGGCGCGGACTTGAAGCCTGC	Site directed mutagenesis G140A

EUnsp1b-M141A-fw	AGTCCGCGGCGCTCGTGCTGTGGTCGATCC	Site directed mutagenesis M141A
EUnsp1b-M141A-rev	CCACAGCACGAGCGCCGCGGACTTGAAGCC	Site directed mutagenesis M141A
EUnsp1b-R142A-fw	CCGCGGCATGGCTGCTGTGGTCGATCCTG	Site directed mutagenesis R142A
EUnsp1b-R142A-rev	CGACCACAGCAGCCATGCCGCGGACTTGAAG	Site directed mutagenesis R142A
EUnsp1b-fw	ACGAGAATTCCCATGGATGTCTGACGTTTACAG GTGGAAG	Amplify EUnsp1b and introduce upstream EcoRI and NcoI sites
3xFLAG-EUnsp1b-fw	GCCGAATTCCCATGGACTACAAAGACCATGACG GTGATTATAAAGATCATGACATCGATTACAAGG ATGACGATGACAAGTCTGACGTTTACAGGTGGA AG	Amplify EUnsp1b and introduce upstream 3xFLAG tag and NcoI site
EUnsp1b-rev	ATCACCTGCAGGGCGGCCGCCTCGAGTCAGCCA TACCACTTATGTG	Amplify EUnsp1b and introduce downstream XhoI, NotI and SbfI restriction sites
pL-seq	GATCTGATCTGGGGCCTC	Sequencing pL vector

Part 2

Biophysical characterization

Parameters	
**Solvent**<br>**1x PBS (pH 7.4 @ 25°C), 100 mM KCl, 5% glycerol**	2.027 mM sodium dihdyrogen phosphate<br>7.973 mM disodium hydrogen phosphate<br>138 mM sodium chloride<br>102.7 mM potassium chloride<br>~684 mM glycerol
**Solvent density[1] ρ (g/cm^3)**	1.0354 ± 0.0005
**Buffer viscosity[1] η (P)**	0.01235 ± 0.0002
**Temperature T (°C)**	20.0
**his$_6$-PCBP2 partial specific volume[1] $\bar{\nu}$ (cm^3/g)**	0.73318
**his$_6$-PCBP2 formula mass[2] M_w (kDa)**	39749.32
**his$_6$- PCBP2 absorption coefficient[2] ε_{280} (M^{-1} cm^{-1})**	14815
**PRRSV nsp1β partial specific volume[1] $\bar{\nu}$ (cm^3/g)**	0.72777
**PRRSV nsp1β formula mass[2] M_w (kDa)**	23786.87
**PRRSV nsp1β absorption coefficient[2] ε_{280} (M^{-1} cm^{-1})**	46200
**34nt ssRNA partial specific volume $\bar{\nu}$ (cm^3/g)**	dependent on cation concentration<br>expected range 0.50 - 0.65
**34nt ssRNA formula mass[3] M_w (kDa)**	10688.3
**34nt ssRNA absorption coefficient[3] ε_{260} (M^{-1} cm^{-1})**	323000

[1]Sednterp, [2]ProtParam, [3]IDTDNA

Table S3

Section 1:
Characterization of 34 nt ssRNA by Sedimentation Velocity

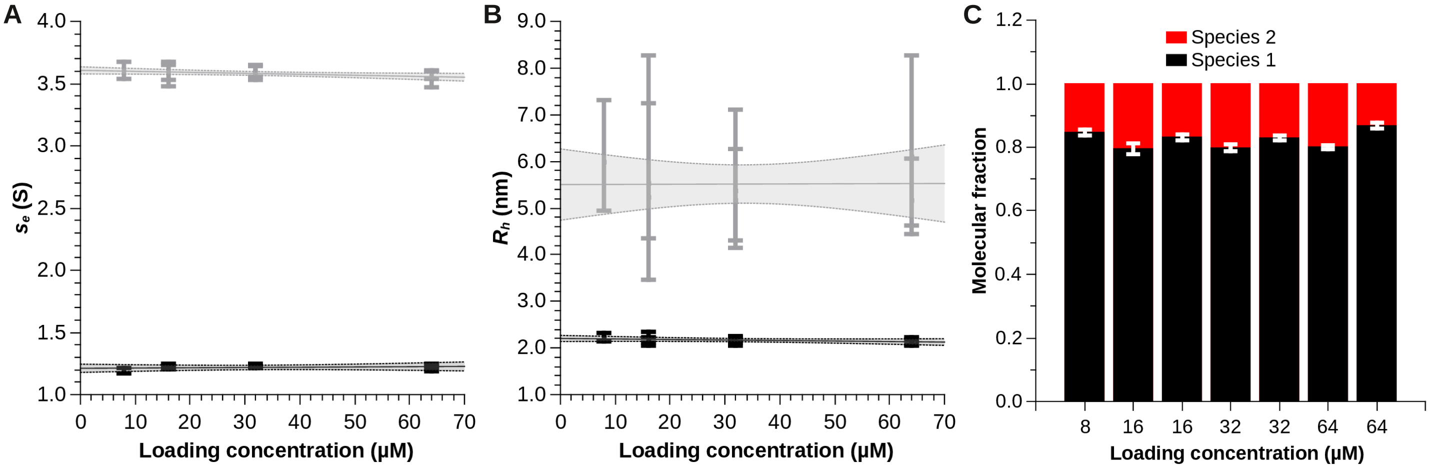

Fig. S1

Results

Species 1	
**Sedimentation coefficient s^0_e**	1.21 ± 0.04 S
**Diffusion coefficient D^0_e**	7.9 ± 0.3 ·10⁻⁷ cm²/s
**Partial specific volume** that yields correct mass (10.7 kDa)	0.62763 [0.60703 - 0.64704] cm^3/g
**Sedimentation coefficient $s^0_{20°C, w}$**	1.59 [1.54 - 1.65] S
**Hydrodynamic radius R_h^0**	2.20 [2.12 - 2.29] nm
Species 2	
**Sedimentation coefficient s^0_e**	3.61 ± 0.03 S
**Diffusion coefficient D^0_e**	3.2 ± 0.5 ·10⁻⁷ cm²/s
**Sedimentation coefficient $s^0_{20°C, w}$**	4.74 [4.65 - 4.83] S
**Hydrodynamic radius R_h^0**	5.49 [4.84 - 6.35] nm
**Molecular mass** if RNA; would contain 7-8 strands	79.3 [68.2 - 90.5] kDa

Uncertainties are given as 95% confidence intervals

Table S4

8 µM ssRNA monitored by absorbance optics
30000 rpm, λ = 278 nm

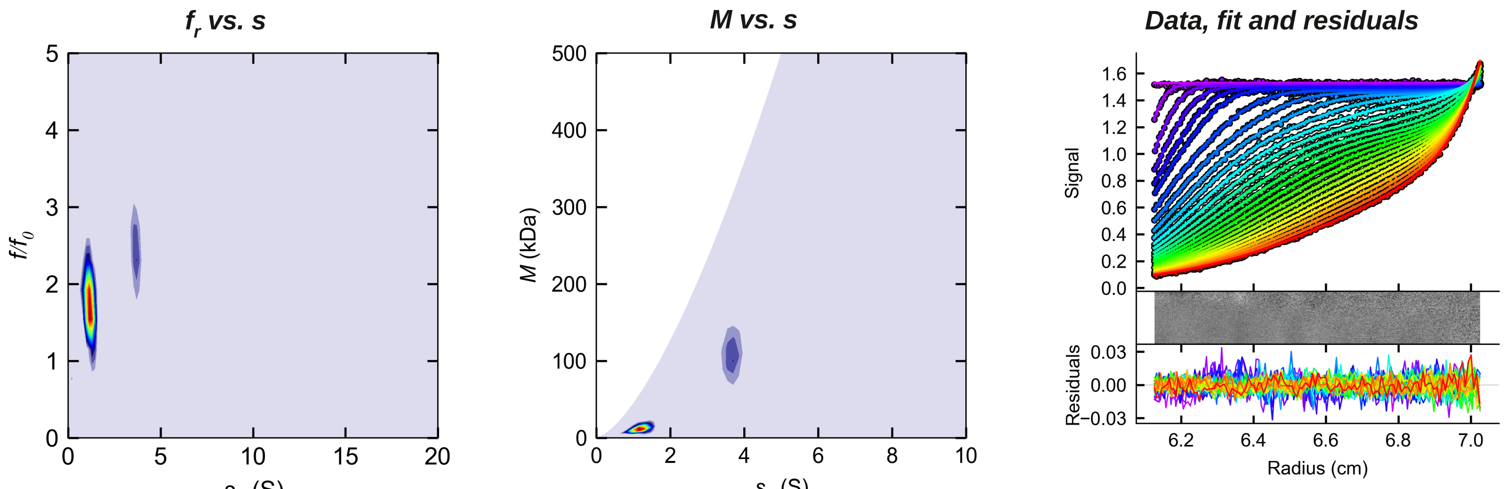

Reduced χ² [r.m.s.d]: 0.546565 [0.007393]

Species Analysis (30000 rpm, λ = 278 nm, exp. # 1)

Parameter	Value	95% confidence interval
Species 1		
**Sedimentation coefficient s_e (S)**	1.191056	1.1725 - 1.2098
**Diffusion coefficient D_e ($\cdot 10^{-7}$ cm²/s)**	7.826990	7.5122 - 8.1480
Species 2		
**Sedimentation coefficient s_e (S)**	3.605013	3.5373 - 3.6741
**Diffusion coefficient D_e ($\cdot 10^{-7}$ cm²/s)**	2.905149	2.3804 - 3.5159
**Fraction (signal)**	0.153196	0.1439 - 0.1631
Common		
**Total concentration (signal)**	1.508362	1.4841 - 1.5328
**Meniscus position (cm)**	6.081005	6.0774 - 6.0844
**Bottom position (cm)**	7.237708	7.2307 - 7.2447
**Reduced χ2 of fit [rmsd]**	0.5652639	
**R.m.s.d. of fit**	0.007518403	

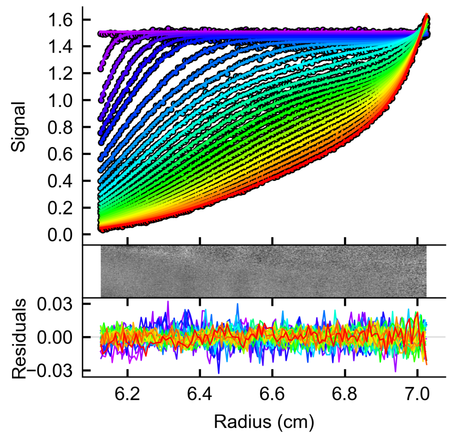

Reduced χ² [r.m.s.d]: 0.565264 [0.007518]

16 μM ssRNA monitored by absorbance optics

42000 rpm, λ = 294 nm

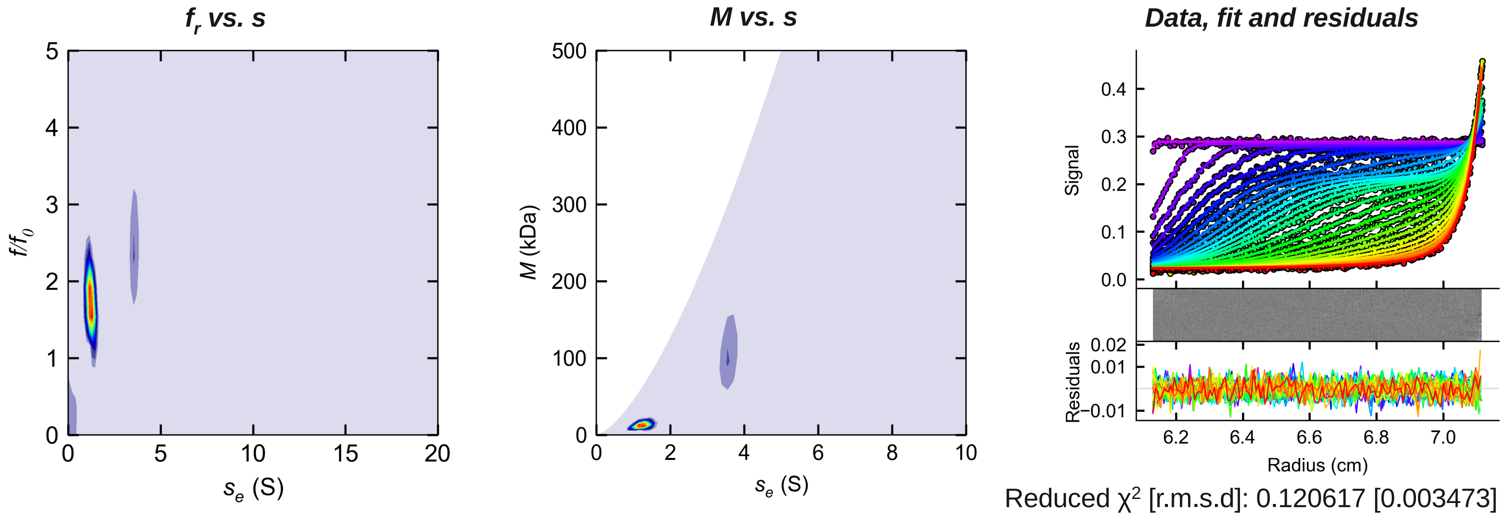

Reduced χ^2 [r.m.s.d]: 0.120617 [0.003473]

42000 rpm, λ = 291 nm

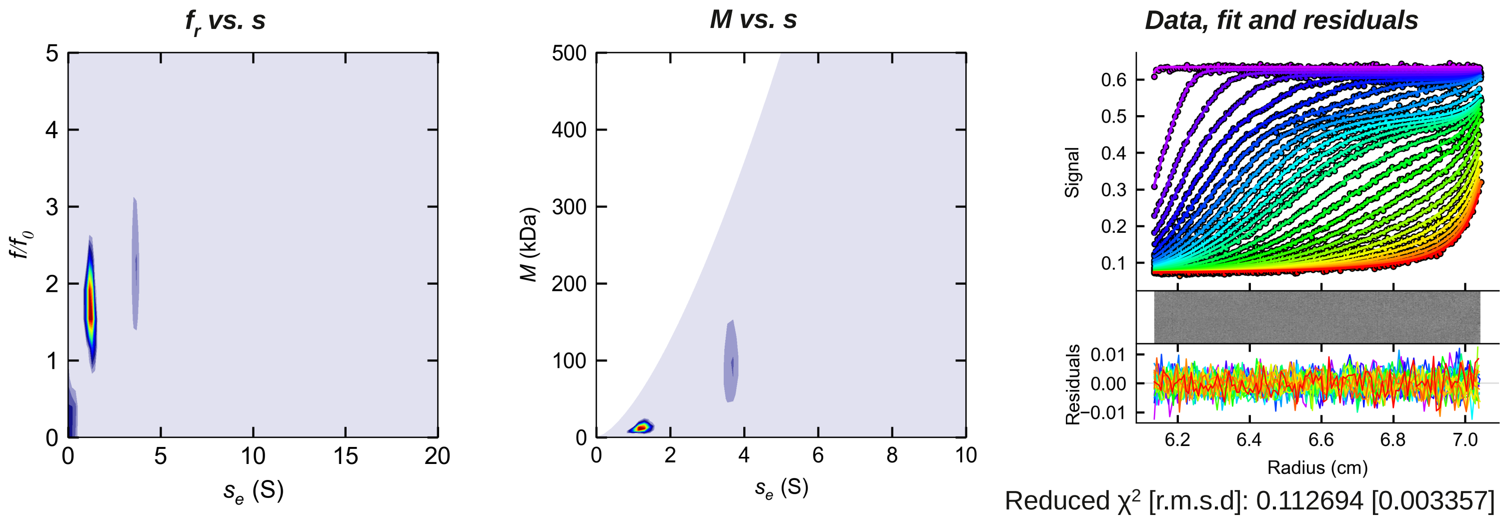

Reduced χ^2 [r.m.s.d]: 0.112694 [0.003357]

Species Analysis (42000 rpm, λ = 294 nm, exp. # 1)

Parameter	Value	95% confidence interval
Species 1		
**Sedimentation coefficient s_e (S)**	1.229725	1.2078 - 1.2518
**Diffusion coefficient D_e ($\cdot 10^{-7}$ cm²/s)**	7.837109	7.4467 - 8.2514
Species 2		
**Sedimentation coefficient s_e (S)**	3.579254	3.4808 - 3.6784
**Diffusion coefficient D_e ($\cdot 10^{-7}$ cm²/s)**	3.316965	2.1005 - 5.0301
**Fraction (signal)**	0.204758	0.1884 - 0.2216
Common		
**Total concentration (signal)**	0.2623926	0.2545 - 0.2705
**Meniscus position (cm)**	6.113078	6.1073 - 6.1188
**Bottom position (cm)**	7.227485	7.2230 - 7.2323
**Reduced χ2 of fit [rmsd]**	0.1228484	
**R.m.s.d. of fit**	0.003504974	

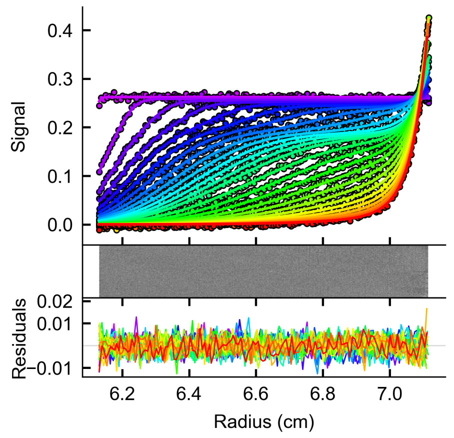

Reduced χ^2 [r.m.s.d]: 0.122848 [0.003505]

Species Analysis (42000 rpm, λ = 291 nm, exp. # 2)

Parameter	Value	95% confidence interval
Species 1		
**Sedimentation coefficient s_e (S)**	1.215424	1.2044 - 1.2265
**Diffusion coefficient D_e ($\cdot 10^{-7}$ cm²/s)**	8.173451	7.8048 - 8.4944
Species 2		
**Sedimentation coefficient s_e (S)**	3.590108	3.5299 - 3.6475
**Diffusion coefficient D_e ($\cdot 10^{-7}$ cm²/s)**	3.140034	2.3970 - 4.0048
**Fraction (signal)**	0.168034	0.1588 - 0.1769
Common		
**Total concentration (signal)**	0.5605898	0.1588 - 0.1769
**Meniscus position (cm)**	6.117462	6.1147 - 6.1205
**Bottom position (cm)**	7.234521	7.2271 - 7.2411
**Reduced χ2 of fit [rmsd]**	0.1260963	
**R.m.s.d. of fit**	0.003551004	

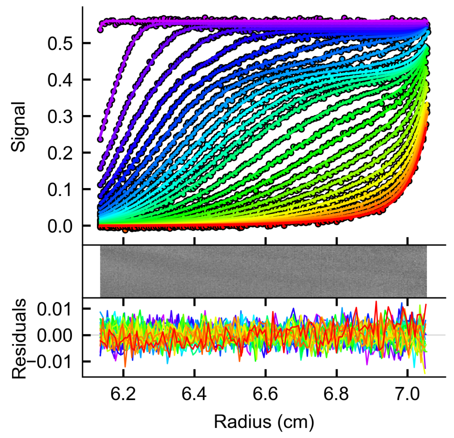

Reduced χ^2 [r.m.s.d]: 0.126096 [0.003551]

Fig. S2B

32 µM ssRNA monitored by absorbance optics

42000 rpm, λ = 294 nm

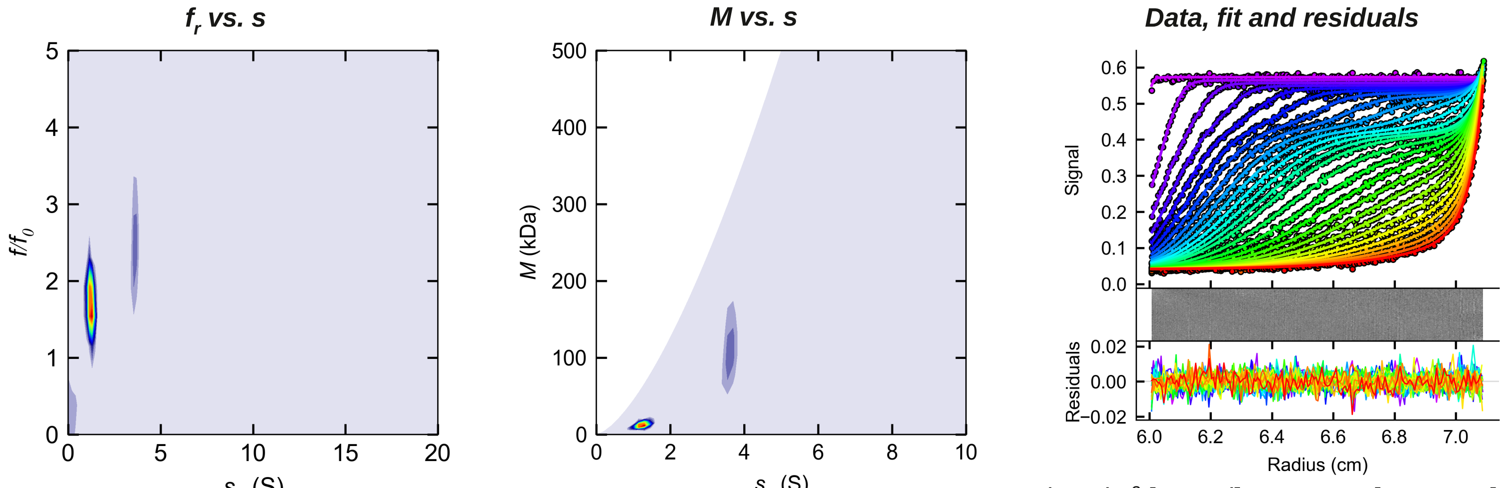

Reduced χ^2 [r.m.s.d]: 0.191756 [0.004379]

42000 rpm, λ = 291 nm

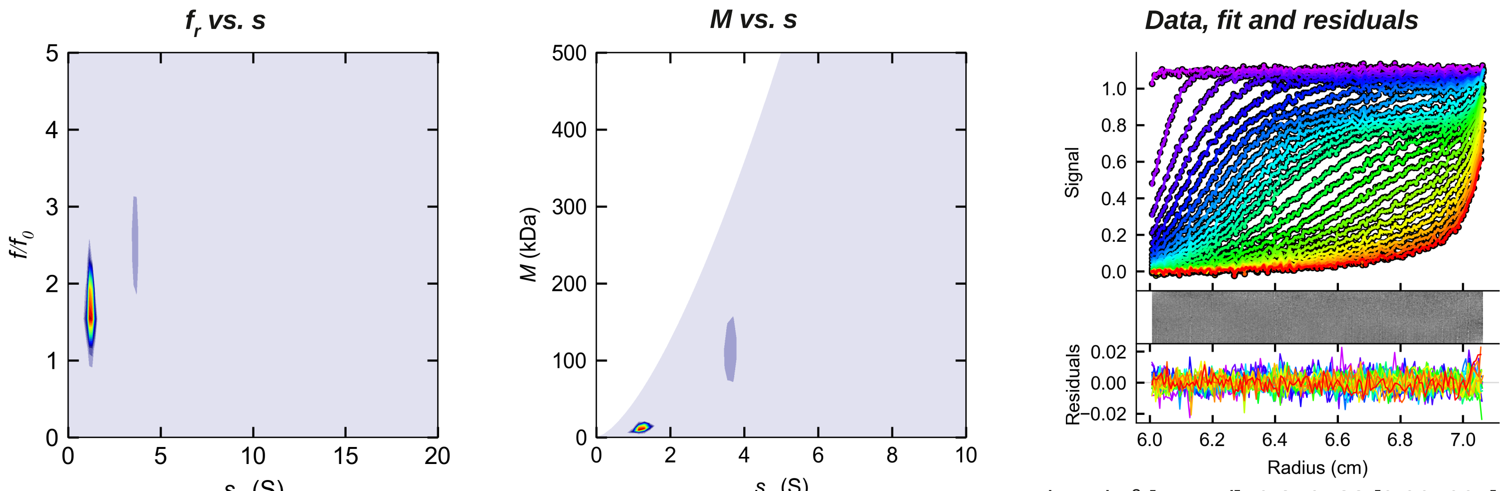

Reduced χ^2 [r.m.s.d]: 0.259183 [0.005091]

Species Analysis (42000 rpm, λ = 294 nm, exp. # 1)

Parameter	Value	95% confidence interval
	Species 1	
**Sedimentation coefficient s_e (S)**	1.232830	1.2201 - 1.2468
**Diffusion coefficient D_e ($\cdot 10^{-7}$ cm²/s)**	8.121437	7.7671 - 8.5080
	Species 2	
**Sedimentation coefficient s_e (S)**	3.585519	3.5308 - 3.6467
**Diffusion coefficient D_e ($\cdot 10^{-7}$ cm²/s)**	3.231844	2.4451 - 4.1974
**Fraction (signal)**	0.202133	0.1919 - 0.2126
	Common	
**Total concentration (signal)**	0.5306498	0.5205 - 0.5417
**Meniscus position (cm)**	5.986057	5.9823 - 5.9896
**Bottom position (cm)**	7.236567	7.2309 - 7.2428
**Reduced χ2 of fit [rmsd]**	0.1983076	
**R.m.s.d. of fit**	0.004453174	

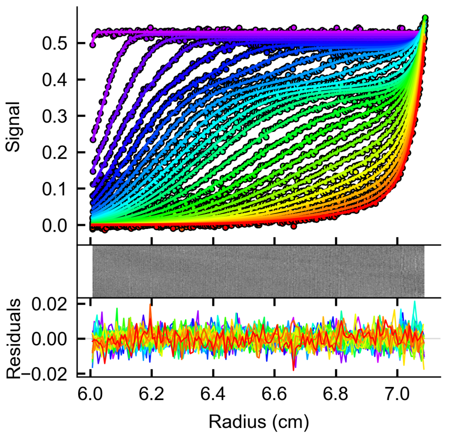

Reduced χ^2 [r.m.s.d]: 0.198308 [0.004453]

Species Analysis (42000 rpm, λ = 291 nm, exp. # 2)

Parameter	Value	95% confidence interval
	Species 1	
**Sedimentation coefficient s_e (S)**	1.220184	1.2127 - 1.2280
**Diffusion coefficient D_e ($\cdot 10^{-7}$ cm²/s)**	7.938753	7.7172 - 8.1994
	Species 2	
**Sedimentation coefficient s_e (S)**	3.596446	3.5560 - 3.6386
**Diffusion coefficient D_e ($\cdot 10^{-7}$ cm²/s)**	3.366156	2.7780 - 4.0454
**Fraction (signal)**	0.170676	0.1640 - 0.1770
	Common	
**Total concentration (signal)**	1.088870	1.0756 - 1.1033
**Meniscus position (cm)**	5.989551	5.9874 - 5.9917
**Bottom position (cm)**	7.235867	7.2314 - 7.2412
**Reduced χ2 of fit [rmsd]**	0.2852372	
**R.m.s.d. of fit**	0.005340760	

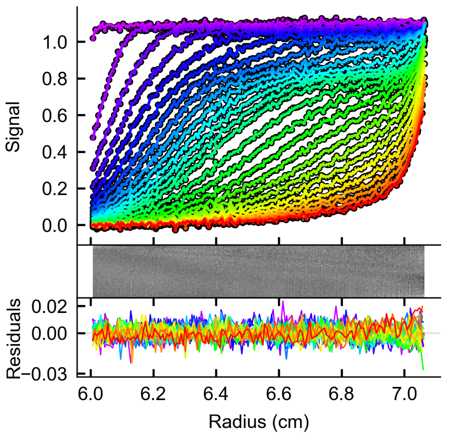

Reduced χ^2 [r.m.s.d]: 0.285237 [0.005341]

Fig. S2C

64 µM ssRNA monitored by absorbance optics

42000 rpm, λ = 294 nm

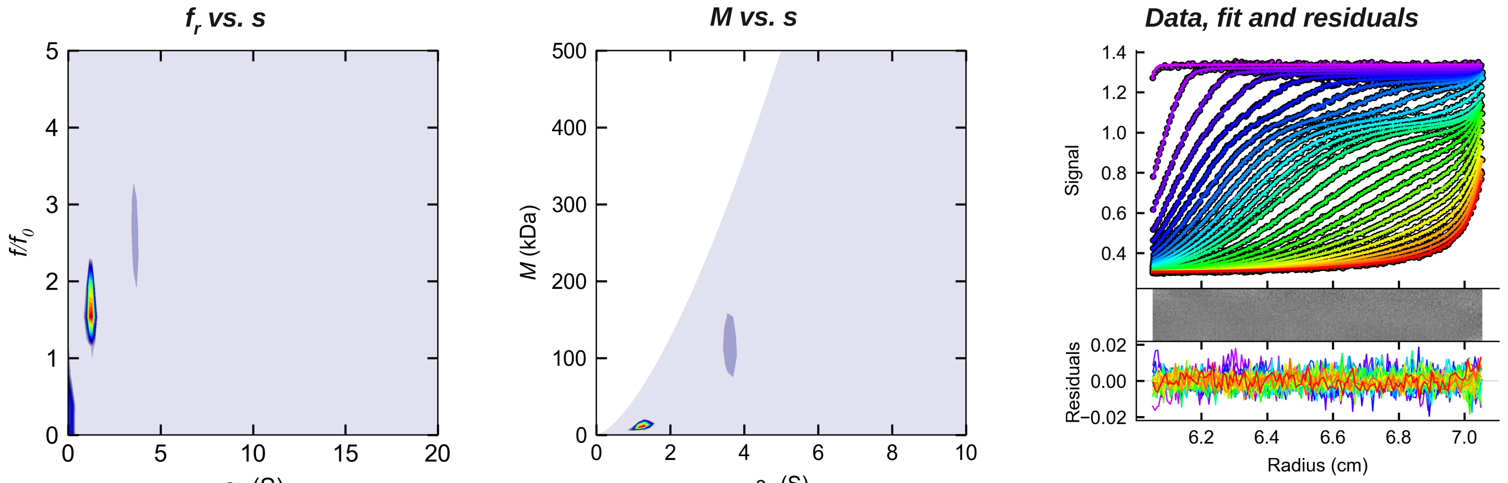

Reduced χ² [r.m.s.d]: 0.211508 [0.004599]

42000 rpm, λ = 291 nm

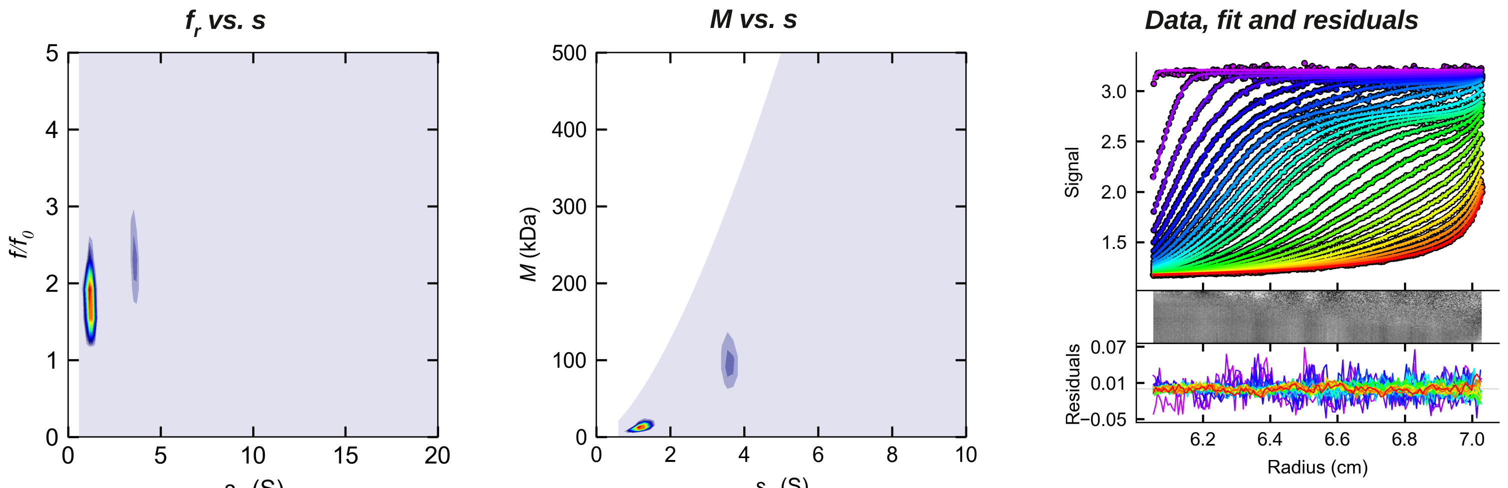

Reduced χ² [r.m.s.d]: 1.01949 [0.010097]

Species Analysis (42000 rpm, λ = 294 nm, exp. # 1)

Parameter	Value	95% confidence interval
	Species 1	
**Sedimentation coefficient s_e (S)**	1.239898	1.2324 - 1.2474
**Diffusion coefficient D_e ($\cdot 10^{-7}$ cm²/s)**	8.170592	7.9399 - 8.4006
	Species 2	
**Sedimentation coefficient s_e (S)**	3.572412	3.5362 - 3.6070
**Diffusion coefficient D_e ($\cdot 10^{-7}$ cm²/s)**	3.355237	2.8711 - 3.9210
**Fraction (signal)**	0.198935	0.1930 - 0.2052
	Common	
**Total concentration (signal)**	1.024325	1.0124 - 1.0365
**Meniscus position (cm)**	6.025673	6.0235 - 6.0279
**Bottom position (cm)**	7.241323	7.2362 - 7.2464
**Reduced χ2 of fit [rmsd]**	0.2163596	
**R.m.s.d. of fit**	0.004651447	

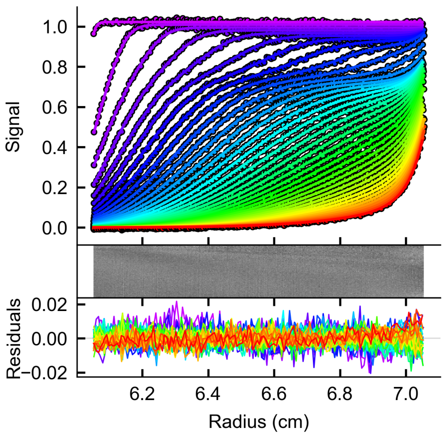

Reduced χ² [r.m.s.d]: 0.21630 [0.004651]

Species Analysis (42000 rpm, λ 291 nm, exp. # 2)

Parameter	Value	95% confidence interval
	Species 1	
**Sedimentation coefficient s_e (S)**	1.201514	1.1918 - 1.2113
**Diffusion coefficient D_e ($\cdot 10^{-7}$ cm²/s)**	8.107909	7.7838 - 8.4409
	Species 2	
**Sedimentation coefficient s_e (S)**	3.534074	3.4728 - 3.5943
**Diffusion coefficient D_e ($\cdot 10^{-7}$ cm²/s)**	2.847448	2.1050 - 3.7689
**Fraction (signal)**	0.132244	0.1238 - 0.1408
	Common	
**Total concentration (signal)**	2.030704	1.9974 - 2.0654
**Meniscus position (cm)**	6.027494	6.0247 - 6.0302
**Bottom position (cm)**	7.245158	7.2363 - 7.2544
**Reduced χ2 of fit [rmsd]**	1.393352	
**R.m.s.d. of fit**	0.011804033	

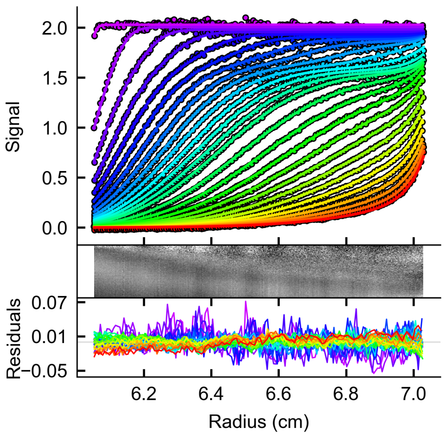

Reduced χ² [r.m.s.d]: 1.393352 [0.011804]

Fig. S2D

Section 2:

Characterization of
Human PCBP2, PRRSV nsp1-β and PRRSV 34 nt ssRNA
Complex by Sedimentation Velocity

Complex of human PCBP2, PRRSV nsp1-β and PRRSV 34 nt ssRNA

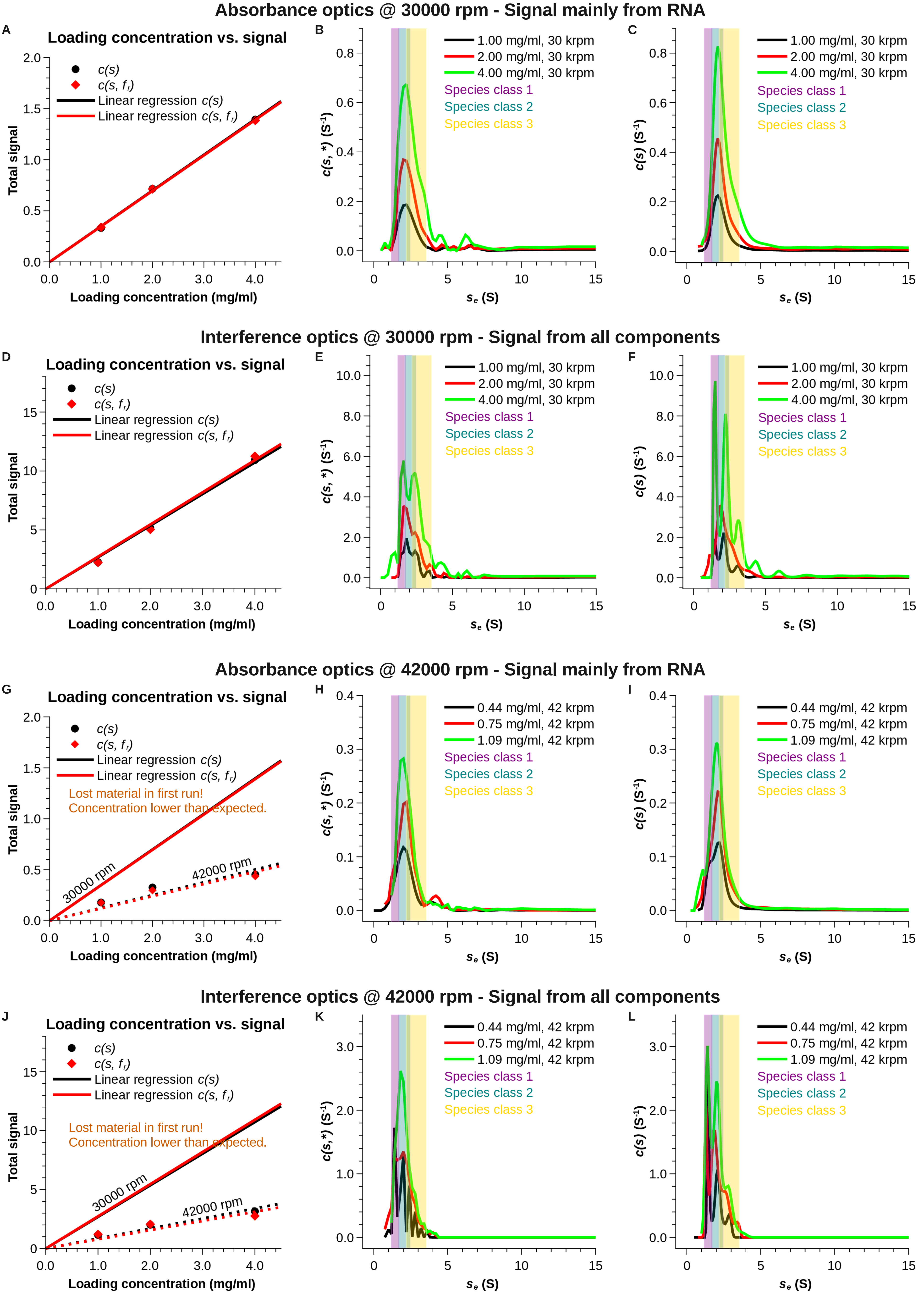

Fig. S3

Species classes

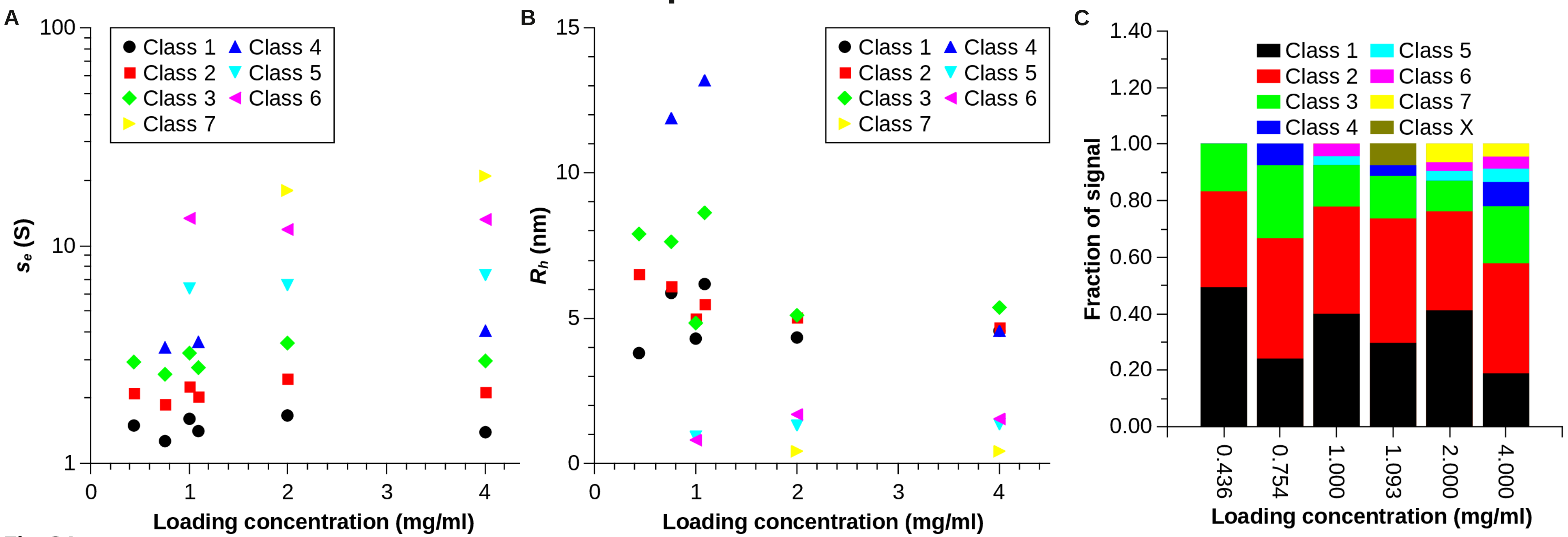

Fig. S4

Results

Species class 1	
Sedimentation coefficient $s^0_{20°C,w}$	2.02 [1.61 - 2.43] S
Diffusion coefficient $D^0_{20°C,w}$	4.52 [2.76 - 6.28] ·10^{-7} cm²/s
Hydrodynamic radius R_h^0	4.74 [3.41 - 7.74] nm
Species class 2	
Sedimentation coefficient $s^0_{20°C,w}$	2.84 [2.28 - 3.41] S
Diffusion coefficient $D^0_{20°C,w}$	3.51 [2.87 - 4.15] ·10^{-7} cm²/s
Hydrodynamic radius R_h^0	6.11 ± [5.17 - 7.46] nm
Species class 3	
Sedimentation coefficient $s^0_{20°C,w}$	3.96 [2.97 - 4.94] S
Diffusion coefficient $D^0_{20°C,w}$	2.92 [1.37 - 4.47] ·10^{-7} cm²/s
Hydrodynamic radius R_h^0	7.34 [4.79 - 15.61] nm

Table S5

Results

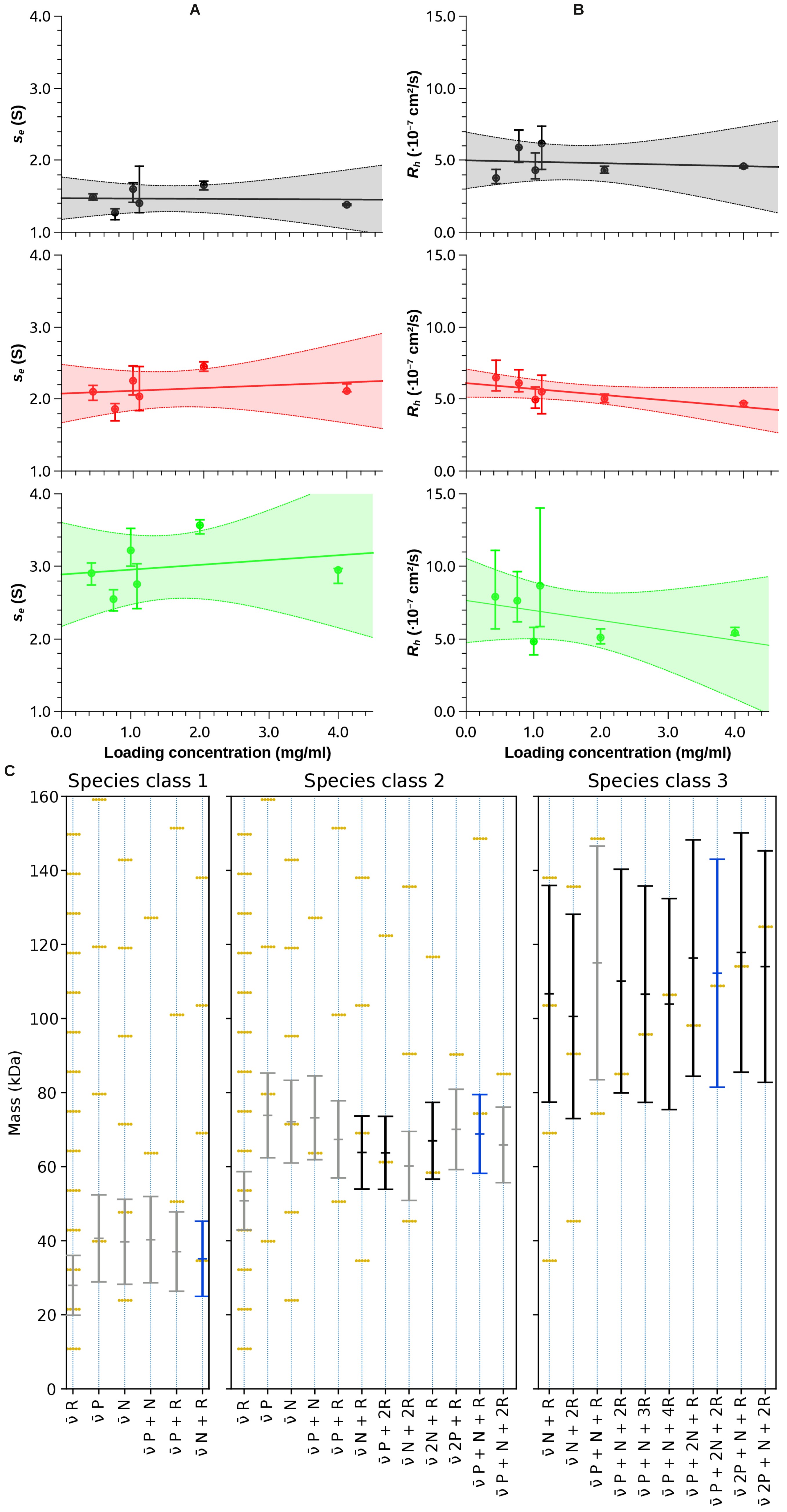

P: PCBP2, N: nsp1β, R: 34nt ssRNA

Fig. S5

Complex of human PCBP2, PRRSV nsp1-β and PRRSV 34 nt ssRNA

Absorbance optics @ 30000 rpm - loading concentration 1.0 mg/ml

c(s, f_r) analysis

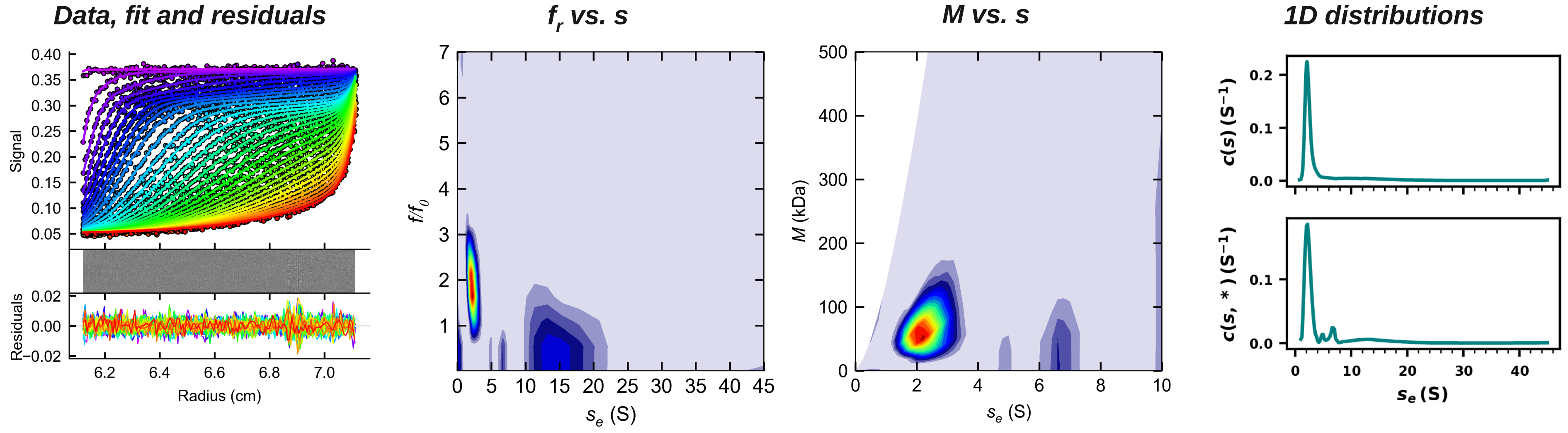

Reduced χ^2 [r.m.s.d]: [0.003905]

Absorbance optics @ 42000 rpm - actual concentration 0.44 mg/ml

c(s, f_r) analysis

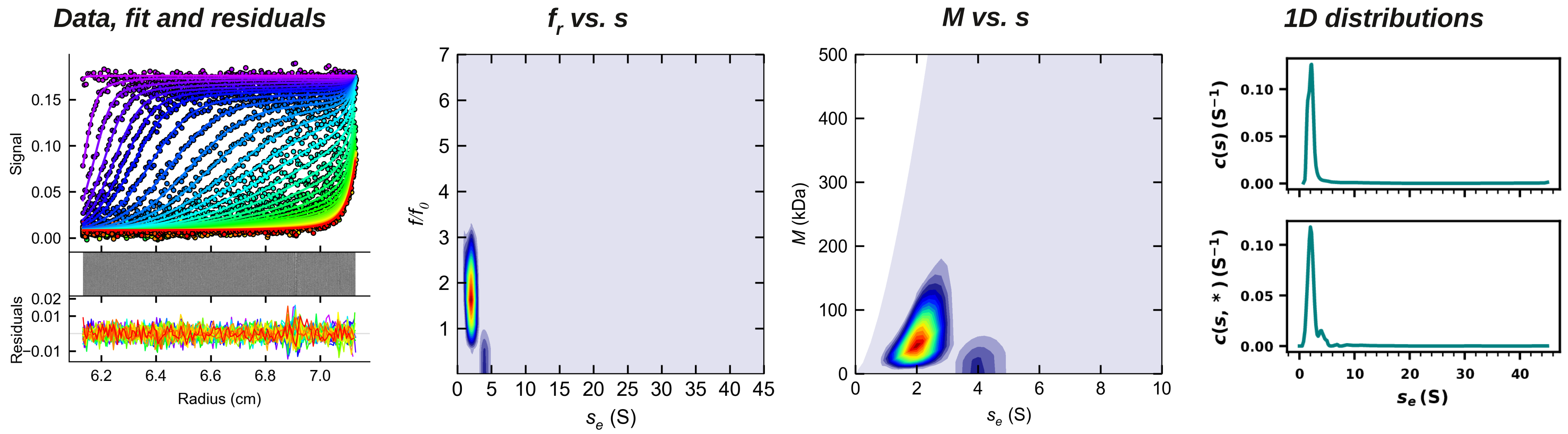

Reduced χ^2 [r.m.s.d]: [0.003341]

Fig. S6A

Complex of human PCBP2, PRRSV nsp1-β and PRRSV 34 nt ssRNA

Interference optics @ 30000 rpm - loading concentration 1.0 mg/ml

c(s, f_r) analysis

Data, fit and residuals | ***f_r vs. s*** | ***M vs. s*** | ***1D distributions***

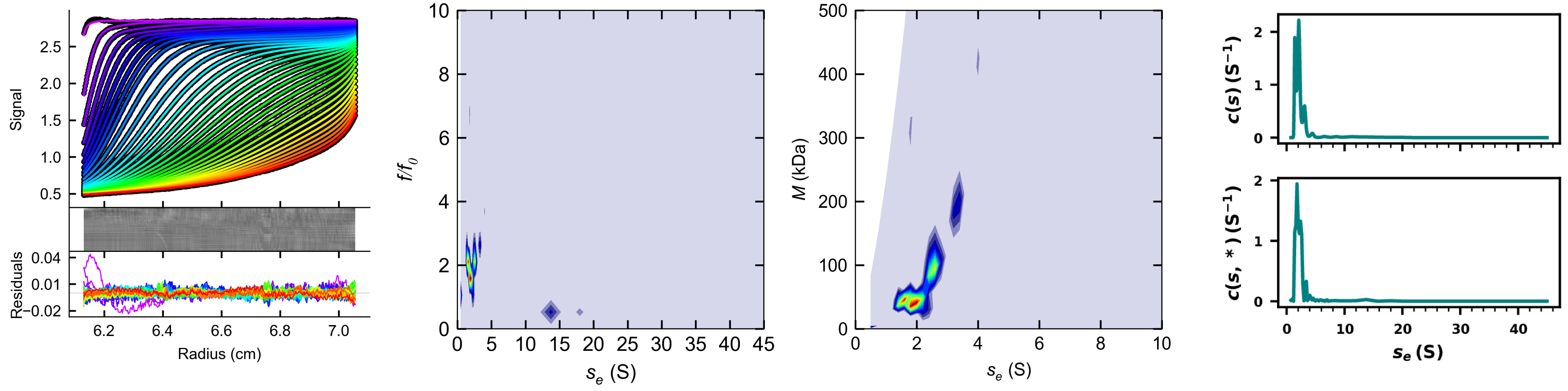

Reduced χ² [r.m.s.d]: [0.003815]

Interference optics @ 42000 rpm - actual concentration 0.44 mg/ml

c(s, f_r) analysis

Data, fit and residuals | ***f_r vs. s*** | ***M vs. s*** | ***1D distributions***

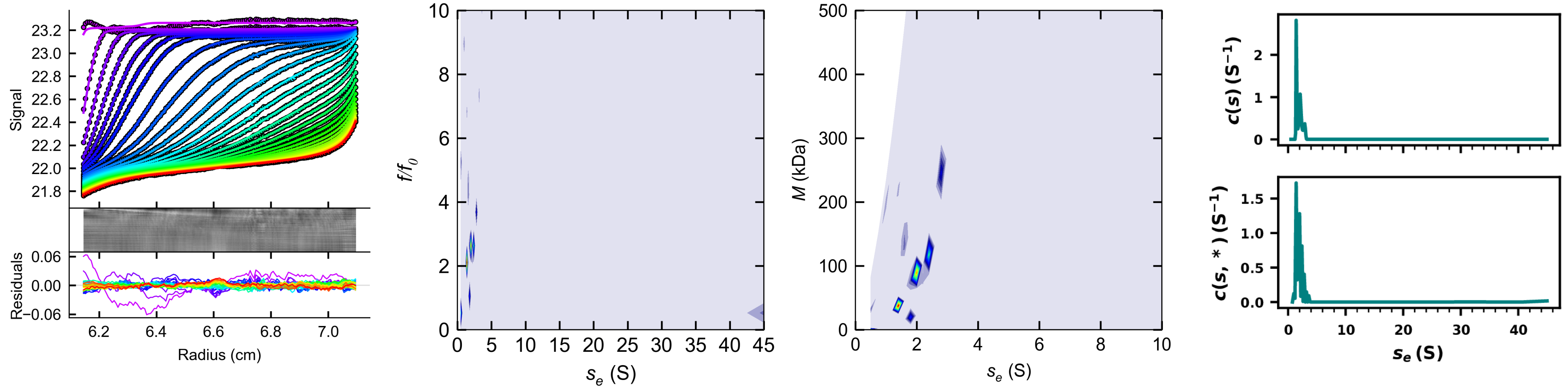

Reduced χ² [r.m.s.d]: [0.006460]

Fig. S6B

Complex of human PCBP2, PRRSV nsp1-β and PRRSV 34 nt ssRNA

Species Analysis (1.00 mg/ml, 30000 rpm, i. o.)

Parameter	Value	95% confidence interval
Species # 1 (class 1)		
**Sedimentation coefficient $s_{20°C,w}$ (S)**	2.185538	1.9320 - 2.3175
**Molecular mass[1] *M* (Da)**	39429.258626	31975.1983 - 43851.1943
**Concentration (signal)**	0.889672	
Species # 2 (class 2)		
**Sedimentation coefficient $s_{20°C,w}$ (S)**	3.086904	2.8131 - 3.3679
**Molecular mass[1] *M* (Da)**	64508.620238	56946.9871 - 72146.4835
**Concentration (signal)**	0.848381	
Species # 3 (class 3)		
**Sedimentation coefficient $s_{20°C,w}$ (S)**	4.413055	4.1166 - 4.8238
**Molecular mass[1] *M* (Da)**	89133.977232	75536.5637 - 117267.7025
**Concentration (signal)**	0.327668	
Species # 4 (class 5)		
**Sedimentation coefficient $s_{20°C,w}$ (S)**	8.680858	6.5740 - 11.0911
**Molecular mass[1] *M* (Da)**	33004.982556	27014.9568 - 41589.0286
**Concentration (signal)**	0.0689382	
Species # 5 (class 6)		
**Sedimentation coefficient $s_{20°C,w}$ (S)**	18.269621	17.2184 - 19.6377
**Molecular mass[1] *M* (Da)**	62045.164082	50792.5477 - 83330.0656
**Concentration (signal)**	0.101118	
Common		
**Meniscus position (cm)**	6.107007	6.1061 - 6.1081
**Bottom position (cm)**	7.216139	7.2101 - 7.2245
**Buffer signal[2] frictional ratio f_r**	2.382192	1.8745 - 3.3470
**Reduced χ2 of fit [rmsd]**	0.1871007	
**R.m.s.d. of fit**	0.004326	

[1]Substitute $\bar{v}$ = 0.73 cm^3/g
[2]Continuous *c(s)* from 0.0 - 0.8 S

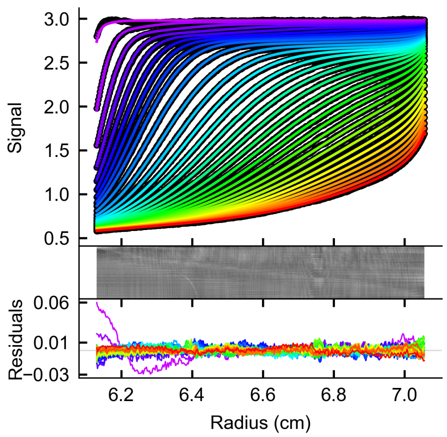

Reduced χ^2 [r.m.s.d]: 0.187101 [0.004326]

Fig. S6C

Complex of human PCBP2, PRRSV nsp1-β and PRRSV 34 nt ssRNA

Species Analysis (0.44 mg/ml, 42000 rpm, i. o.)

Parameter	Value	95% confidence interval
Species # 1 (class 1)		
**Sedimentation coefficient $s_{20°C, w}$ (S)**	2.045761	1.9890 - 2.0955
**Molecular mass[1] *M* (Da)**	32468.185230	28140.1889 - 35903.1468
**Concentration (signal)**	0.564412	
Species # 2 (class 2)		
**Sedimentation coefficient $s_{20°C, w}$ (S)**	2.874757	2.7103 - 2.9979
**Molecular mass[1] *M* (Da)**	78493.617851	67236.8428 - 93921.0463
**Concentration (signal)**	0.388871	
Species # 3 (class 3)		
**Sedimentation coefficient $s_{20°C, w}$ (S)**	3.976393	3.7610 - 4.1698
**Molecular mass[1] *M* (Da)**	131871.067920	94586.0501 - 201092.7657
**Concentration (signal)**	0.193644	
Common		
**Meniscus position (cm)**	6.107400	6.1056 - 6.1093
**Bottom position (cm)**	7.201615	7.1931 - 7.2121
**Buffer signal[2] frictional ratio f_r**	1.481410	1.2663 - 1.9532
**Reduced χ2 of fit [rmsd]**	0.5931672	
**R.m.s.d. of fit**	0.007702	

[1]Substitute $\bar{v}$ = 0.73 cm^3/g
[2]Continuous *c(s)* from 0.0 - 0.8 S

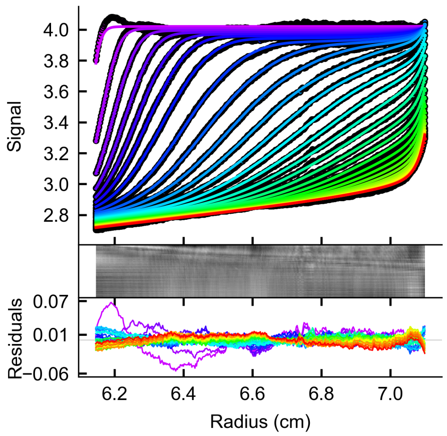

Reduced χ^2 [r.m.s.d]: 0.593167 [0.007702]

Fig. S6D

Complex of human PCBP2, PRRSV nsp1-β and PRRSV 34 nt ssRNA

Absorbance optics @ 30000 rpm - loading concentration 2.0 mg/ml

c(s, f_r) analysis

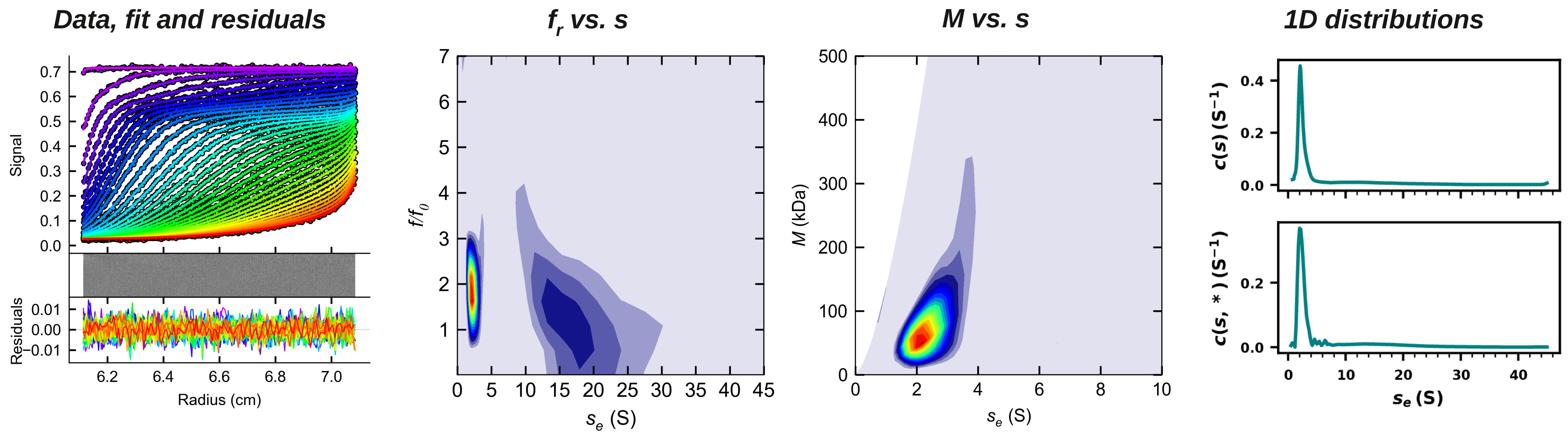

Reduced χ^2 [r.m.s.d]: [0.003730]

Absorbance optics @ 42000 rpm - actual concentration 0.75 mg/ml

c(s, f_r) analysis

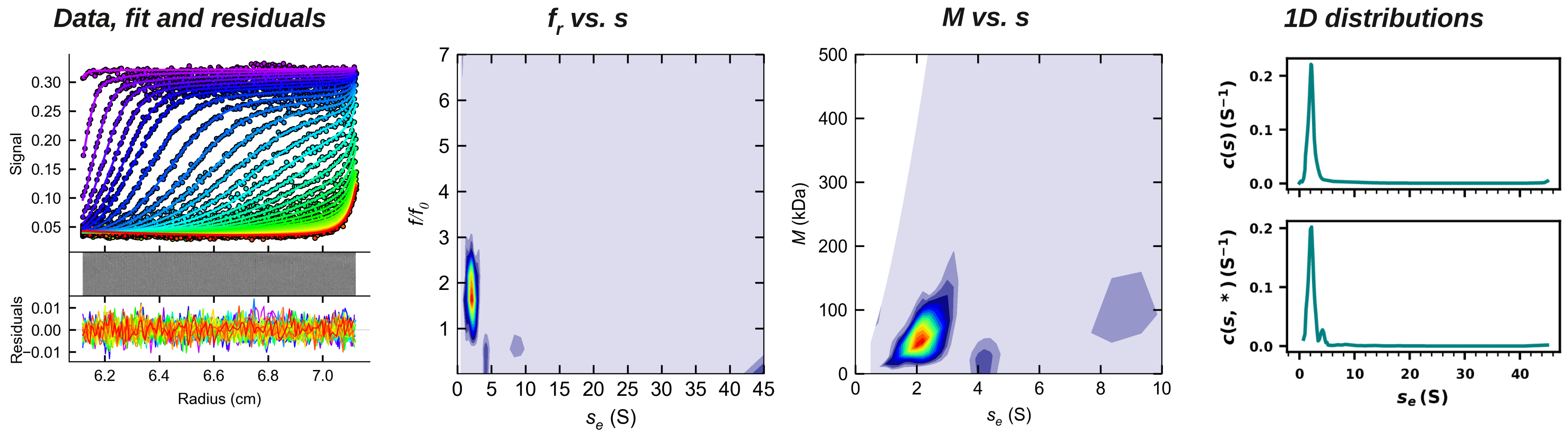

Reduced χ^2 [r.m.s.d]: [0.003382]

Fig. S6E

Complex of human PCBP2, PRRSV nsp1-β and PRRSV 34 nt ssRNA

Interference optics @ 30000 rpm - loading concentration 2.0 mg/ml

c(s, f_r) analysis

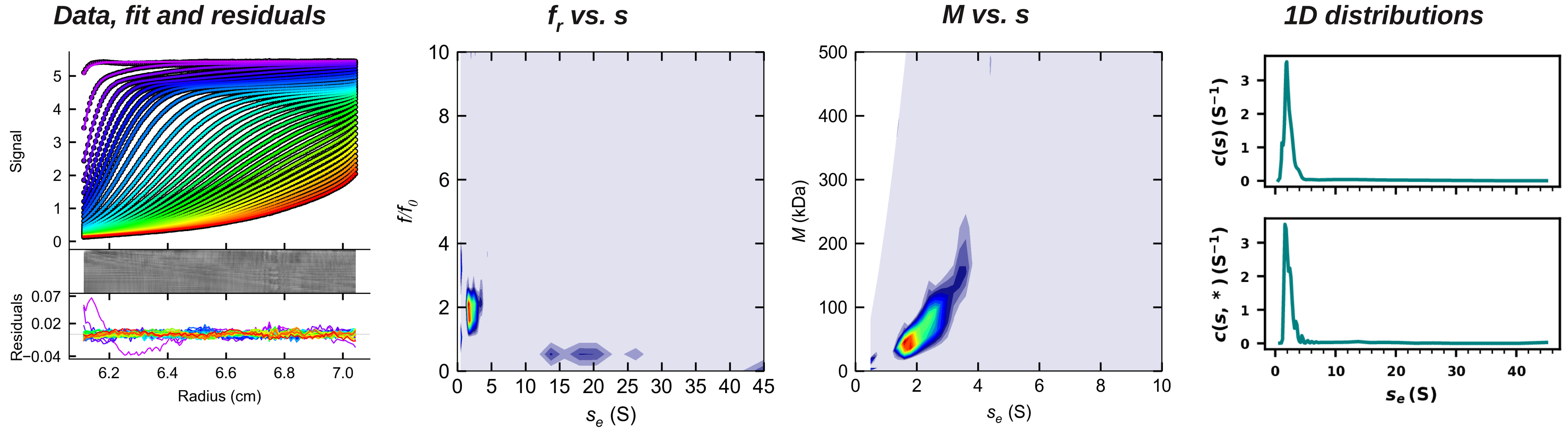

Reduced χ^2 [r.m.s.d]: [0.005117]

Interference optics @ 42000 rpm - actual concentration 0.75 mg/ml

c(s, f_r) analysis

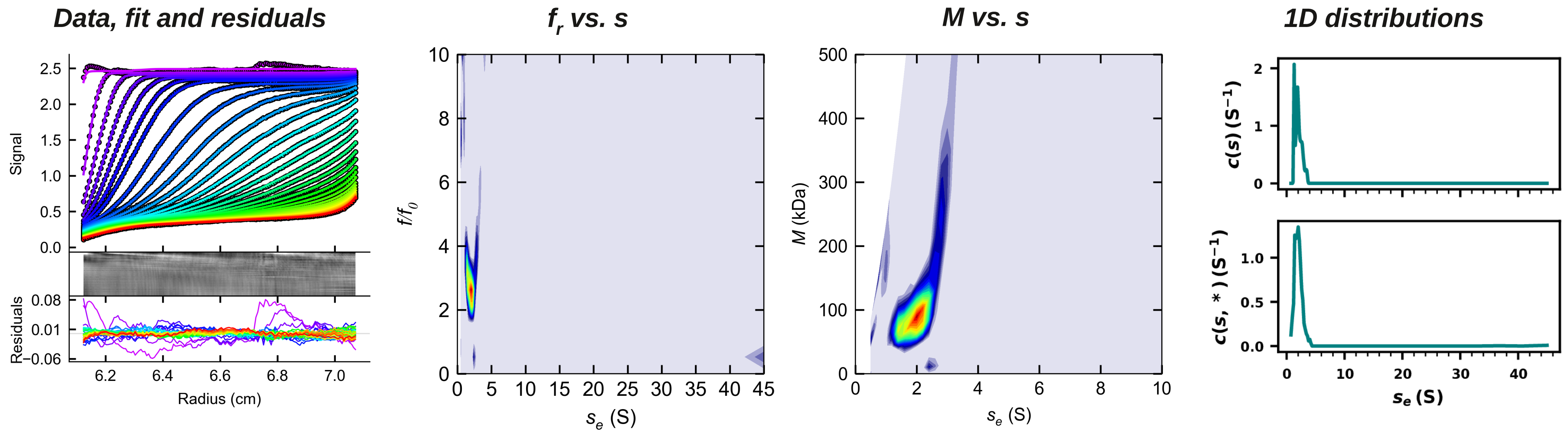

Reduced χ^2 [r.m.s.d]: [0.009482]

Fig. S6F

Complex of human PCBP2, PRRSV nsp1-β and PRRSV 34 nt ssRNA

Species Analysis (2.00 mg/ml, 30000 rpm, i. o.)

**Parameter**	**Value**	**95% confidence interval**
Species # 1 (class 1)		
**Sedimentation coefficient $s_{20°C, w}$ (S)**	2.259298	2.1789 - 2.3345
**Molecular mass[1] *M* (Da)**	40911.383621	39015.7993 - 42569.8757
**Concentration (signal)**	2.10665	
Species # 2 (class 2)		
**Sedimentation coefficient $s_{20°C, w}$ (S)**	3.362426	3.2647 - 3.4499
**Molecular mass[1] *M* (Da)**	70607.408753	66954.0626 - 74408.5887
**Concentration (signal)**	1.80079	
Species # 3 (class 3)		
**Sedimentation coefficient $s_{20°C, w}$ (S)**	4.879189	4.7237 - 4.9818
**Molecular mass[1] *M* (Da)**	104525.361816	94748.6103 - 115580.2252
**Concentration (signal)**	0.557564	
Species # 4 (class 5)		
**Sedimentation coefficient $s_{20°C, w}$ (S)**	9.007732	7.8572 - 10.1107
**Molecular mass[1] *M* (Da)**	49129.251609	38405.4058 - 69148.1903
**Concentration (signal)**	0.179173	
Species # 5 (class 6)		
**Sedimentation coefficient $s_{20°C, w}$ (S)**	16.172510	15.2395 - 17.1308
**Molecular mass[1] *M* (Da)**	114826.491328	86165.0080 - 173746.5190
**Concentration (signal)**	0.15973	
Species # 6 (class 7)		
**Sedimentation coefficient $s_{20°C, w}$ (S)**	24.574698	23.3895 - 25.9063
**Molecular mass[1] *M* (Da)**	42732.956296	38685.9354 - 49078.2030
**Concentration (signal)**	0.337733	
Common		
**Meniscus position (cm)**	6.078588	6.0778 - 6.0790
**Bottom position (cm)**	7.233989	7.2288 - 7.2399
**Buffer signal[2] frictional ratio f_r**	3.336644	2.8806 - 3.9916
**Reduced χ2 of fit [rmsd]**	0.3392895	
**R.m.s.d. of fit**	0.005825	

[1]Substitute $\bar{v}$ = 0.73 cm^3/g
[2]Continuous *c(s)* from 0.0 - 0.8 S

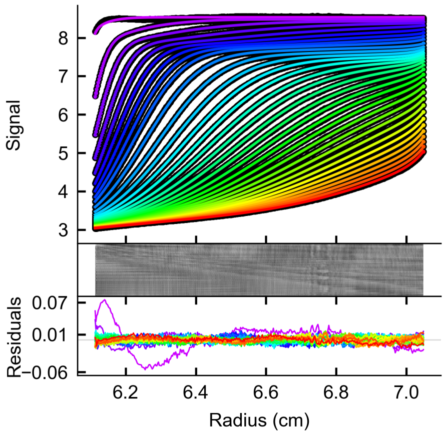

Reduced χ^2 [r.m.s.d]: 0.339290 [0.005825]

Fig. S6G

Complex of human PCBP2, PRRSV nsp1-β and PRRSV 34 nt ssRNA

Species Analysis (0.75 mg/ml, 42000 rpm, i. o.)

**Parameter**	**Value**	**95% confidence interval**
Species # 1 (class 1)		
**Sedimentation coefficient $s_{20°C,w}$ (S)**	1.737800	1.6152 - 1.8163
**Molecular mass[1] *M* (Da)**	42836.913056	36022.7457 - 53638.9211
**Concentration (signal)**	0.488908	
Species # 2 (class 2)		
**Sedimentation coefficient $s_{20°C,w}$ (S)**	2.545311	2.3295 - 2.6511
**Molecular mass[1] *M* (Da)**	65269.106310	58823.6621 - 72195.2814
**Concentration (signal)**	0.869682	
Species # 3 (class 3)		
**Sedimentation coefficient $s_{20°C,w}$ (S)**	3.497679	3.2748 - 3.6633
**Molecular mass[1] *M* (Da)**	112116.879536	90117.1719 - 141988.8079
**Concentration (signal)**	0.526914	
Species # 4 (class 4)		
**Sedimentation coefficient $s_{20°C,w}$ (S)**	4.655214	4.3692 - 4.8847
**Molecular mass[1] *M* (Da)**	232327.712281	148281.3925 - 426686.8809
**Concentration (signal)**	0.156663	
Common		
**Meniscus position (cm)**	6.093045	6.0911 - 6.0950
**Bottom position (cm)**	7.175154	7.1615 - 7.1904
**Buffer signal[2] frictional ratio f_r**	5.718135	4.3407 - 7.4711
**Reduced χ2 of fit [rmsd]**	0.9201238	
**R.m.s.d. of fit**	0.009592	

[1]Substitute $\bar{v}$ = 0.73 cm^3/g
[2]Continuous *c(s)* from 0.0 - 0.8 S

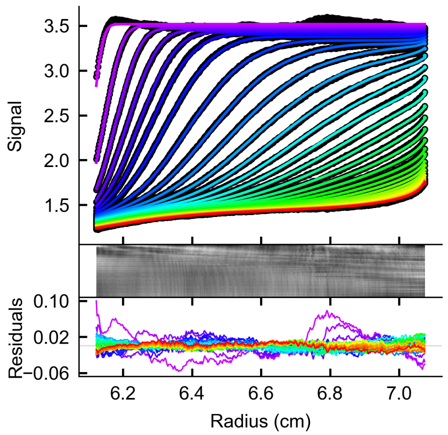

Reduced χ^2 [r.m.s.d]: 0.920124 [0.009592]

Fig. S6H

Complex of human PCBP2, PRRSV nsp1-β and PRRSV 34 nt ssRNA

Absorbance optics @ 30000 rpm - loading concentration 4.0 mg/ml

c(s, f_r) analysis

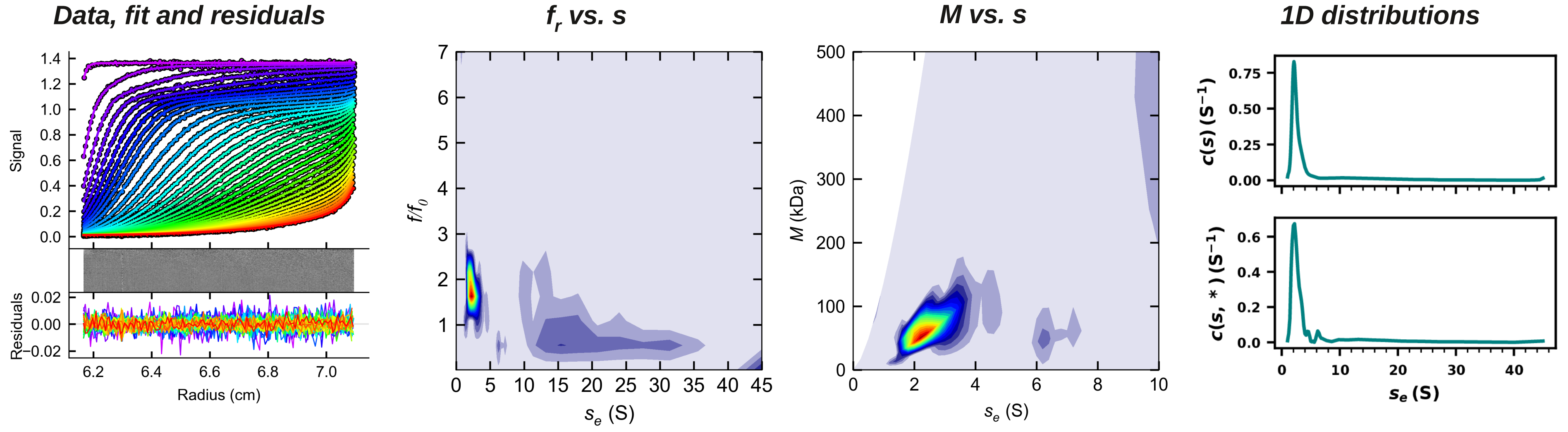

Reduced χ[2] [r.m.s.d]: [0.004508]

Absorbance optics @ 42000 rpm - actual concentration 1.09 mg/ml

c(s, f_r) analysis

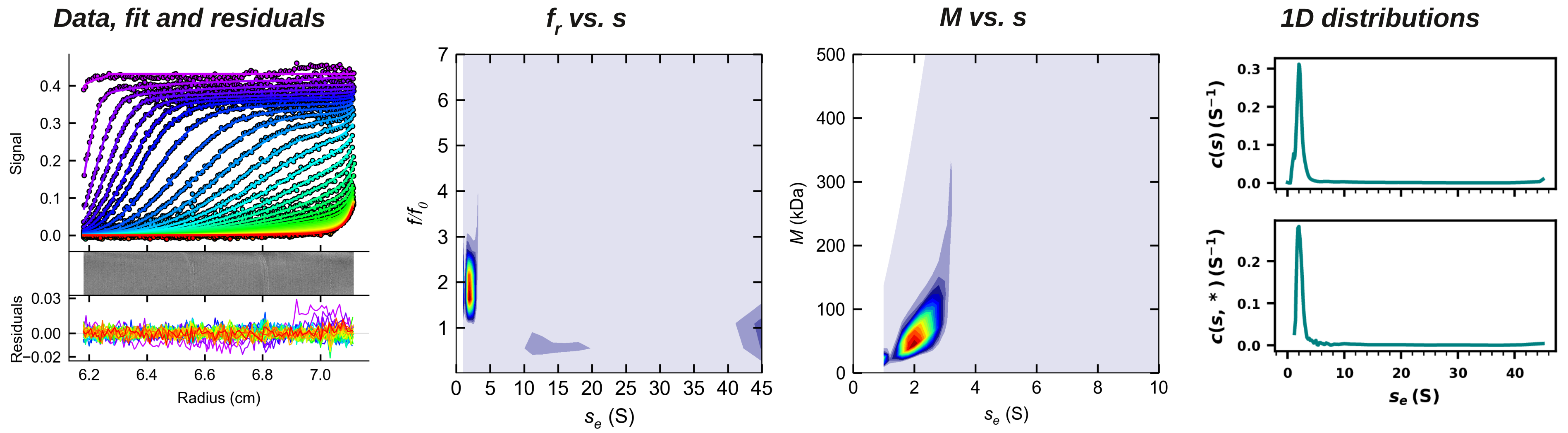

Reduced χ[2] [r.m.s.d]: [0.004322]

Fig. S6I

Complex of human PCBP2, PRRSV nsp1-β and PRRSV 34 nt ssRNA

Interference optics @ 30000 rpm - loading concentration 4.0 mg/ml

$c(s, f_r)$ analysis

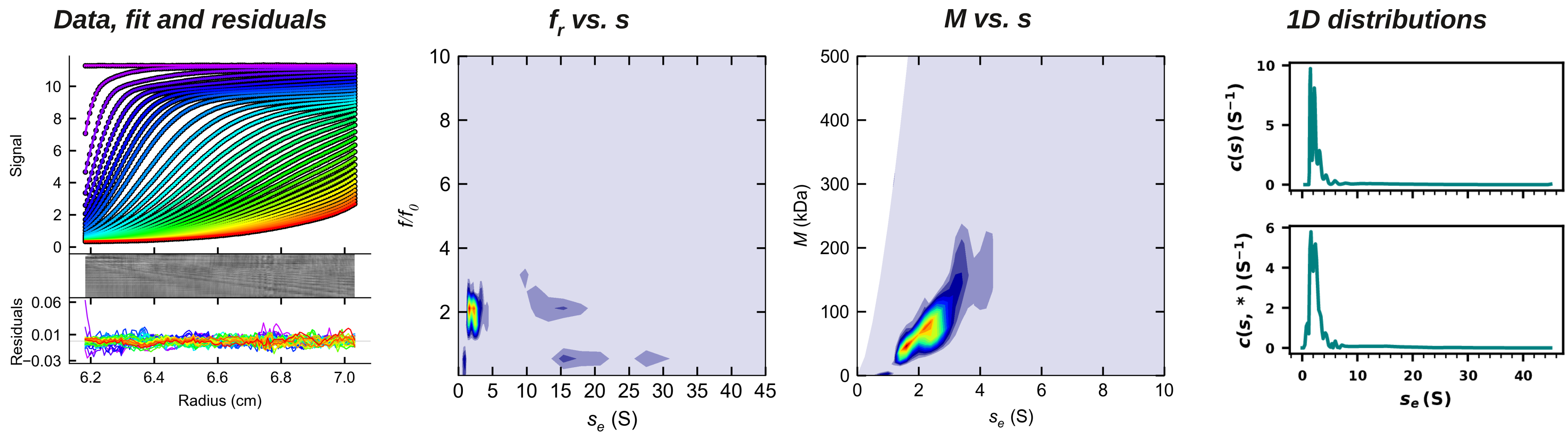

Reduced χ² [r.m.s.d]: [0.005735]

Interference optics @ 42000 rpm - actual concentration 1.09 mg/ml

$c(s, f_r)$ analysis

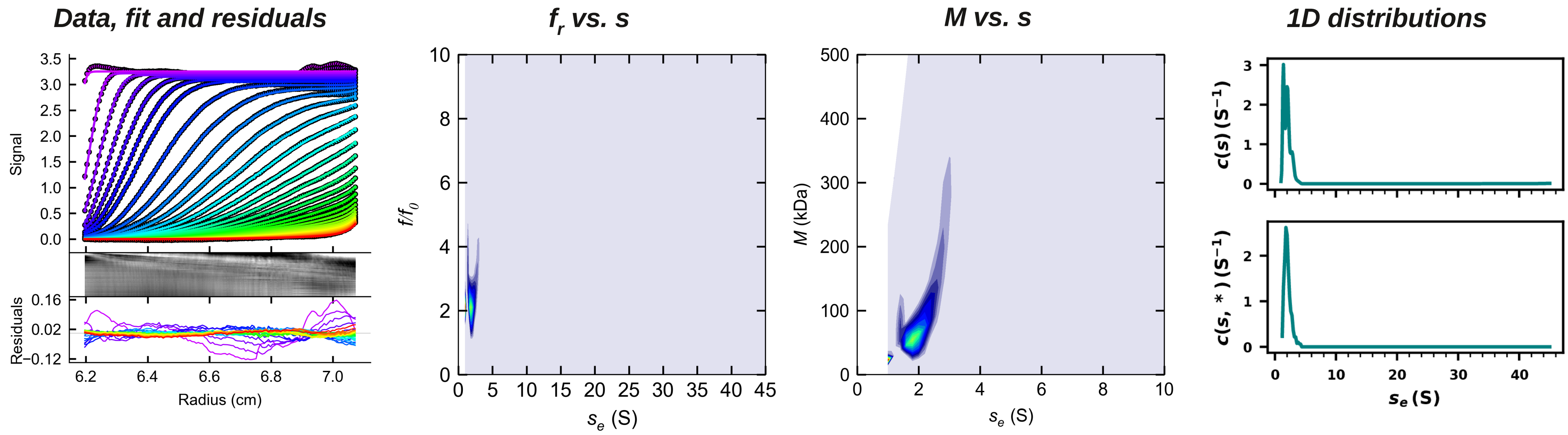

Reduced χ² [r.m.s.d]: [0.019392]

Fig. S6J

Complex of human PCBP2, PRRSV nsp1-β and PRRSV 34 nt ssRNA

Species Analysis (4.00 mg/ml, 30000 rpm, i. o.)

Parameter	Value	95% confidence interval
Species # 1 (class 1)		
**Sedimentation coefficient $s_{20°C, w}$ (S)**	1.893838	1.8767 - 1.9115
**Molecular mass[1] *M* (Da)**	36242.513823	35605.9184 - 36924.8539
**Concentration (signal)**	2.02274	
Species # 2 (class 2)		
**Sedimentation coefficient $s_{20°C, w}$ (S)**	2.895957	2.8717 - 3.0216
**Molecular mass[1] *M* (Da)**	56798.604800	56171.7866 - 57417.8843
**Concentration (signal)**	4.20148	
Species # 3 (class 3)		
**Sedimentation coefficient $s_{20°C, w}$ (S)**	4.032742	3.7914 - 4.0755
**Molecular mass[1] *M* (Da)**	91110.192293	88642.3598 - 93600.9839
**Concentration (signal)**	2.17643	
Species # 4 (class 4)		
**Sedimentation coefficient $s_{20°C, w}$ (S)**	5.558437	5.5059 - 5.7911
**Molecular mass[1] *M* (Da)**	106503.214501	97771.9841 - 113202.5506
**Concentration (signal)**	0.924506	
Species # 5 (class 5)		
**Sedimentation coefficient $s_{20°C, w}$ (S)**	9.980081	9.6854 - 10.2378
**Molecular mass[1] *M* (Da)**	55993.213184	50533.5330 - 65497.6998
**Concentration (signal)**	0.504232	
Species # 6 (class 6)		
**Sedimentation coefficient $s_{20°C, w}$ (S)**	18.163020	17.9089 - 18.4664
**Molecular mass[1] *M* (Da)**	116367.123008	102061.2320 - 138132.1828
**Concentration (signal)**	0.460625	
Species # 7 (class 7)		
**Sedimentation coefficient $s_{20°C, w}$ (S)**	28.605184	27.7262 - 30.0465
**Molecular mass[1] *M* (Da)**	50122.522087	49947.7361 - 50312.8130
**Concentration (signal)**	0.500927	
Common		
**Meniscus position (cm)**	6.154459	6.1540 - 6.1548
**Bottom position (cm)**	7.219491	7.2170 - 7.2245
**Buffer signal[2] frictional ratio f_r**	1.126869	0.9060 - 1.3521
**Reduced χ2 of fit [rmsd]**	0.5273897	
**R.m.s.d. of fit**	0.007262	

[1]Substitute $\bar{v}$ = 0.73 cm^3/g
[2]Continuous *c(s)* from 0.0 - 0.8 S

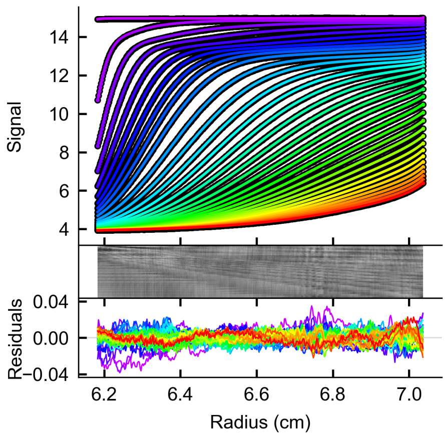

Reduced χ^2 [r.m.s.d]: 0.527390 [0.007262]

Fig. S6K

Complex of human PCBP2, PRRSV nsp1-β and PRRSV 34 nt ssRNA

Species Analysis (1.09 mg/ml, 42000 rpm, i. o.)

**Parameter**	**Value**	**95% confidence interval**
Species # 1 (class X)		
**Sedimentation coefficient $s_{20^\circ C, w}$ (S)**	1.209722	0.8561 - 1.7601
**Molecular mass[1] *M* (Da)**	30400.731201	20063.1666 - >900000
**Concentration (signal)**	0.255537	
Species # 2 (class 1)		
**Sedimentation coefficient $s_{20^\circ C, w}$ (S)**	1.922069	1.7474 - 2.6281
**Molecular mass[1] *M* (Da)**	49646.909726	42877.4221 - 61527.1873
**Concentration (signal)**	0.962814	
Species # 3 (class 2)		
**Sedimentation coefficient $s_{20^\circ C, w}$ (S)**	2.787625	2.5211 - 3.3593
**Molecular mass[1] *M* (Da)**	64072.302750	54843.6506 - 109988.7832
**Concentration (signal)**	1.43393	
Species # 4 (class 3)		
**Sedimentation coefficient $s_{20^\circ C, w}$ (S)**	3.776941	3.3105 - 4.1561
**Molecular mass[1] *M* (Da)**	136947.108751	87118.2414 - 219814.9212
**Concentration (signal)**	0.4912	
Species # 5 (class 4)		
**Sedimentation coefficient $s_{20^\circ C, w}$ (S)**	4.936165	4.2947 - 5.5977
**Molecular mass[1] *M* (Da)**	273002.737766	108529.5424 - >900000
**Concentration (signal)**	0.119144	
Common		
**Meniscus position (cm)**	6.169227	6.1674 - 6.1710
**Bottom position (cm)**	7.181567	7.1682 - 7.2017
**Buffer signal[2] frictional ratio f_r**	4.608594	2.9507 - 28.0506
**Reduced χ2 of fit [rmsd]**	3.344057	
**R.m.s.d. of fit**	0.018287	

[1]Substitute $\bar{v}$ = 0.73 cm^3/g
[2]Continuous *c(s)* from 0.0 - 0.8 S

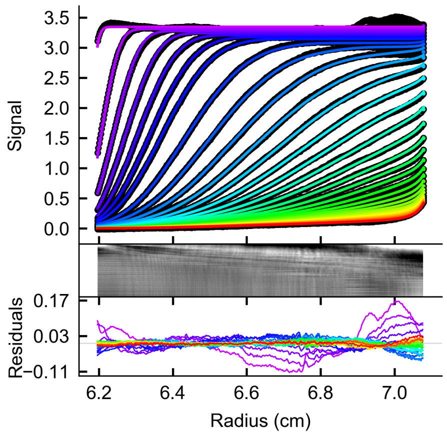

Reduced χ^2 [r.m.s.d]: 3.344057 [0.018287]

Section 3:

Characterization of

Human PCBP2, PRRSV nsp1-β and PRRSV 34 nt ssRNA

Complex by SEC-SAXS

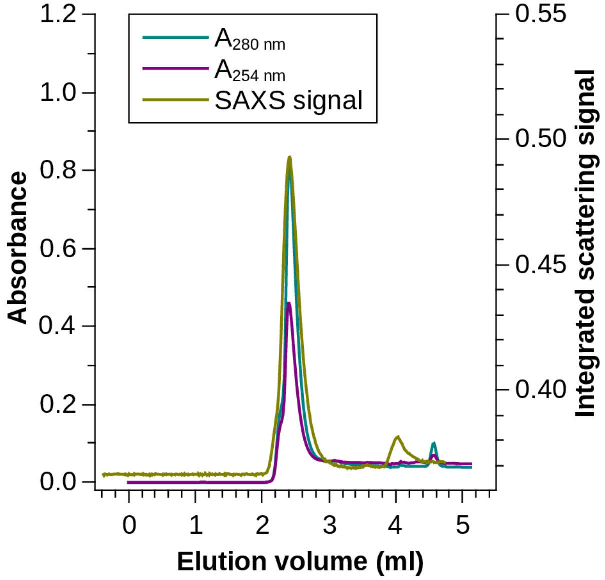

A280 nm
A254 nm
SAXS signal
Absorbance
Integrated scattering signal
Elution volume (ml)
1.2
1.0
0.8
0.6
0.4
0.2
0.0
0.55
0.50
0.45
0.40
0
1
2
3
4
5

Fig. S7
