# Supplementary material for: Molecular characterization of the RNA-protein complex directing −2/−1 programmed ribosomal frameshifting during arterivirus replicase expression
Source: J Biol Chem. 2021 Jan 13;295(52):17904–21. doi: 10.1074/jbc.RA120.016105 (PMC7939443; doi:10.1074/jbc.RA120.016105)
Supplement: Supplementary file 2 — Ankoor Patel [file mmc2.pdf]

Ankoor Patel

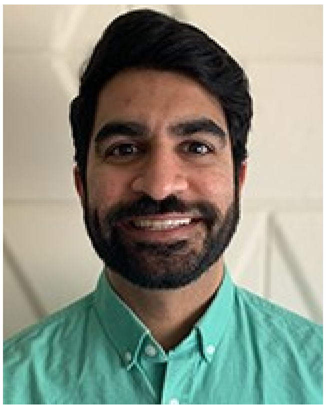

Current position: Ph.D. Candidate, Department of Microbiology, University of Manitoba, Winnipeg, Manitoba, Canada

Education: B.Sc. in Biochemistry, 2015, University of Manitoba, Winnipeg, Manitoba, Canada

---

How did you become interested in this topic?

Our lab focuses on a multitude of research disciplines from antibiotic resistance to human genetic disorders; however, my research focuses on viral proteases and how they are often multifunctional. I became interested in this field of research sheerly based on the juxtaposition of how viruses are incredibly simple yet complex at the same time. Even after years of studying viral proteases from several different viruses, it still amazes me how these proteins can carry out such a wide array of different processes in the host to benefit themselves. That is the case with PRRSVâ€™s nsp1Î², which acts primarily as an endopeptidase but has the ability to facilitate these remarkably intricate ribosomal frameshifting events to heighten pathogenesis.

Can you describe an exciting moment you experienced while doing this research?

Starting off in the lab as a junior scientist, I had a hard time trying to purify each protein needed for this research. The day I was successfully able to capture the tricomponent complex of both proteins bound to nucleic acid, I was elated and knew we really had something.

Where do you seek scientific inspiration?

Most often I seek scientific inspiration from my peers. Watching them succeed in doing what they love makes me feel that much more motivated to do the same in my little bubble of research.

Read Patel's article on page 17904.
